# Redox and Spin States Series of an Organometallic Heme Analogue Based on a Non-Innocent NHC/N-Donor Hybrid Macrocycle

**DOI:** 10.1021/acs.inorgchem.5c04599

**Published:** 2026-01-20

**Authors:** Massimiliano Morganti, Jan C. Kruse, Sandeep K. Gupta, Sebastian Dechert, Serhiy Demeshko, Franc Meyer

**Affiliations:** † University of Göttingen, Institute of Inorganic Chemistry, Tammannstraße 4, 37077 Göttingen, Germany; ‡ University of Göttingen, International Center for Advanced Studies of Energy Conversion (ICASEC), Tammannstraße 6, 37077 Göttingen, Germany

## Abstract

Iron complexes of tetradentate macrocyclic ligands containing *N*-heterocyclic carbene (NHC) donors have been referred to as organometallic heme analogues, but they usually lack the redox noninnocence under oxidizing conditions that is characteristic of porphyrins. Here we report a novel NHC/N-donor hybrid macrocyclic ligand containing two *trans* NHC moieties, a pyridine and a redox active carbazolide fragment. Its Fe^II^, Fe^III^ and formal Fe^IV^ complexes have been isolated and comprehensively characterized, where UV/vis and ^57^Fe Mössbauer spectroscopies, SQUID magnetometry and density functional theory (DFT) calculations reveal that the latter are best described as Fe^III^ systems antiferromagnetically coupled to a carbazolide-based organic π-radical. Two different redox series are obtained depending on the axial ligands: nitriles such as MeCN give low-spin (LS) configurations of the metal ion, while in case of weakly coordinating solvents and triflate anions the iron adopts an intermediate-spin (IS) configuration; MeCN binding constants have been determined. As in other heme analogues with NHC-based macrocycles, the strong equatorial σ-donor character raises the energy of the Fe­(d_*x* ^2^–*y* ^2^_) orbital, making high-spin (HS) iron species inaccessible. The combined features of equatorial ligand redox noninnocence, restriction to LS/IS surfaces and tunability via the axial coligands makes this a promising platform for bioinspired reactivity such as the generation of reactive Fe/O_*x*_ intermediates.

## Graphical abstract

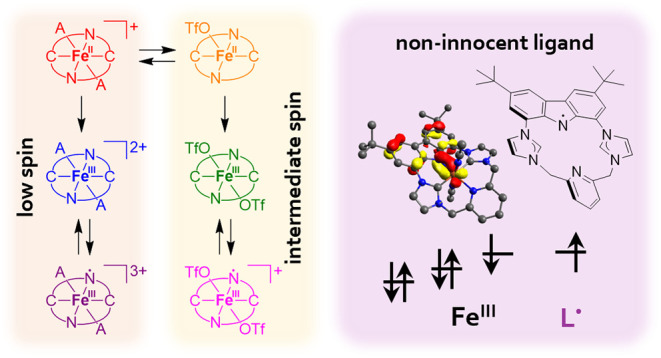

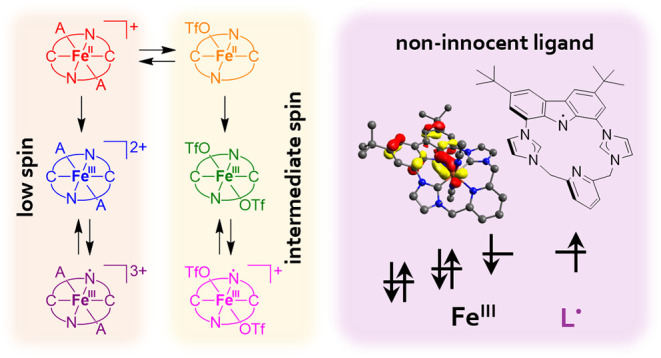

## Introduction

Heme and nonheme enzymes are attracting much interest due to their ability to catalyze an impressively wide range of interesting yet challenging chemical transformations.^1–4^ Therefore, scientists are devoting great efforts to the characterization of the active centers of such enzymes and to their emulation in synthetic model systems.^2,3,5–9^ However, many of the enzymatic intermediates involve iron in unusually high oxidation states,^5,10–13^ and the instability of the associated model complexes has been a long-time challenge for their isolation. In the last decades, the stabilization of key intermediates has been enabled by the development of new synthetic analogues based on sophisticated ligand design.^6,11,13^

The use of macrocyclic ligands containing N-heterocyclic carbenes (NHCs) instead of more common {N_4_}-donor macrocycles such as porphyrins or cyclams provided a new way to stabilize metals in unusual oxidation states, thanks to the combination of strong σ-donating character, variable π-acceptor ability, and easily tunable electronic properties of NHCs.^14–19^ Indeed, iron complexes of tetra­(NHC) macrocycles have been described as organometallic heme analogues.^19–21^ In 2013, our group reported an Fe^II^ complex of a macrocyclic tetra­(NHC) ligand (**A**, Figure 1) that can be readily oxidized with 2-(*tert*-butylsulfonyl)­iodosobenzene to afford the first organometallic oxidoiron­(IV) complex.^22^ It has been shown that the strong equatorial ligand field provided by the imidazol-2-ylidene donors raises the energy of the d_*x* ^2^–*y* ^2^_ orbital above the d_*z* ^2^_ orbital, which leads to a unique triplet state-only reactivity in C–H bond activation reactions, in contrast to the two-state reactivity postulated for other oxidoiron­(IV) model complexes.^23,24^ Furthermore, the same tetra­(NHC) platform gave rise to a series of structurally characterized {FeNO}^*n*^ complexes (*n* = 6,7,8) whose electronic structure is distinct from their heme analogues,^25^ and related tetra­(NHC) ligand scaffolds allowed for the synthesis of an {FeNO}^7^ complex,^20^ as well as catalytic aziridination reactions.^26–28^ More recently, NHCs have been combined with amines, amides, phosphines, alkoxides and pyridine in hybrid macrocycles, to evaluate the effect of the different donor sets on the electronic structures and reactivities of the resulting metal complexes.^16,29^ Complex **B**, for example, contains a hybrid ligand bearing two NHC and two pyridine moieties *trans* to each other.^30^

**Figure 1:**
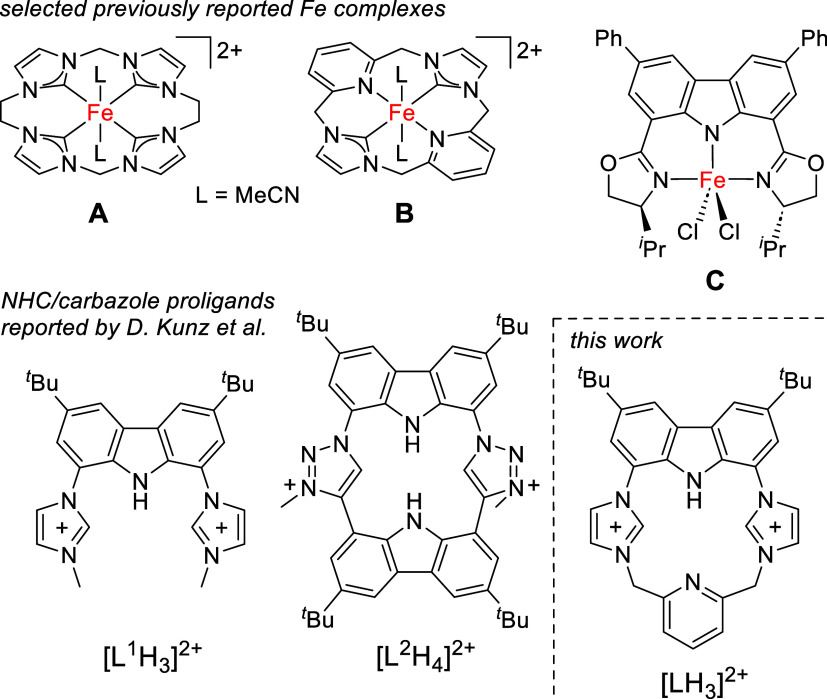
Examples of relevant nonheme iron complexes bearing a tetracarbene ligand scaffold (A),^22^ a hybrid NHC/pyridine macrocycle (B)^30,30^ and a redox non-innocent carbazole-based ligand (C);^36^ carbazole/imidazolium^38,39^ and carbazole/triazolium proligands^47^ developed by Kunz et al. and new {NCNC} hybrid ligand reported in this work.

Many heme enzymatic intermediates benefit from the participation of the non-innocent porphyrin ligand in the catalytic cycles to facilitate multielectron redox processes, as in Compound I (Cpd-I) of heme oxygenases.^2,12,31–33^ The ability to include redox-active ligands into bioinspired catalysts is indeed an active field of current research.^31,34,35^ A notable example of an asymmetric epoxidation nonheme iron catalyst that exploits a redox-active ligand is complex **C** (Figure 1), reported by Nakada and co-workers.^36^ This carbazole-based Fe^III^ complex can be oxidized in situ with iodosobenzene to generate a species with an oxidoiron­(IV) unit coupled to a carbazole-based π-radical cation, which can efficiently mediate asymmetric epoxidation thanks to the chiral directing groups on the BOX moieties. Pincer ligands based on a central carbazolide with flanking donor groups are now finding increasing use.^37^ As a prominent example, Kunz and co-workers have equipped a carbazole backbone with two imidazolium groups in proligands such as [L^1^H_3_]^2+^,^38,39^ and after 3-fold deprotonation the resulting tridentate {CNC} pincer ligands have been used for the synthesis of a variety of transition metal complexes and their catalytic applications.^40–45^ Related carbazole/triazolium {CNC} pincer ligands have been used in coinage metal complexes that exhibit fluorescence properties.^46^ Most relevant for the present work is the macrocyclic carbazole/triazolium proligand [L^2^H_4_]^2+^ that has been described as an NHC-containing porphyrinoid, and that serves as a carbenaporphyrin ligand in its lithium and scandium complexes.^47^

Apart from the use of redox-active ligands, heme and nonheme enzymes maximize their catalytic activity and selectivity by tuning the spin state of their active centers.^48–50^ Numerous heme models have been synthesized and studied to unravel the effects that allow for spin-state variations in active centers, such as hydrogen bonding, macrocycle deformation and axial ligation.^51–54^ Spin state variations governed by axial coligands have also been observed for Fe^II^ and Fe^III^ complexes of tetra­(NHC) macrocycles.^55,56^

Herein, we report the synthesis of a new macrocyclic {NCNC} hybrid proligand [LH_3_]^2+^, which after deprotonation combines the strong σ-donation of two *trans* NHC groups with a pyridine and a single redox-active carbazole moiety. This novel macrocycle is shown to form Fe^II^ complexes in both low- and intermediate-spin states that can undergo stepwise oxidation to the corresponding Fe^III^ congeners and further, via a second oxidation that is mostly ligand based, to the low- and intermediate-spin Fe^III^/π-radical cation coupled systems, thereby emulating some fundamental features of heme cofactors.

## Results and Discussion

### Synthesis and Characterization of the Hybrid Proligand [LH_3_]­(OTf)_2_

3,6-Di-*tert*-butyl-1,8-bis­(imidazole-1-yl)­carbazole (**1**) was synthesized in 75% yield according to literature.^39^ Macrocyclization of **1** with equimolar amounts of 2,6-bis­(bromomethyl)­pyridine in refluxing acetonitrile, followed by counteranion exchange by the addition of AgOTf gave the target hybrid proligand [LH_3_]­(OTf)_2_ in 39% yield (Scheme 1).

**Scheme 1:**
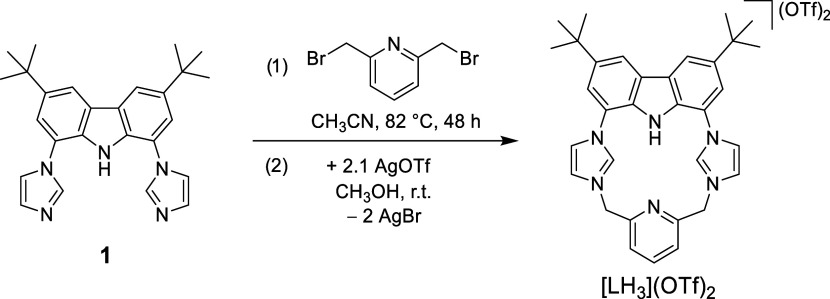
Schematic Representation of the Synthesis of [LH_**3**_]­(OTf)_**2**_

ESI­(+)-MS analysis of a CH_3_CN solution of [LH_3_]­(OTf)_2_ shows three major peaks at *m*/*z* = 665, 515, and 258 corresponding to ions [LH_3_(OTf)+H]^+^, [LH_3_]^+^ and [LH_3_]^2+^, respectively (Figure S1). Colorless single crystals suitable for X-ray diffraction (XRD) were obtained by slow diffusion of Et_2_O into a CH_3_CN solution of [LH_3_]­(OTf)_2_ at room temperature. The molecular structure of the dication core is depicted in Figure 2. The macrocycle adopts a concave (bowl-shaped) conformation, with the two imidazolium rings and the pyridine unit bending out from the plane of the carbazole backbone, with interplanar angles of 56.7, 49.7 and 38.1° respectively (Figure 2, right). This structural feature is also found in the reported solid state structures of doubly *N*-substituted ligands derived from **1**, in which the constraint of the carbazole backbone and the steric interaction between the imidazolium C6/6′ protons force the bending of the imidazolium rings out from the carbazole plane.^39^ However, the ^1^H NMR spectrum of [LH_3_]­(OTf)_2_ recorded in DMSO-*d*_6_ at room temperature shows nine signals including a singlet for the methylene linkages, indicative of apparent C_2v_ symmetry of the molecule due to fast dynamic behavior in solution. The complete set of NMR spectra with peak assignments as well as IR and UV/vis spectra of [LH_3_]­(OTf)_2_ are shown in Figures S29–S36, S46 and S2, S3 in the Supporting Information.

**Figure 2:**
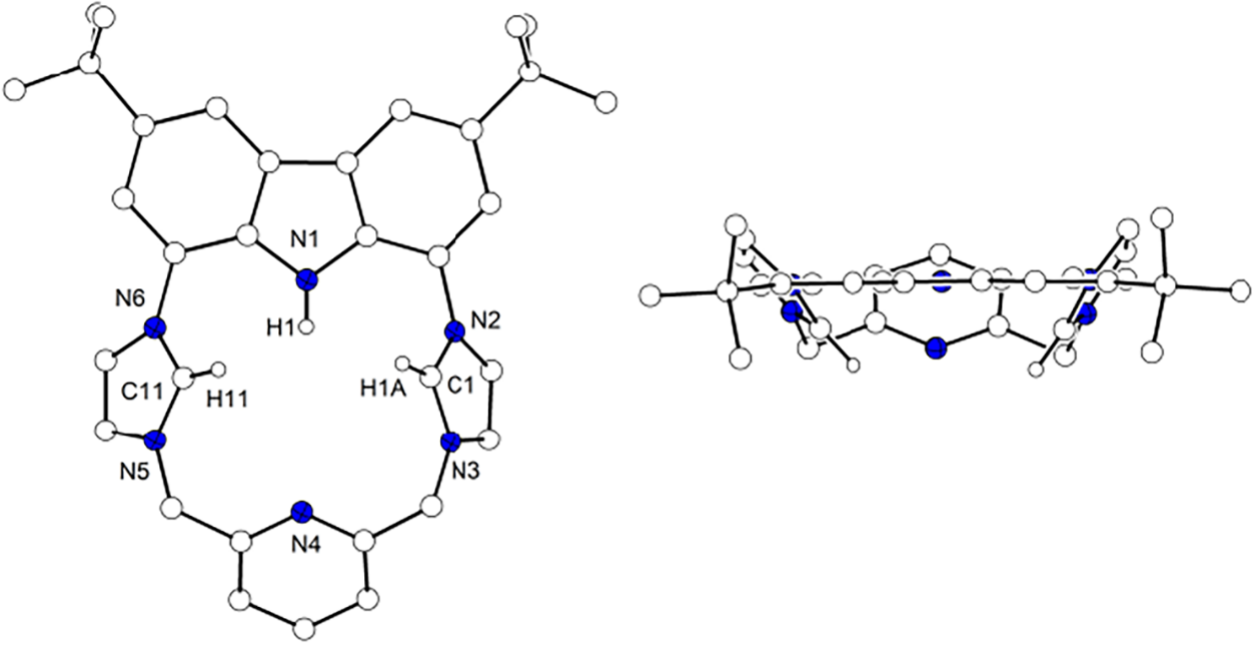
Molecular structure of the cationic part of [LH_3_]­(OTf)_2_. Most hydrogen atoms are omitted for clarity. Left: view perpendicular to the carbazole plane; right: view along the carbazole plane.

### Synthesis and Characterization of Ferrous Complexes of [LH_3_]­(OTf)_2_

The synthesis of an Fe^II^ complex of [LH_3_]­(OTf)_2_ was achieved by using {Fe­[N­(SiMe_3_)_2_]_2_}_2_ ({Fe­(hmds)_2_}_2_) as an iron source that is also capable of deprotonating the ligand (Scheme 2).^57^ Treatment of [LH_3_]­(OTf)_2_ with 1 equiv of {Fe­(hmds)_2_}_2_ in CH_3_CN initially formed a yellow precipitate, which slowly dissolved over time, affording a red-brown solution. After workup, complex [LFe^II^(MeCN)_2_]­OTf (**2a**) was isolated as an orange powder in 73% yield. Slow diffusion of Et_2_O into a concentrated solution of **2a** in CH_3_CN afforded orange single crystals suitable for X-ray diffraction; the molecular structure of the cation is shown in Figure 3 and selected bond lengths and angles are listed in Table 2 (vide infra).

**Figure 3:**
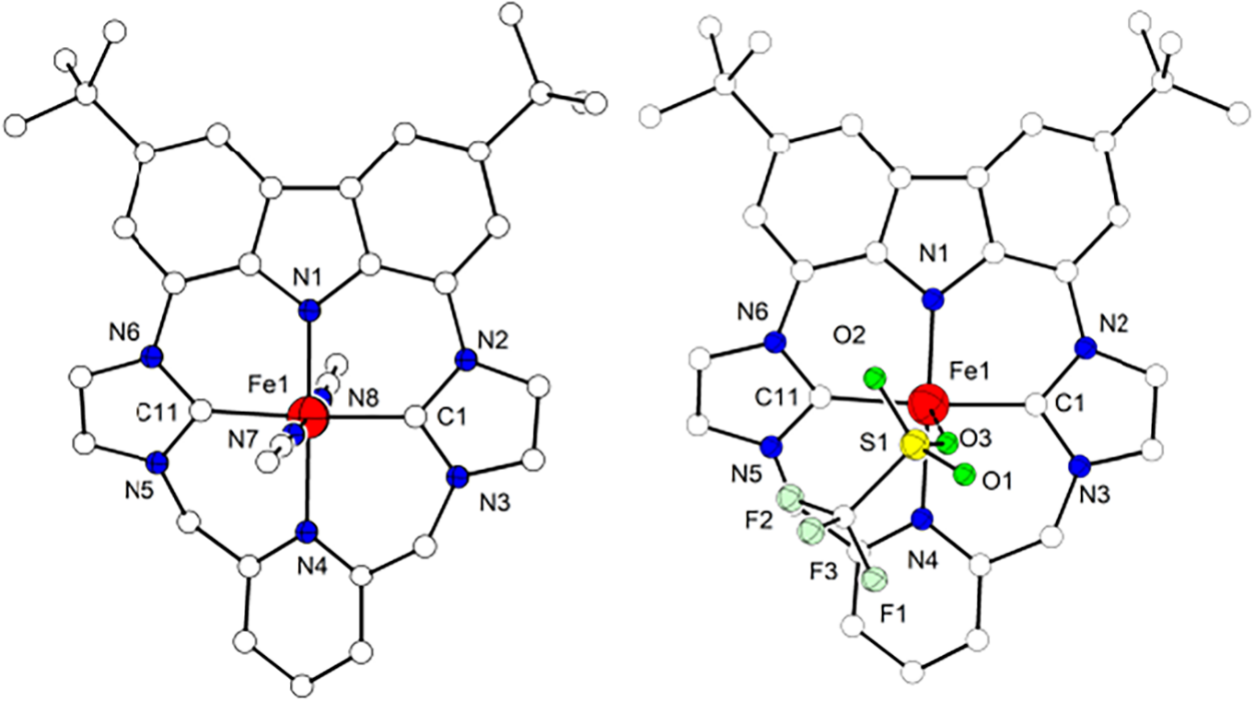
Molecular structures of the cations of **2a** (left) and of **2b** (right). Hydrogen atoms are omitted for clarity.

**Scheme 2:**
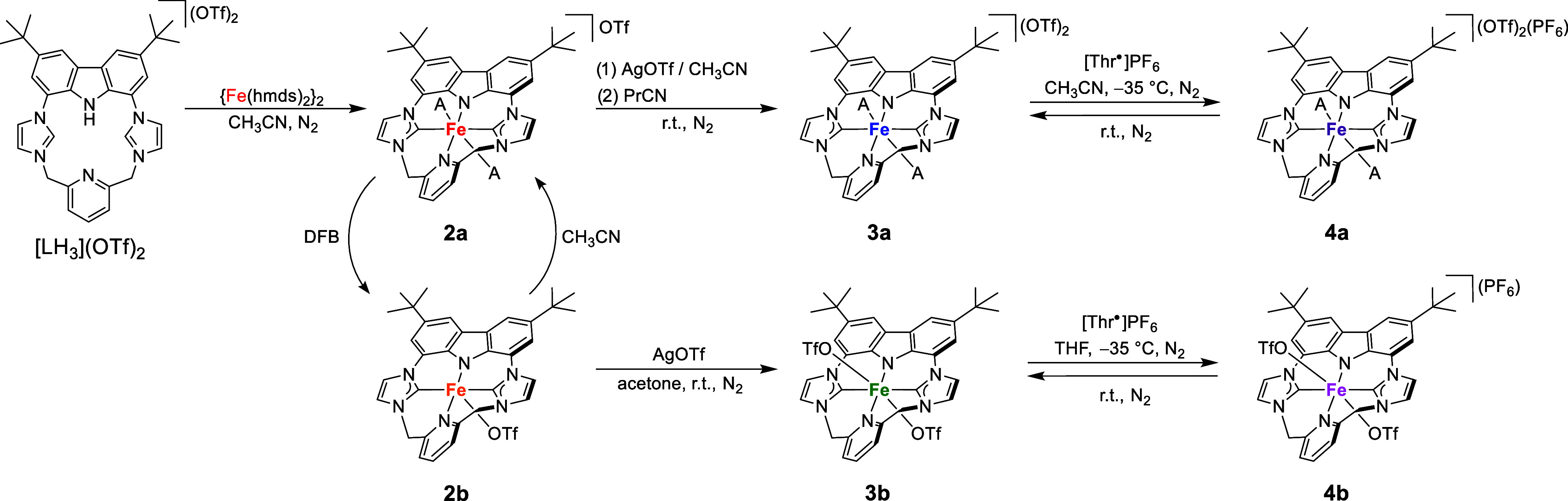
Synthesis of the Ferrous Complexes **2a** and **2b**, Their Interconversion and Their Oxidations to **3a/3b** and **4a/4b**. Ligands Denoted as A Represent Coordinated Nitrile Solvent Molecules (MeCN or PrCN).

Complex **2a** crystallizes in the monoclinic *P*2_1_/*c* space group; the metal ion is found six-coordinate, with the macrocyclic {NCNC} ligand L^–^ occupying the equatorial sites and two axial MeCN molecules located *trans* to each other. The L^–^ macrocycle adopts a twisted conformation, in which the two imidazol-2-ylidene rings and the pyridine fragment form angles of 21.1, 25.1 and 35.8° with the carbazole plane, the latter being 3.9° distorted from full planarity. Despite this deformation of the macrocycle, the ferrous ion maintains an almost perfect octahedral {FeC_2_N_4_} coordination geometry, with an overall deviation parameter Σ of 18.1.^58^ Fe–C bond lengths (1.941(2) and 1.942(2) Å) fall within the range of known Fe^II^ complexes with NHC/pyridine hybrid ligands (1.80–2.16 Å)^59–66^ and are slightly longer compared to the Fe–C bonds in complex **B**.^30^ Also the Fe–N^py^ bond length of 2.071(2) Å is not unusual when compared with the other NHC/pyridine hybrid systems (1.8906–2.279 Å).^59–66^ However, the Fe–N^cbz^ bond is the shortest (1.938(2) Å) among the carbazole-Fe^II^ complexes characterized so far (1.958–1.980 Å).^67–70^ A possible explanation for this relatively short Fe–N^cbz^ distance could be a marked π-donation from the carbazole, combined with the π-acceptor abilities of the pyridine in *trans* position.^71^ Similar M–N bond lengths were found for, e.g., a low-spin octahedral pyrrole-based pincer Fe^II^ complex and some of its CO-ligated derivatives^72^ and in a Co^II^ complex with a carbazole-bis­(imine) ligand.^73^ Moreover, due to the anionic nature of the carbazolide a significant electrostatic contribution to the Fe–N^cbz^ bonding compared to the other Fe–L bonds can be expected.^74^

The ^1^H NMR spectrum of crystals of **2a** dissolved in CD_3_CN, recorded at r.t., shows apparent C_2*v*_ symmetry of the molecule in solution, as evidenced by the presence of one singlet for the two methylene linkers of the macrocycle, and two signals for the *meta*- and *para*-protons of the pyridine moiety (Figure 4). This finding indicates that the twisting of the L^–^ macrocycle is not static, and it suggests a fast dynamic process associated with flipping of the pyridine around a bisecting noncrystallographic *C*_2_ axes. Furthermore, all the signals appear in the range of 0–12 ppm, indicative of a diamagnetic, low-spin (*S* = 0) nature of **2a**. The ^13^C NMR spectrum shows a typical resonance at 202.2 ppm for the deshielded C2 atoms of the imidazol-2-ylidene groups.^75^ All other resonances appear just slightly shifted compared to proligand [LH_3_]­(OTf)_2_. Full 2D-NMR spectra and signal assignments are shown in Figures S37–S44 in the Supporting Information.

**Figure 4:**
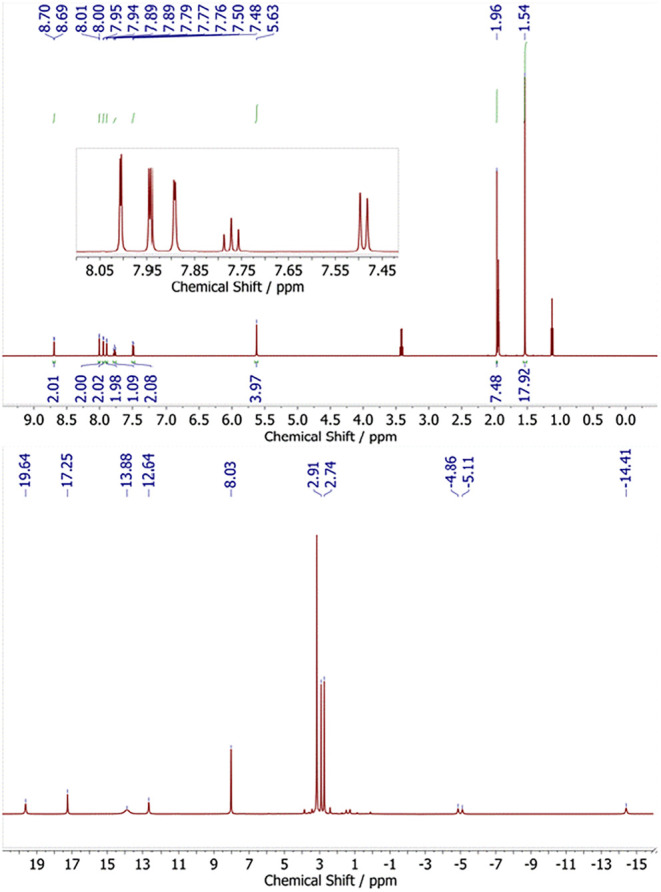
^1^H NMR spectra of **2a** dissolved in CD_3_CN (top) and in DMF-*d*_7_ (bottom) at r.t.

Interestingly, a solution of crystalline **2a** in DMF-*d*_7_ gave a ^1^H NMR spectrum of a paramagnetic species shown in Figure 4 (bottom). Similar spectra could be also obtained when dissolving **2a** in THF-*d*_8_ or CD_3_OD. When these solvents were removed under vacuum and the orange residue was redissolved in CD_3_CN, the ^1^H NMR spectrum of diamagnetic complex **2a** is regained. Moreover, when crystals of complex **2a** were kept under vacuum for several hours, a dramatic change in the ^57^Fe Mössbauer spectrum was observed (vide infra), which was reversed when the obtained solid was redissolved in CH_3_CN and again precipitated with Et_2_O (Figure S53).

These findings strongly suggested that the spin state of **2a** can be reversibly tuned by changing the coordination environment of the complex, e.g., by replacing the solvent molecules occupying the two axial positions. When purification of complex **2a** was carried out with exclusive usage of less coordinating solvents (THF, DCM, 1,2-difluorobenzene (*o*-DFB)), or when pure **2a** was repeatedly dissolved and reprecipitated from such media, the loss of axially coordinated CH_3_CN molecules led to the isolation of the pentacoordinated complex [LFe^II^(OTf)] (**2b**). Concentration of a saturated solution of this ferrous compound in *o*-DFB led to the formation of single crystals suitable for XRD.

**2b** crystallizes in the monoclinic *P*2_1_/*c* space group, and the structure in solid state confirms the loss of both axial CH_3_CN ligands (Figure 3). In **2b** the Fe^II^ has a square pyramidal coordination geometry (τ_5_ is 0.003),^76^ with a triflate anion in the axial position. The hybrid macrocycle adopts a twisted conformation similar to the one in **2a**, and the metal ion is now located 0.10 Å above the {NCNC} plane. Fe–N^py^ and Fe–C distances are slightly longer than in **2a**, as expected for an intermediate spin state, while the Fe–N^cbz^ bond is slightly shorter (1.899(1) in **2b** vs 1.938(2) Å in **2a**); the axial Fe–O bond is very long (2.322(1) Å). Considering the overall structure of **2b** and the strong-field equatorial {NCNC} ligation, an *S* = 1 intermediate spin state can be predicted, as confirmed by magnetometry and spectroscopic methods (vide infra). According to DFT calculations, the two singly occupied molecular orbitals of **2b** are localized mainly on Fe (Figure S84), whereas the doubly occupied HOMO of **2a** has significant Fe–N^cbz^ antibonding character (Figure S77), which is reflected in the relative contraction of the Fe–N^cbz^ bond in intermediate-spin complex **2b**.

When **2a** is dissolved in PrCN it shows a UV/vis absorption spectrum distinct from that of **2b** dissolved in THF, (see the following section for their comparison). Both maintain their different electronic structures (*S* = 0 vs *S* = 1) at low temperatures (Figures S6 and S15). Titration experiments were carried out by adding aliquots of MeCN to a THF solution of **2b** in order to determine the formation constant *K*_2_ of the MeCN complex **2a** (Figure 5). Intermediates with only one nitrile ligand were not observed to a significant extent in the UV/vis spectra (Figure S16). Across the temperature range investigated, *K*_2_ was found to increase by more than one order of magnitude with decreasing temperature, as expected for an entropically disfavored ligand binding. An additional titration experiment was carried out in acetone solution at 20 °C, in which *K*_2_ was approximately doubled when compared to the value in THF solution (Table 1).

**Figure 5:**
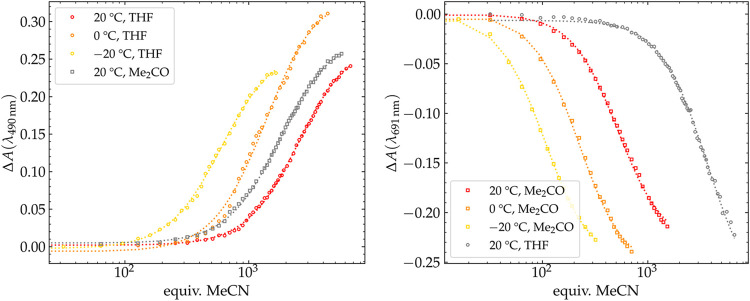
Plots of the absorbance difference at 490 or 691 nm for 0.1 mm solutions of **2b** (left) and **3b** (right), respectively, in acetone or THF versus added amount of CH_3_CN and fitted 1:2 binding isotherms at different temperatures, see SI for details).

**Table 1:** Equilibrium Constants K_2_/L^2^·mol^–2^ for the Coordination of MeCN to **2b** and **3b** in Acetone or THF at Different Temperatures

	**2b**		**3b**	
*T*/°C	THF	acetone	THF	acetone
20	14.4	31.2	7.56	392
0	50.7	n.d.	n.d.	2.31 × 10^3^
–20	272	n.d.	n.d.	1.17 × 10^4^

### Electrochemical Properties of **2a** and **2b**

The redox properties of complexes **2a** and **2b** were probed by (spectro)­electrochemistry experiments. For cyclic voltammetry (CV) measurements, a glassy carbon working electrode was used, and all data were referenced versus the internal standard ferrocenium/ferrocene (Fc^+/0^). **2a** was dissolved in 0.1 m (^*n*^Bu_4_N)­PF_6_ in dry and degassed CH_3_CN, while **2b** was dissolved in 0.2 m ( ^*n*^Bu_4_N)­PF_6_ in dry and degassed THF. **2a** shows rich redox behavior, with two facile oxidations at −0.37 and +0.62 V, and two irreversible reductions at −2.00 and −2.31 V (Figure 6). The two oxidation processes are reversible, as evidenced by the linear dependence of the peak current on the square root of the scan rate and by the peak-to-peak separation of 74 and 81 mV, respectively (Figures S57–59). The first oxidation is assigned to the Fe^III^/Fe^II^ couple, as confirmed by ^57^Fe Mössbauer spectroscopy, which will be discussed in detail in the following section. This oxidation potential is cathodically shifted compared to those of Fe^II^ complexes of tetra­(NHC) macrocycles such as **A** or NHC/pyridine hybrid ligands such as **B**,^30,55,59^ where the Fe^III^/Fe^II^ potentials are found in the range of −0.16 to +0.76 V, which may be rationalized by the lower charge of **2a**.^54,77–79,27^ However, the Fe^III^/Fe^II^ potential of **2a** is significantly less negative than those of tetra­(NHC) ligated iron complexes with a σ- and π-donating axial thiolato ligand.^56^ The second oxidation process of **2a** at +0.62 V can be attributed to a ligand-centered oxidation, as it has previously been shown that many 3,6-disubstituted carbazoles are oxidized to their respective radical cations at similar potentials.^81,82^ To support these hypotheses on the nature of the oxidized products, UV/vis spectroelectrochemical analyses (UV-SEC) were performed on MeCN solutions of **2a**.

**Figure 6:**
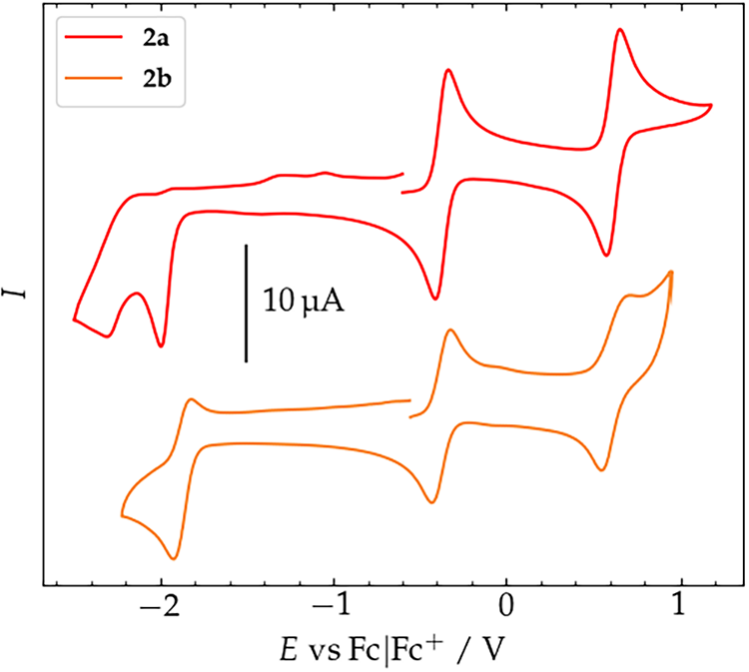
Cyclic voltammograms of 1 mM solutions of crystalline **2a** in MeCN with 0.1 m (^*n*^Bu_4_N)­PF_6_ at a scan rate of 0.1 V/s (red, top) and **2b** in THF with 0.2 m (^*n*^Bu_4_N)­PF_6_ at 0.8 V/s (orange, bottom) at r.t.

The initial spectrum of complex **2a** shows three main bands at 321, 377, and 480 nm (full spectrum is shown in Figure S4). When applying a potential of −0.14 V vs Fc^+/0^ to induce the first oxidation process (Figure 7a), a new species **3a** is generated, with absorptions at 338, 419, 446, 560, 602 nm and a broad band in the NIR-region. The presence of isosbestic points indicates a clean one-step conversion upon oxidation. The initial spectrum of **2a** was re-established when applying a potential of −0.90 V, indicating the full chemical reversibility of this redox process (Figure S67). The spectral changes for the second oxidation event to give twice oxidized **4a** were monitored by applying a potential of 0.76 V. As shown in Figure 7b, three new major absorptions are observed at 511, 728, and 804 nm, the latter two bands showing a rather high intensity, suggesting that they originate from charge transfer transitions. Indeed, these spectroscopic features are reminiscent of those of carbazole dimer and poly-*N*-vinylcarbazole radical cations, where two intense charge resonance bands between 700 and 1000 nm were observed.^83,84^ Furthermore, similar charge transfer bands have been reported for a Fe^III^–porphyrin π-radical cation complex,^85^ and Nakada’s Fe^III^-carbazole-bisoxazoline based catalyst **C** after oxidation with iodosobenzene.^36^

**Figure 7:**
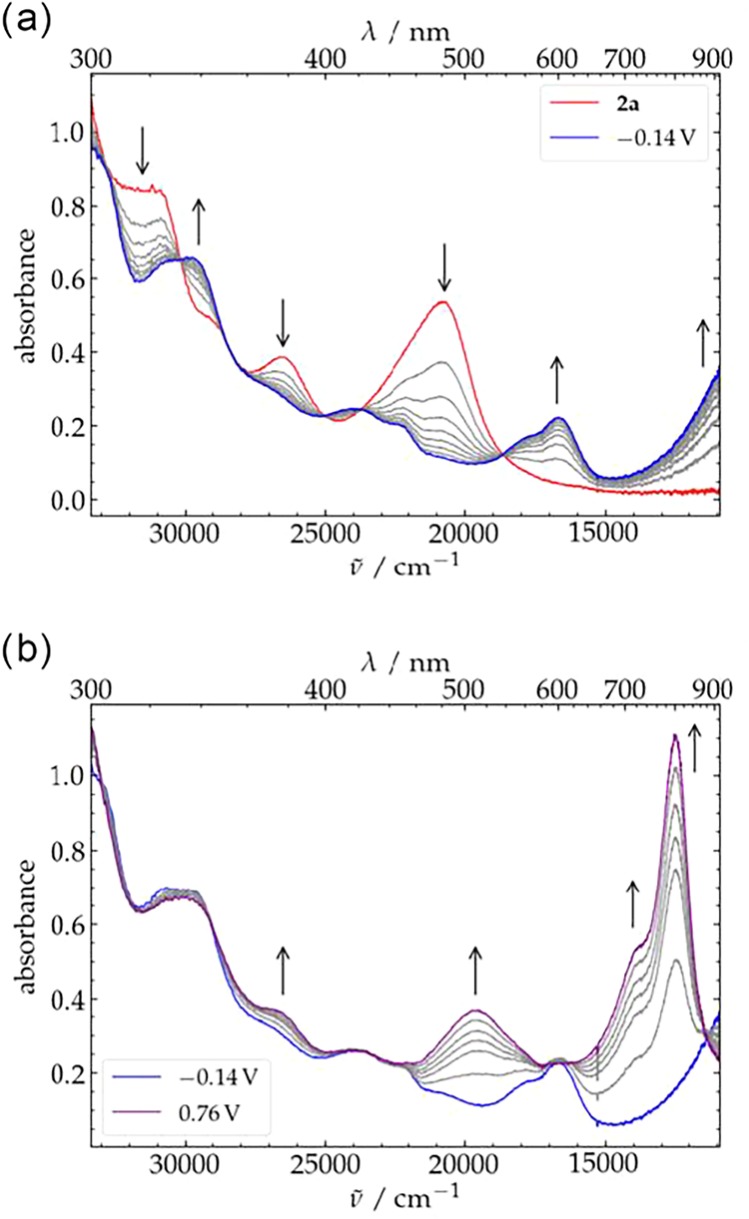
UV/vis spectroelectrochemistry of a 1 mm solution of complex **2a** in MeCN with 0.1 m (^*n*^Bu_4_N)­PF_6_ as electrolyte. (a) first oxidation of **2a** (red spectrum) at an applied potential of −0.14 V vs Fc^+/0^ held for 300 s; (b) second oxidation of the mono-oxidized complex (blue spectrum) at an applied potential of 0.76 V vs Fc^+/0^ held for 300 s.

Subsequent application of a potential of −0.14 V for 5 min partially restored the original spectrum of **3a** (Figure S68), indicating partial chemical reversibility of the process (the doubly oxidized complex **4a** decomposes over time in solution, see below). Full assignment of the optical features of all the complexes was performed by TD-DFT calculations (vide infra).

Analogous electrochemical signatures were found in the CV of complex **2b** recorded in THF (Figure 6). The two reversible oxidations occurred at potentials very close to those of **2a** in MeCN (−0.36 and 0.66 V) (Figures S62–64), while the first reduction event appeared at a less negative potential (−1.88 V) and showed partial reversibility. All in all, complexes **2a** and **2b** show very similar electrochemical behavior despite the differences in axial ligation and spin state, opening the interesting possibility to isolate two analogous redox series of Fe complexes of the {NCNC} macrocycle that differ in their spin ground states.

**Figure 8:**
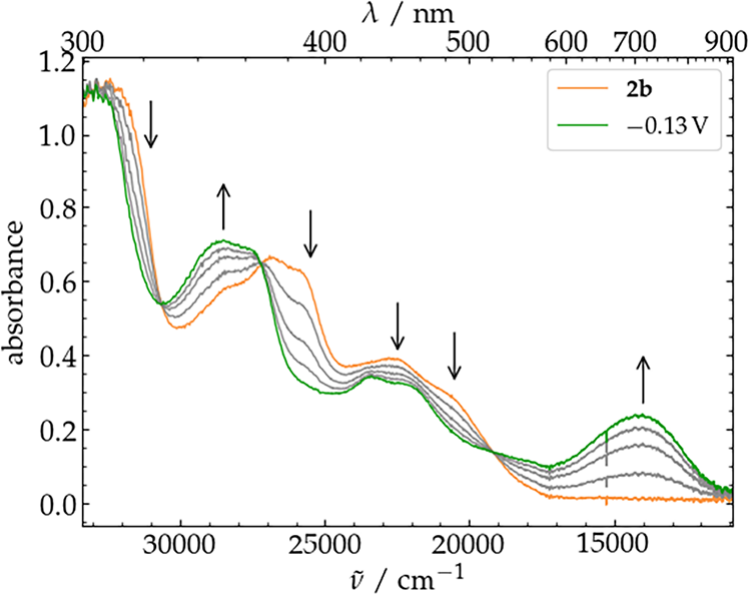
UV/vis spectroelectrochemistry of a 1 mm solution of complex **2b** in THF with 0.1 m (^*n*^Bu_4_N)­PF_6_ as electrolyte showing the first oxidation of **2b** (orange spectrum) at an applied potential of −0.13 V vs Fc^+/0^ held for 2700 s.

The UV/vis spectrum of **2b** recorded in THF shows main features at 305, 355, 373, 388, 436, and 484 nm (for the full spectrum see Figure S13). When applying a potential of −0.13 V, bands of a new species **3b** appear at 351, 427, 450 nm together with a broad feature at 713 nm and two isosbestic points at 368 and 521 nm (Figure 8). Rereduction at a potential of −0.53 V re-established the original spectrum of **2b** (Figure S69). Interestingly, the broad feature between 600 and 800 nm appears at higher energy compared to **2a**, indicating a significant difference in the electronic structure of the two ferric complexes **3a** and **3b**, also suggesting a different ligand environment. Unfortunately, attempts of monitoring the spectroelectrochemical oxidation to the 2e^–^ oxidized species **4b** at higher potentials were unsuccessful and no further spectral changes could be observed, indicating a high instability of the target complex in THF at r.t. (**4b**, vide infra).

Motivated by the rich redox chemistry of both complexes and the possibility of both metal- and ligand-based oxidations, we targeted the bulk synthesis, isolation, and full characterization of the four oxidized complexes.

### Syntheses of the One- and Two-Electron Oxidized Complexes **3a**, **3b**, **4a** and **4b**

Chemical one-electron oxidation of **2a** to **3a** was achieved by using AgOTf as oxidant (formal potential of 0.04 V vs Fc^+/0^ in MeCN).^86^ XRD quality single crystals of [LFe^III^(PrCN)_2_]­(OTf)_2_ (**3a**) were obtained via slow diffusion of Et_2_O into a concentrated PrCN solution at r.t. Oxidation of **2b** to **3b** was achieved in an analogous way using acetone as solvent. Single crystals of [LFe^III^(OTf)_2_] (**3b**) were grown via layering Et_2_O on top of a concentrated acetone solution at r.t. The second oxidation of **3a** to **4a** was performed using thianthrene radical cation hexafluorophosphate ([Thṙ]­PF_6_) in MeCN (0.86 V vs Fc^+/0^)^86^ at −35 °C (Scheme 2). Similarly, **4b** was obtained from **3b** via oxidation with [Thṙ]­PF_6_ at −35 °C using THF as a solvent. Even at −35 °C, **4a** in MeCN gradually decomposes within a few hours and complex **3a** is obtained as a major product (Figure S12). Similarly, gradual decomposition of **4b** in acetone or THF mostly returns **3b**.

### Molecular Structures of **3a**, **3b** and **4a** in Solid State

Single crystal XRD analysis of **3a** (triclinic *P*1̅ space group) confirmed the presence of an oxidized dicationic complex (Figure 9, and Table 2); the overall structure of the cation is very similar to the one of **2a**, with the metal ion in octahedral environment (Σ parameter of 21.91) composed of the equatorial {NCNC} macrocycle and two axial MeCN ligands. Fe–C bonds (1.958(3) and 1.956(3) Å) and the Fe–N^py^ bond (2.104(3) Å) are longer than in ferrous **2a**, suggesting reduced π-backdonation to the imidazol-2-ylidene and pyridine moieties.^55^ In contrast, the Fe–N^cbz^ bond in **3a** (1.859(3) Å) is shorter than in **2a** (1.938(2) Å) and also shorter than in reported Fe^III^-carbazole complexes.^36,71^ A similar shortening of the Fe–N bond was observed in highly distorted porphyrin Fe^III^ complexes with π-accepting axial ligands, in which the ruffling of the macrocycle, in a shape similar to the one of complexes **2a** and **3a**, allows for interaction between the singly occupied Fe­(d_*xy*_) orbital with ligand orbitals of the same symmetry (for a (d_*xz*_,d_*yz*_)^4^(d_*xy*_)^1^ configuration that results from the stabilization of the d_*xz*_ and d_*yz*_ orbitals by the π-accepting axial ligands), giving rise to delocalization of the unpaired electron on the nitrogen atom of the pyrrole rings.^54^ A similar delocalization can be evidenced for complex **3a** by DFT calculations (vide infra), where the axial MeCN ligands lead to a (d_*xy*_)^2^(d_*xz*_,d_*yz*_)^3^ configuration and the d_*xz*_ orbital then interacts with the N­(p_*z*_) orbital in the ligand. This reflects a pronounced covalent character of the Fe–N^cbz^ π bond and provides an explanation for its rather short bond length.

**Table 2:** Selected Bond Lengths and Angles Determined by Single Crystal X-ray Diffraction and Corresponding Values of BP86 Optimized Geometries (in *italics*) of Complexes **2a/2b, 3a/3b, 4a/4b**

	**2a**	**2b**	**3a**	**3b**	**4a**	**4b**
	Bond Length exp./*BP86 opt*. (Å)					
Fe–N^cbz^	1.938(2)/*1.937*	1.899(1)/*1.888*	1.859(3)/*1.881*	1.884(3)/*1.882*	1.837(3)/*1.862*	*1.849*
Fe–N^py^	2.071(2)/*2.049*	2.100(1)/*2.077*	2.104(3)/*2.089*	2.133(3)/*2.129*	2.087(3)/*2.116*	*2.299*
Fe–C	1.941(2)/*1.938*	1.944(2)/*1.926*	1.956(3)/*1.940*	1.976(2)/*1.956*	1.944(3)/*1.947*	*1.955*
Fe–C	1.942(2)/*1.938*	1.943(2)/*1.916*	1.958(3)/*1.940*	1.976(2)/*1.955*	1.942(3)/*1.945*	*1.955*
Fe–A_1_	1.934(2)/*1.874*	2.322(1)/*2.192*	1.950(6)/*1.894*	2.226(2)/*2.201*	1.917(3)/*1.901*	*2.268*
Fe–A_2_	1.938(2)/*1.875*		1.937(2)/*1.888*	2.226(2)/*2.200*	1.921(3)/*1.901*	*1.911*
	Angle exp./*BP86 opt*. (°)					
N^cbz^–Fe–C	87.90(7)/*87.9*	88.40(6)/*88.1*	89.1(1)/*88.8*	88.22(7)/*88.5*	89.10(12)/*89.4*	*90.3*
N^cbz^–Fe–C	88.01(8)/*88.0*	88.25(6)/*88.6*	88.5(1)/*88.7*	88.22(7)/*88.5*	89.60(12)/*89.5*	*90.1*
N^py^–Fe–C	92.04(7)/*92.0*	91.12(6)/*91.0*	90.9(1)/*91.0*	91.78(7)/*91.5*	90.52(12)/*90.6*	*89.4*
N^py^–Fe–C	92.06(8)/*92.1*	91.62(6)/*91.9*	91.5(1)/*91.4*	91.78(7)/*91.5*	90.77(12)/*90.6*	*89.5*
N^cbz^–Fe–N^py^	179.9(7)/*179.9*	173.64(5)/*174.8*	179.2(1)/*179.8*	180.00/*179.8*	178.9(1)/*179.9*	*173.6*
C–Fe–C	175.9(1)/*175.9*	173.82(6)/*174.5*	177.4(2)/*177.6*	176.5(1)/*177.0*	178.7(1)/*178.9*	*172.7*
N_A1_–Fe–N_A2_	178.8(1)/*174.1*		172.5(4)/*178.4*	167.62(9)/*170.9*	175.8(1)/*175.9*	*176.8*

A1 and A2 refer to axial ligands: CH_3_CN for **2a** and **4a**, PrCN for **3a** and OTf^–^ for **2b** and **3b**. DFT calculated values for **4a** are given for the lowest single point energy configuration, with a low or intermediate spin Fe­(III) center antiferromagnetically coupled to a radical on the carbazole moiety (see DFT section).

**Figure 9:**
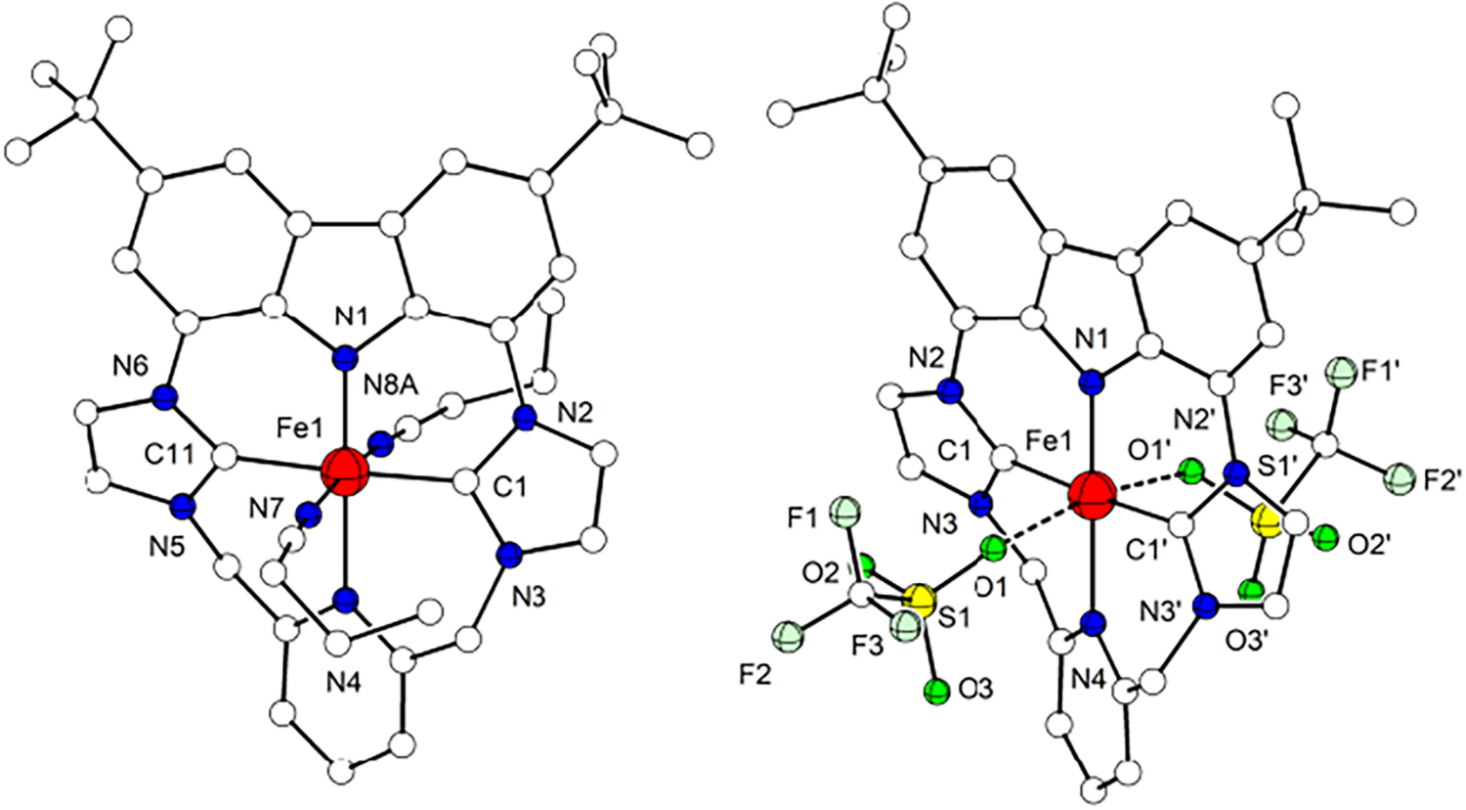
Molecular structure of the cation of **3a** (left) and of **3b** (right). Hydrogen atoms are omitted for clarity. Symmetry transformation used to generate equivalent atoms: (′) 1 – *x*, *y*, 3/2 – *z*.

Complex **3b** in solid state (monoclinic space group *C*2/*c*) also features octahedral geometry, but with two triflate anions in the axial positions and a Σ parameter of 39.30. All six bonds around Fe are longer compared to the ones in **3a**, compatible with a *S* = 3/2 ground state (vide infra).^87,88^ The complex has a crystallographic *C*_2_ axis passing through N1, Fe1 and N4. The axial Fe–O^OTf^ bonds of 2.226(2) Å are substantially elongated compared to the Fe–N^MeCN^ bonds in **3a**, which in combination with the weak field character of the triflate ions gives rise to the intermediate spin state, as confirmed by Mössbauer and EPR spectroscopies as well as SQUID magnetometry (vide infra). In this regard, complexes **3a** and **3b** would behave in the opposite way to reported five-coordinate octaethyltetraphenylporphyrin-Fe^III^ complexes, for which the use of weaker field axial ligands caused the shortening of the Fe–N^pyrrole^ bonds, and hence a spin state change from high-spin (when using Cl^–^ as axial ligand) to pure low-spin (using TfO^–^, ClO_4_^–^ and I_3_^–^), passing through spin-admixed species in case of intermediate field-strength axial ligands (I^–^).^53^

When the low-spin complex **3a** was further oxidized, the formally Fe^IV^ complex [LFe­(MeCN)_2_]­(OTf)_2_(PF_6_) (**4a**) was obtained, which crystallizes in the monoclinic space group *P*2_1_/*c* (Figure 10). The octahedral geometry of the cation with two axial MeCN ligands is preserved (Σ parameter of 20.66) while all Fe-ligand bonds are slightly shorter than in **3a** (Table 2). The C–C and C–N bond lengths within the carbazole moieties of the proligand [LH_3_]­(OTf)_2_ and the iron-containing compounds **2a, 2b**, **3a**, and **3b** show only small variations (Figure 11). In contrast, the bond lengths in **4a**, particularly within the six-membered rings, underline the presence of a ligand-based radical. Moreover, the bond lengths of the carbazole moiety observed for **4a** closely match those reported for the organic radical [L'H]­[Al­(OC_4_F_9_)_4_] (L'H = radical of 1,8-bis­(3,5-di-*tert*-butylphenyl)-3,6-di-*tert*-butyl-carbazole).^89^ The assignment of oxidation states in twice oxidized **4a** (as well as **4b**) was further supported by a combination of spectroscopy, magnetometry and DFT calculations, as discussed below.

**Figure 10:**
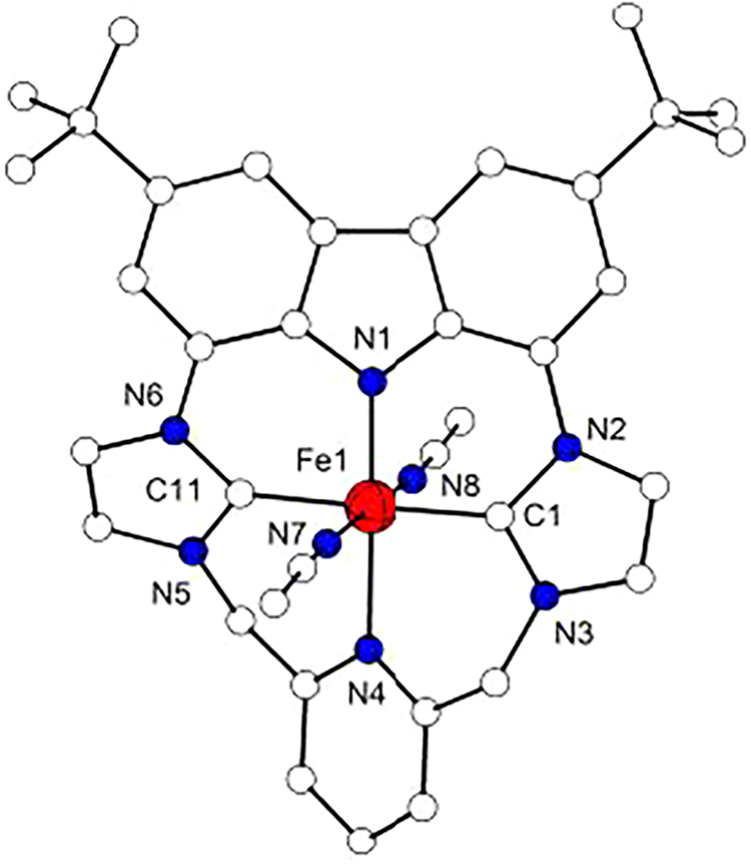
Molecular structure of the cation of **4a**. Hydrogen atoms are omitted for clarity.

**Figure 11:**
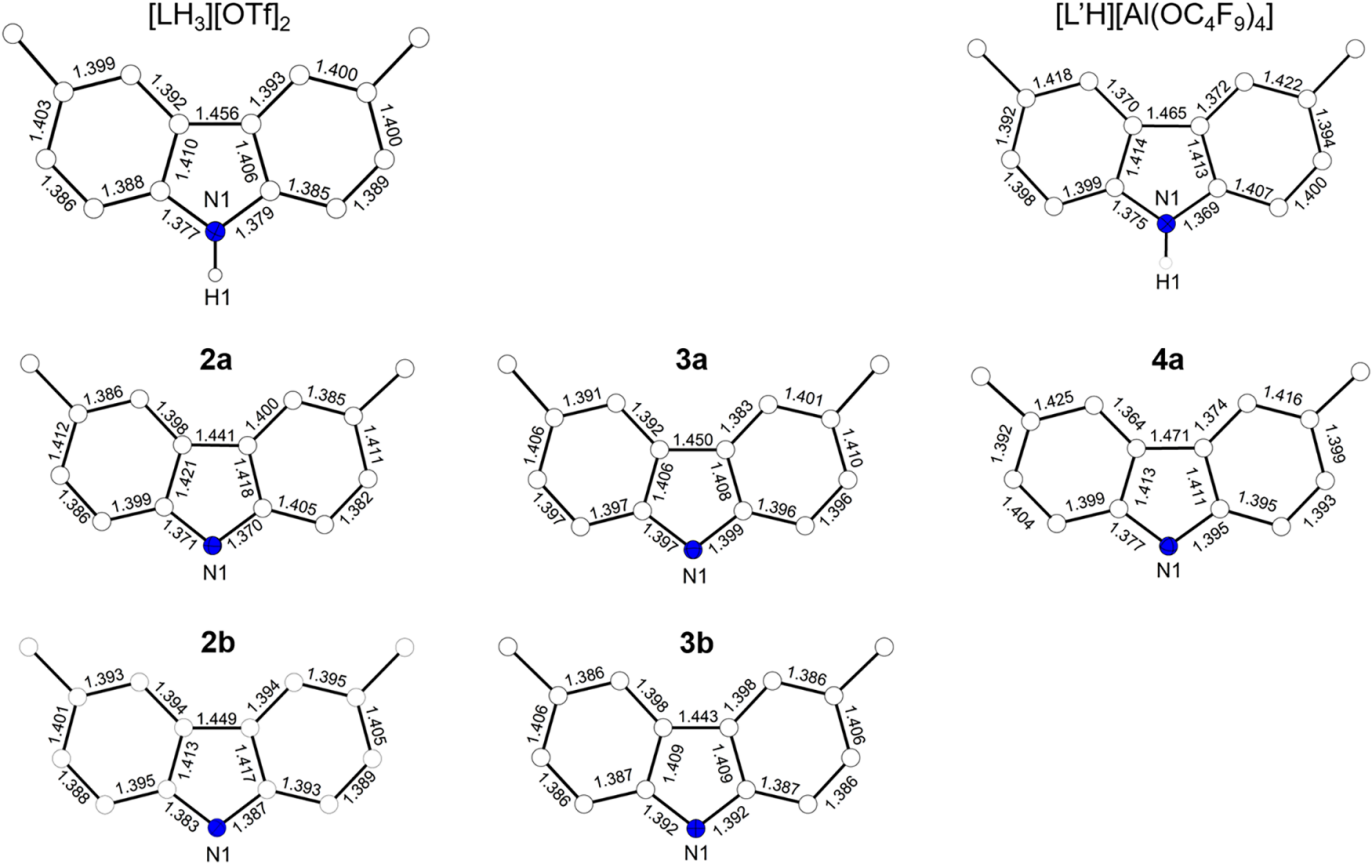
Comparison of the C–C and C–N bond lengths [Å] in the carbazole moieties of [LH_3_]­(OTf)_2_, **2a**, **2b**, **3a**, **3b**, **4a**, and [L'H]­[Al­(OC_4_F_9_)_4_], with [L'H]^+^ being the radical cation of 1,8-bis­(3,5-di-*tert*-butylphenyl)-3,6-di-*tert*-butyl-carbazole.^89^

### Electronic Structure Analysis of **2a**/**b** and **3a**/**b**: Modulating the Spin State via the Axial Ligands

To assess the effect of axial ligands on the electronic structures of the present redox series of ferrous and ferric complexes based on the {NCNC} macrocyclic ligand, viz. **2a**/**b** and **3a**/**b**, a bouquet of experimental techniques has been applied. The Mössbauer spectrum of complex **2a** (Figure 12-I) shows an isomer shift δ = 0.33 mm·s^–1^, in agreement with its low-spin Fe^II^ nature (Table 3). Slightly lower values were found for Fe^II^-tetracarbene complexes such as **A** (δ = 0.23 mm·s^–1^),^22,55^ underlining the weaker σ-donating properties of pyridine and carbazolide compare to NHCs as well as the longer Fe–N^py^ bond. An almost identical value of 0.32 mm·s^–1^ was indeed recorded for the NHC/pyridine hybrid ligand ferrous complex **B**.^30^ The rather oblate electron density distribution around the metal center in **2a** is reflected in its large quadrupole splitting parameter (Δ*E*_Q_ = 2.33 mm·s^–1^, Table 3); for Fe^II^ complexes with a formally symmetric 3d^6^ low-spin configuration small quadrupole splittings are usually expected, but higher Δ*E*_Q_ values have been observed when strongly σ-donating groups are asymmetrically distributed in the coordination sphere (cf. Δ*E*_Q_ = 2.10 mm·s^–1^ for **A**).^22,90,91^ **2b** shows a significantly higher isomer shift (0.48 mm·s^–1^; Figure 12-IV and Table 3) than **2a**, compatible with the presence of intermediate spin Fe^II^.^91,92^ The very large quadrupole splitting (4.26 mm·s^–1^) reflects the large electric field gradient resulting from both the *S* = 1 ground state with (d_*xy*_)^2^(d_*yz*_)^2^(d_*xz*_)^1^(d_z^2^_)^1^(d_*x* ^2^–*y* ^2^_)^0^ configuration (cf. Figure S84) and the asymmetric square-pyramidal coordination of the metal ion.^91^

**Figure 12:**
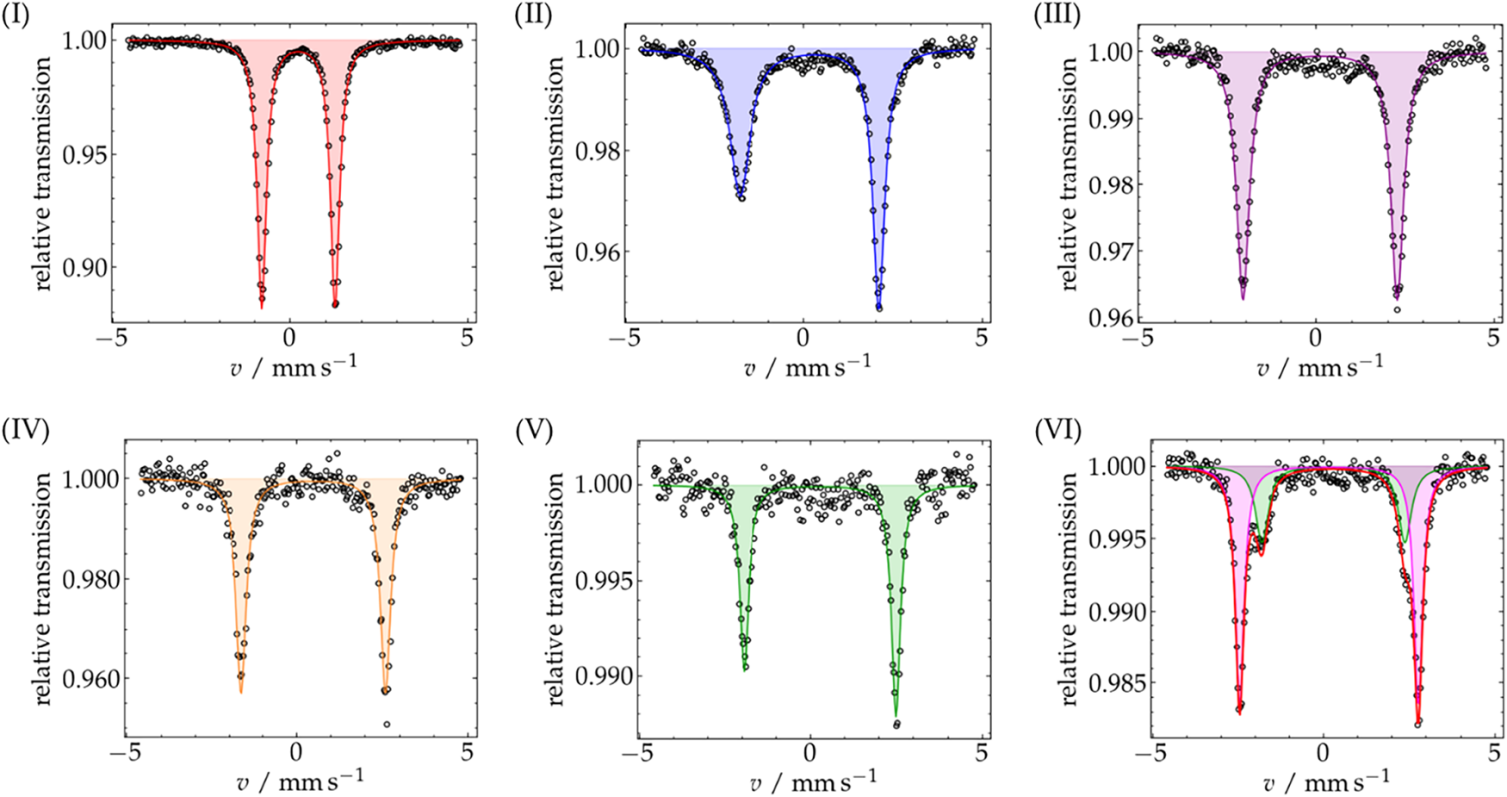
Mössbauer spectra at 80 K of solid samples of complexes **2a** (I), **3a** (II), **4a** (III), **2b** (IV), **3b** (V) and **4b** (VI).

**Table 3:** Experimental Mössbauer Parameters and DFT Calculated Values (in *italics*) of Complexes **2a/2b, 3a/3b, 4a/4b**

complex	δ	|Δ*E*_Q_|	Γ
**2a**	0.33/*0.21*	2.33/*2.24*	0.26
**2b**	0.48/*0.31*	4.26/*4.01*	0.28
**3a**	0.18/*0.15*	3.84/*3.06*	0.41
**3b**	0.29/*0.18*	4.43/*3.89*	0.32
**4a**	0.09/*0.08*	4.36/*4.00*	0.30
**4b**	0.16/*0.17*	5.23/*3.15*	0.31

DFT calculated chemical shifts were estimated according to Römelt et al.^93^ Isomer shifts δ, quadrupole splittings Δ*E*_Q_ and line widths Γ are given in mm·s^–1^. Calculated values for **4a** and **4b** are given for the lowest single point energy configuration, with a low or intermediate spin Fe^III^ center antiferromagnetically coupled with a radical on the carbazole moiety (see DFT section).

Variable temperature SQUID magnetometry additionally confirmed the *S* = 1 ground state for **2b**. Measurements performed on a powder sample at 0.5 T in the temperature range 210–2 K show a temperature independent χ_M_ *T* value of 1.24 cm^3^·K·mol^–1^ above 50 K (Figure 13) and a decrease of χ_M_ *T* below 50 K due to zero-field splitting (ZFS; VTVH measurements are shown in Figure S54). These data could be well simulated with *S* = 1, *g* = 2.24 and a single ion ZFS parameter *D* = 41.8 cm^–1^ (see SI for details).

**Figure 13:**
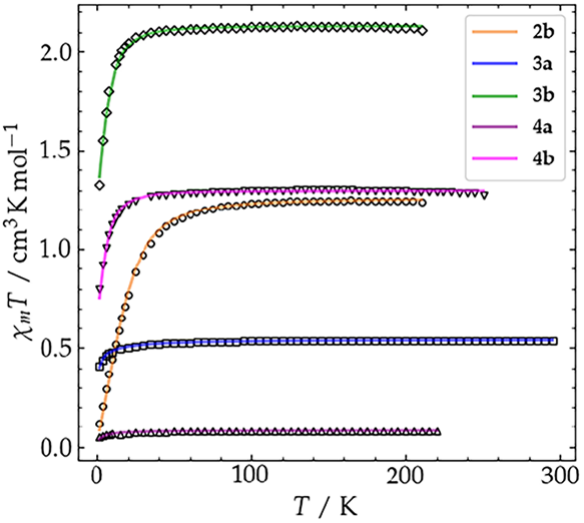
χ_M_ *T vs* *T* plots of complexes **2b** (circles and orange fit), **3a** (squares and blue fit), **3b** (diamonds and green fit), **4a** (triangles pointing up and purple fit) and **4b** (triangles pointing down and magenta fit).

Complex **3a** shows a ^57^Fe Mössbauer isomer shift of δ = 0.18 mm·s^–1^ (Table 3) compatible with a metal-based oxidation of **2a** resulting in a low-spin (*S* = 1/2) Fe^III^ complex. The large quadrupole splitting (Δ*E*_Q_ = 3.84 mm·s^–1^) suggests anisotropically populated 3d orbitals, as expected for an octahedral low-spin Fe^III^ complex.^91^ The presence of three strong-field equatorial donors such as imidazol-2-ylidenes and carbazolide combined with axial nitrile donors dictates the preference for low-spin configurations, as seen for related complexes such as the Fe^III^ congeners of **A** and **B**,^22,30,55^ and such a high Δ*E*_Q_ value likely reflects contributions from the covalency of the Fe–N^cbz^ and Fe–C bonds. The asymmetry of the spectrum (Figure 12-II) is not unusual for a half-integer spin Fe^III^ species, which often show effects of fast paramagnetic relaxation on the Mössbauer spectroscopy time scale.^91^

EPR spectroscopy on a frozen PrCN solution of **3a** reveals a rhombic spectrum, which can be simulated with *g* values of 2.13, 2.05, and 1.96 (Figure 14), supporting the presence of a metal-based radical. However, the rather low average *g* value (2.05) suggests some degree of delocalization of the spin density on the organic backbone of the complex. Indeed, the DFT calculated spin density plot for **3a** (Figure 15) indicates delocalization of the spin population on the N^cbz^ donor atom and, to a lower extent, on the entire carbazolide backbone (see the DFT calculations section for further detail). SQUID magnetometry of a solid sample of **3a** in the temperature range 295–2 K in shows an almost constant χ_M_ *T* value of 0.54 cm^3^·K·mol^–1^ (Figure 13), which is in accordance with a low spin (*S* = 1/2) Fe^III^ system with some orbital contribution (*g* = 2.23; note that the difference between microscopic *g* values derived from EPR and the effective *g* value derived from bulk susceptibility measurements may originate from, e.g., different sample conditions, viz. frozen solution vs solid material, weak magnetic interactions, etc.). The slight decrease of χ_M_ *T* below 30 K is probably due to intermolecular antiferromagnetic interactions and has been modeled with a Weiss temperature Θ = −0.33 K (see SI for details).

**Figure 14:**
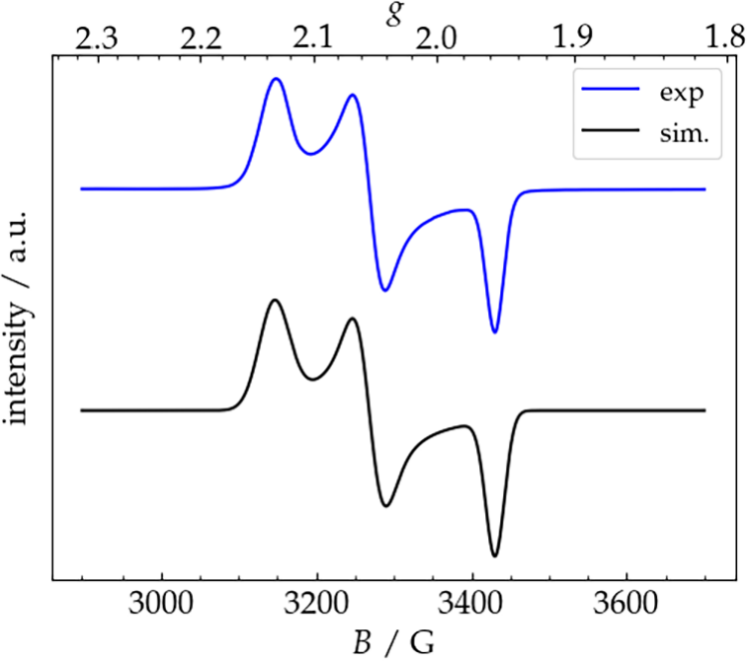
X-band EPR spectrum (9.393506 GHz, 9.875 mW, modulation: 100 kHz, 4.00 G) at 140 K of a frozen 1 mm solution of **3a** in butyronitrile.

**Figure 15:**
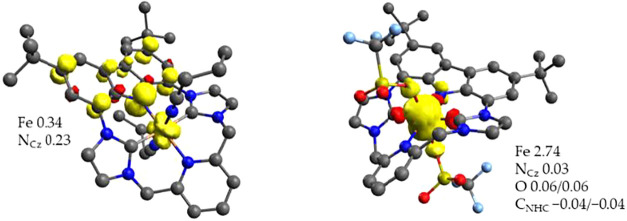
Spin density plots (isovalue 0.005) of complex **3a** (left) and **3b** (right); spin distribution according to Löwdin population analysis.

Similar to **2a**/**3a**, the decrease of the ^57^Fe Mössbauer isomer shift from 0.48 (**2b**) to 0.29 mm·s^–1^ (**3b**; Table 3 and Figure 12) supports a largely metal-centered oxidation, compatible with an intermediate spin (IS) nature of the oxidized product. Indeed, related IS Fe^III^ complexes of strong field macrocyclic ligands show similar isomer shift values.^94–97^ As seen for Fe^II^ complexes **2a** and **2b**, the presence of strong field donors in the equatorial plane prevents any high-spin (*S* = 5/2) state for the Fe^III^ complexes **3a** and **3b** while the identity of the axial ligands determines the preference for either low-spin (*S* = 1/2) or IS (*S* = 3/2) states, with the weak field triflate anions stabilizing the latter one.^53^ The increase in the quadrupole splitting (Δ*E*_Q_ = 4.43 mm·s^–1^) reflects once again both the markedly anisotropic population of the d orbitals and the covalency of the Fe–N^cbz^ bond. Indeed, the longer Fe–N^py^ bond compared to Fe–N^cbz^ together with the very long axial Fe–O bonds explain the large electric field gradient at the central metal ion. SQUID measurements on solid **3b** between 210 and 2 K confirm the *S* = 3/2 ground state, the χ_M_ *T* value of 2.11 cm^3^·K·mol^–1^ at 210 K being in line with the expected value. The decrease of χ_M_ *T* at temperatures below 30 K can be attributed to zero field splitting with best fit parameters *g* = 2.13 and *D* = 9.0 cm^–1^ (Figures 13 and S55).

As observed in variable temperature UV/vis spectra, **3b** dissolved in THF maintains its electronic structure (*S* = 3/2 ground state) down to −80 °C (Figure S19). In contrast, when dissolved in acetone, the UV/vis spectrum of **3b** at low temperatures showed incomplete conversion to a species with an absorption spectrum similar to **3a** (Figure S20). This was observed for several batches of **3b**, making binding of residual CH_3_CN in the sample an unlikely explanation. Addition of NaOTf to an acetone solution of **3b** did not change the UV/vis spectrum significantly (Figure S21), indicating that O-coordination of acetone solvent molecules is the likely cause for the formation of a low-spin species.

By addition of aliquots of CH_3_CN to a solution of **3b**, *K*_2_ for the formation of **3a** was determined in both acetone and THF at 20 °C (Figure 5). When compared to **2a** in acetone, the *K*_2_ value for **3a** is increased by one order of magnitude, whereas in THF the *K*_2_ value for **3a** is only twice that of **2a** (Table 1); this may reflect the greater ability of acetone (dielectric constant ε = 20.7) to stabilize the highly charged species with separated OTf^–^ counteranions compared to less polar THF (ε = 7.58). When the temperature is lowered by 40 °C, *K*_2_ in acetone increases by more than one order of magnitude, similar to what was found for **2a** in THF. For the formation of **3a** as well as for **2a**, intermediates with only one nitrile ligand coordinated were not observed in the UV/vis spectra (Figure S22), reflecting a strong preference for the bis­(nitrile) adduct.

Further insight into the electronic structures of complexes **3a**/**3b** was obtained with density functional theory (DFT) calculations, using the ORCA program suite and BP86 and B3LYP functionals.^98,99^ Interestingly, a plot of the spin density for complexes **3a** and **3b** reveals a large degree of spin delocalization on the carbazole backbone for the low-spin complex **3a**, indicating significant covalency of the Fe–N^cbz^ π bond (Figures 15 and S89). Conversely, the three unpaired electrons of **3b** are mainly located on the metal ion (Figures 15 and S95). TD-DFT calculations reflect this finding, assigning the broad low energy band in the UV/vis spectra of **3a** in the NIR region (calculated at 897 nm) to a transition between orbitals delocalized along the Fe–N^cbz^ π bond (Figure S93) while the absorption band of **3b** at 713 nm (calcd. 651 nm) is assigned to an LMCT transition from the carbazole backbone (Figure S99).

### Electronic Structure of **4a** and **4b**: A Ligand-Based Oxidation

Upon bulk oxidation of **3a** to **4a** the ^57^Fe Mössbauer isomer shift decreases slightly from 0.18 mm·s^–1^ (**3a**) to 0.09 mm·s^–1^ (**4a**; Table 3 and Figure 12-III), which indicates that de-electronation may occur at the carbazole fragment of the macrocyclic ligand.^36^ SQUID magnetometry of a powdered sample of **4a** shows χ_M_ *T* values close to zero, indicating a diamagnetic (*S* = 0) ground state in the entire temperature range (2–220 K to avoid decomposition at higher temperatures; Figure 13). This is in accordance with ligand-based oxidation giving a strongly antiferromagnetically coupled low-spin Fe^III^/π-radical system, rather than an *S* = 1 Fe^IV^ complex. The temperature independence of the SQUID data shows that the coupling must be very strong (|2*J*| ≥ 1000 cm^–1^).^100^ Indeed, a butyronitrile solution of **4a** proved to be EPR silent, and ^1^H NMR spectroscopy at −35 °C of a CD_3_CN solution of **4a** confirmed its diamagnetic behavior in solution (Figure S45). Together with the optical absorption features observed by UV-SEC and UV/vis spectroscopy, these experimental findings collectively support the description of **4a** as having a low-spin Fe^III^ ion antiferromagnetically coupled with a carbazole-based radical.^83,84^

Similarly, the one-electron oxidation of the *S* = 3/2 complex **3b** to give complex **4b** leads to a slight decrease of the ^57^Fe Mössbauer isomer shift (from 0.29 to 0.16 mm·s^–1^) and a marked increase in the quadrupole splitting (from 4.43 to 5.23 mm·s^–1^, Table 3 and Figure 12-VI). SQUID measurements performed on solid **4b** support the presence of an intermediate spin Fe^III^ ion (*g*_Fe_ = 2.22, *D*_Fe_ = −9.6 cm^–1^) very strongly (|2*J*| ≥ 1200 cm^–1^) antiferromagnetically coupled to the radical on the ligand (*g*_radical_ = 2.00, fixed) with resulting total spin ground state *S* = 1 (Figures 13 and S56), thus indicating a situation analogous to the one in **4a**, in agreement with the UV/vis data.

The UV/vis spectrum of **4b** in acetone shows no significant variations when lowering the temperature to −80 °C, indicating that the electronic structure remains unchanged (Figure S26). UV/vis spectra of **4b** in acetone showed the presence of **3b** as an impurity, as was also observed in the Mössbauer spectrum of solid material (cf. Figure 12-VI). When dissolving **4a** in acetone, the UV/vis spectrum of clean **4b** was obtained, indicating nitrile dissociation and confirming a nitrile binding equilibrium for **4a**/**4b** in solution (Figure S27). Consequently, addition of CH_3_CN to an acetone solution of **4b** led to formation of **4a** with its characteristic absorption band (Figure S28). Because of the presence of **3a/3b** as an impurity, *K*_2_ of **4b** was not determined.

To support the experimental evidence for a ligand-based oxidation, DFT calculations on complexes **4a** and **4b** have been run considering different possibilities for their electronic structure. In the case of **4a**, we compared the results obtained for a Fe^IV^ singlet state, a broken symmetry state with two *S* = 1/2 spin centers as well as a triplet state. The broken symmetry state is the lowest in energy (1.4 kcal·mol^–1^ lower than the singlet of a genuine Fe^IV^ complex at the B3LYP level) and reproduced best the experimental spectroscopic features (Figure S103). However, when the fraction of Hartree–Fock exchange in the calculation is reduced, the small energy difference between the singlet and BS­(1,1) states vanishes (Table S8). Hence, some genuine low-spin Fe^IV^ character cannot be excluded based on the DFT calculations. It should be noted though that the observed Mössbauer isomer shift of 0.09 mm·s^–1^ for **4a** is higher than for typical six-coordinate heteroleptic Fe^IV^ species with several NHC donors,^22,92,101^ but is in good agreement with the calculated value for the BS­(1,1) state (Table 3). A plot of the magnetic orbitals of complex **4a** calculated at the B3LYP level shows one largely metal based singly occupied orbital and the other extensively delocalized on the carbazole fragment (Figure 16, left). This supports the description of **4a** as a low-spin Fe^III^ system antiferromagnetically coupled to a π-cation radical that is mostly located on the carbazole moiety (though with significant Fe contribution). According to TD-DFT, the calculated electronic transitions at 965, 674, and 662 nm (the latter with lower calculated intensity; the former experimentally observed at 804) would be associated with ligand-based processes involving the p orbitals of both the carbazolide and NHC moieties, whereas the band calculated at 789 nm (experimental 728 nm) is associated with a transition between orbitals delocalized on the Fe–N^cbz^ π bond (Figure S105).

**Figure 16:**
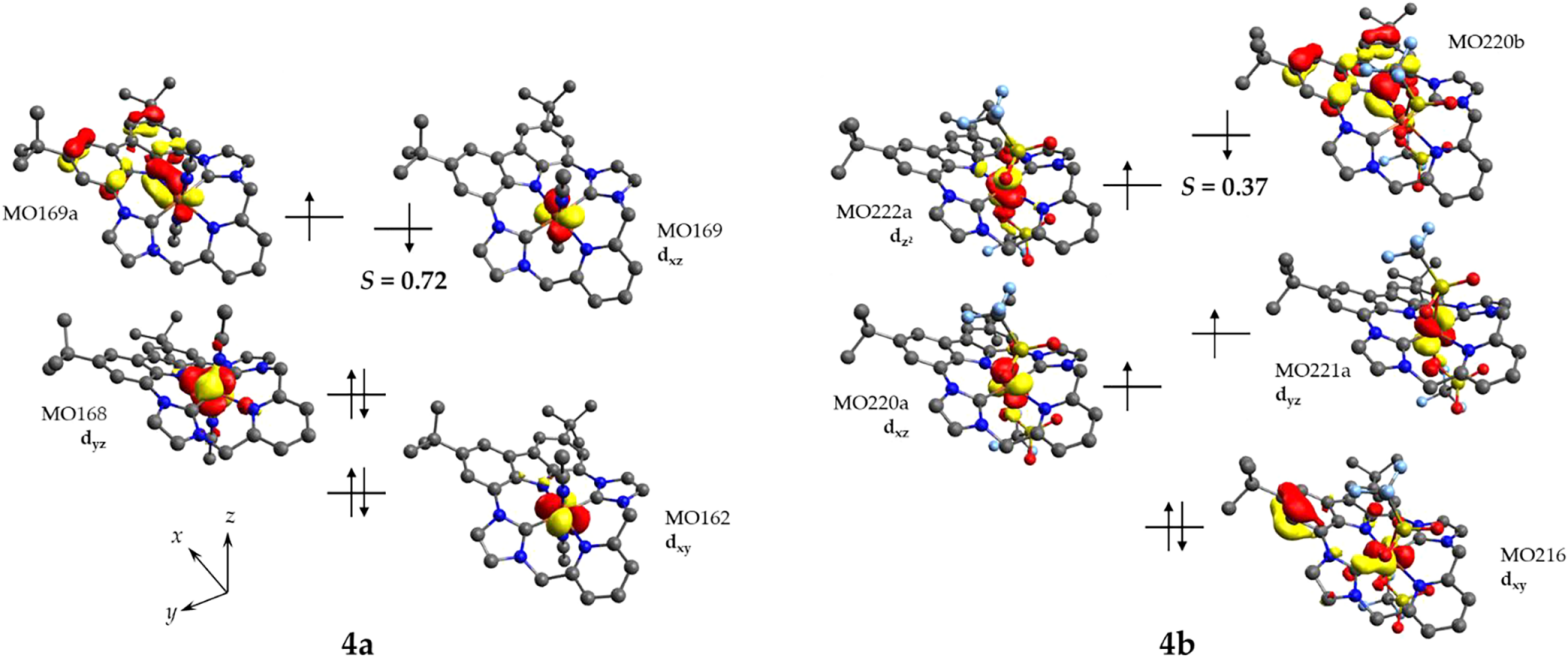
Schematic molecular orbital diagram of **4a** (left) and **4b** (right), represented by unrestricted corresponding orbitals (isovalue 0.05). Orbitals with significant overlap are depicted as doubly occupied, *S* denotes the overlap integral between the spin-down and the corresponding spin-up molecular orbital.

An analogous situation is found for **4b**. The broken symmetry state involving an intermediate-spin Fe^III^ that is antiferromagnetically coupled with a carbazolide-based radical (total spin *S* = 1) shows a single point energy 3.9 kcal·mol^–1^ lower than an *S* = 1 Fe^IV^ state at the B3LYP level. As in the case of **4a**, reducing the fraction of Hartree–Fock exchange in the calculation results in a smaller energy difference (Table S10). The four magnetic orbitals calculated at the B3LYP level (Figure 16, right) show three singly occupied spin-up orbitals located on the metal center and one spin-down orbital on the carbazolide moiety. Overall, DFT calculations support the proposed ligand-based oxidation from **3a**/**3b** to **4a**/**4b** in accordance with experimental findings, although the covalency of the Fe–N^cbz^ π bond in the nitrile-ligated low-spin complexes renders an unambiguous assignment of oxidation states challenging.

## Conclusions

This work presents the synthesis of a novel NHC/N-donor hybrid macrocyclic proligand ([LH_3_]­(OTf)_2_) that (after deprotonation) contains two *trans* NHC moieties, a pyridine and a redox active carbazolide fragment, and that provides a strong field square planar {NCNC} coordination sphere. Direct metalation of the proligand with {Fe­(hmds)_2_}_2_ in acetonitrile affords the low-spin (*S* = 0) octahedral Fe^II^ complex **2a** with two CH_3_CN molecules occupying the axial positions. When **2a** is dissolved in weakly coordinating solvents, nitrile dissociation occurs and an intermediate-spin (*S* = 1) square pyramidal Fe^II^ complex **2b** is formed, which in solid state has the triflate counteranion weakly bound in an axial position. Both **2a** and **2b** show rich redox chemistry and can be oxidized twice to sequentially give Fe^III^ (**3a** and **3b**) and formal Fe^IV^ complexes (**4a** and **4b**). **3a** and **4a** maintain the six-coordinate structure with two *trans*-axial CH_3_CN ligands and low spin ground states (*S* = 1/2 and *S* = 0, respectively), while **3b** (and possibly also **4b**) has two weakly bound triflates in axial positions and hence retains intermediate spin character (*S* = 3/2 and *S* = 1, respectively). In all cases the strong field character of the tetradentate NHC/N-donor hybrid macrocyclic platform lifts the d_*x* ^2^–*y* ^2^_ orbital high in energy, making high-spin iron complexes inaccessible. UV/vis and ^57^Fe Mössbauer spectroscopies in combination with SQUID magnetometry and DFT calculations reveal that the second oxidations (forming **4a** and **4b**) are mostly ligand-based and give carbazolide-based organic π-radicals that are antiferromagnetically coupled to the low spin or intermediate spin Fe^III^ ions, respectively.

In conclusion, two redox series of Fe complexes **2a**/**3a**/**4a** and **2b**/**3b**/**4b** of a tetradentate NHC/N-donor hybrid macrocycle are presented and comprehensively characterized. The new ligand platform combines the strong-field character of NHC-type ligands such as in **A** and **B** with ligand-based redox noninnocence typical for iron porphyrins (hemes), and hence it represents a further elaboration of the growing series of organometallic heme analogues that are based on NHC-containing tetradentate macrocycles. Such features and the possibility to tune iron spin states via the axial ligands now offer new perspectives for the (electro)­chemical activation of small molecules or the generation of bioinspired reactive oxidoiron intermediates with these heme-inspired systems. Work in that direction is ongoing in our laboratories.

## Experimental Section

### General Considerations and Materials

All syntheses and manipulations of air- and moisture-sensitive materials were carried out using standard Schlenk techniques under dry dinitrogen or argon, or in a glovebox (MBraun Unilab sp Plus ECO, O_2_ and H_2_O concentration lower than 0.1 ppm). Solvents were dried by standard procedures, degassed with dry argon, and freshly distilled before use. Deuterated solvents were degassed and dried over 3 Å molecular sieves prior to use. Starting materials were purchased from commercial sources and used as received. Compounds bimca (**1**),^39^ iron bis­(trimethylsilyl)­amide ({Fe­[N­(SiMe_3_)_2_]_2_}_2_),^57^ and thianthrene radical cation hexafluorophosphate^102^ were synthesized according to literature. Electrochemical-grade tetrakis­(*n*-butyl)­ammonium hexafluorophosphate ((^*n*^Bu_4_N)­PF_6_) was purchased from Sigma-Aldrich and dried under vacuum at 125 °C prior to use.

### Safety Statement

Caution! {Fe­[N­(SiMe_3_)_2_]_2_}_2_ is pyrophoric, corrosive and water-sensitive, it must be handled under strict exclusion of air and moisture!

### Mass Spectrometry

ESI-MS spectra were recorded on a Bruker Daltonics MicrOTOF spectrometer. ESI mass spectra are shown in Section 1 of the Supporting Information.

### UV/Vis Spectroscopy

UV/vis spectra were recorded on solutions in a quartz cuvette on a Cary 8454 instrument from Agilent Technologies or a Lambda 465 spectrometer from PerkinElmer. Temperature control was achieved using a Unisoku Cryostat (CoolSpeK) equipped with a magnetic stirrer. UV/vis titration experiments were performed by stepwise addition of acetonitrile to the solution of a compound. The obtained data were analyzed with the Musketeer software, which was used to determine equilibrium constants *K*_2_ by fitting a 1:2 binding isotherm to the experimental spectra.^103^ UV/vis spectra are shown in Section 2 of the Supporting Information.

### NMR Spectroscopy

NMR experiments were recorded on a Bruker Avance III HD 500, a Bruker Avance III HD 400, or a Bruker Avance III 300 at 500, 400 and 300 MHz, respectively. ^1^H- and ^13^C­{^1^H}-NMR chemical shifts in ppm are reported against tetramethylsilane and were referenced using the residual proton signal or the resonance of natural abundant ^13^C atoms of deuterated solvents, respectively.^104^ NMR spectra are shown in Section 3 of the Supporting Information.

### IR Spectroscopy

IR spectra were recorded on an Agilent Technologies Cary 630 FTIR spectrometer inside an argon-filled MBraun glovebox (O_2_ and H_2_O concentration lower than 0.1 ppm). IR spectra are shown in Section 4 of the Supporting Information.

### Mössbauer Spectroscopy

^57^Fe Mössbauer spectra were recorded with a ^57^Co source in a Rh matrix using an alternating constant acceleration Wissel Mössbauer spectrometer operated in the transmission mode and equipped with a Janis closed-cycle helium cryostat. Isomer shifts are reported relative to ambient temperature iron metal. The MFit program was used to simulate experimental data, using Lorentzian line doublets.^105^ Mössbauer parameters are compiled in Table 3, additional spectra are shown in Section 5 of the Supporting Information.

### EPR Spectroscopy

EPR spectra (in the X-band region) were recorded on a Bruker E500 ELEXSYS spectrometer, using a standard ER4102ST 9.45 GHz cavity, and spectra were simulated using the EasySpin program.^106,107^

### Elemental Analyses

Elemental analyses were performed by the analytical laboratory of the Institute of Inorganic Chemistry of the Georg-August University of Göttingen, using an Elementar Vario EL III instrument.

### Magnetic Measurements

Magnetic susceptibility studies were performed using a Quantum-Design MPMS XL-5 or MPMS3 SQUID magnetometer, equipped with a 5 or 7 T magnet, at 0.5 T magnetic field. The powdered samples were contained in a Teflon/gelatin/polycarbonate bucket and fixed in a nonmagnetic sample holder. Each raw data file for the measured magnetic moment was corrected for the diamagnetic contribution of the bucket according to

$$M^{\mathrm{dia}}\left(\mathrm{bucket}\right)=\chi_{g}\cdot m\cdot H$$

with an experimentally obtained gram susceptibility χ_g_ of the bucket. The molar susceptibility data were corrected for the diamagnetic contribution.

Experimental data for **2b**, **3a** and **3b** were modeled by using a fitting procedure to the spin Hamiltonian for Zeeman splitting and in case of **2b** and **3b** additionally zero-field splitting

$$Ĥ={g\mu }_{B}\mathrm{B⃗}\mathrm{S⃗}+D\left[{Ŝ}_{Z}^{2}-\frac{1}{3}S\left(S+1\right)\right]$$

Experimental data for **4a** and **4b** were modeled by using a fitting procedure to the appropriate Heisenberg–Dirac–van Vleck (HDvV) spin Hamiltonian for isotropic exchange coupling with Zeeman and zero-field splitting terms

$$Ĥ=-2J{Ŝ}_{1}{Ŝ}_{2}+g\mu_{B}\mathrm{B⃗}\left({\mathrm{S⃗}}_{1}+{\mathrm{S⃗}}_{2}\right)+D_{1}\left[{Ŝ}_{1z}^{2}-\frac{1}{3}S_{1}\left(S_{1}+1\right)\right]$$

Temperature-independent paramagnetism (TIP: 870·10^–6^ cm^3^·mol^–1^ for **2b**, 620·10^–6^ cm^3^·mol^–1^ for **3a**, 560·10^–6^ cm^3^·mol^–1^ for **3b**, 600·10^–6^ cm^3^·mol^–1^ for **4a** and 4360·10^–6^ cm^3^·mol^–1^ for **4b**) and Curie-behaved paramagnetic impurity (PI: 2.0% for **3a** and 1.8% for **4a**) with spin *S* = 5/2 were included according to

$$\chi_{\mathrm{calc}}=\left(1-\mathrm{PI}\right)\cdot \chi +\mathrm{PI}\cdot \chi_{\mathrm{mono}}+\mathrm{TIP}$$

Intermolecular interactions for **3a** were considered in a mean field approach by using a Weiss temperature (Θ = −0.33 K).^108^ The Weiss temperature *Θ (*defined as Θ = *zJ*_inter_ *S*(*S* + 1)/3*k*) relates to intermolecular interactions *zJ*_inter_, where *J*_inter_ is the interaction parameter between two nearest neighbor magnetic centers, *k* is the Boltzmann constant (0.695 cm^–1^·K^–1^) and *z* is the number of nearest neighbors. Experimental data were modeled with the julX or julX_2S programs.^109^ Variable temperature – variable field (VTVH) magnetization data are shown in Section 6 of the Supporting Information.

### Electrochemistry

Cyclic voltammetry measurements were performed in a nitrogen-filled MBraun glovebox (O_2_ and H_2_O concentration lower than 0.1 ppm), using a Gamry Interface 1000B potentiostat, and the Gamry Framework software package. All experiments were performed on solutions of crystalline material using 0.1 m (^*n*^Bu_4_N)­PF_6_ in dry solvent as electrolyte, an ALS Glassy Carbon disc electrode (1.6 mm diameter) as working electrode, a platinum wire as counter electrode and a silver wire enclosed in a glass sample holder (equipped with a Vycor glass frit) containing electrolyte solution as *pseudo*-reference electrode.^110^ All potentials were referred to the Fc^+/0^ couple (Fc = ferrocene), adding ferrocene for referencing at the end of each experiment. Compensation for *iR* drop was applied postacquisition via the Gamry EchemAnalyst software. Results of electrochemical measurements are shown in Section 7 of the Supporting Information.

### UV/Vis Spectroelectrochemistry

UV/vis spectroelectrochemical experiments were performed in a nitrogen-filled MBraun glovebox (O_2_ and H_2_O concentration lower than 0.1 ppm), using a Gamry Interface 1000B potentiostat, a BWTek deuterium/tungsten light source, a BWTek Exemplar LS spectrometer, UV/vis grade optic fibers (BWTek) and a thin layer quartz cuvette (1 mm path length). 0.1 m (*^n^* Bu_4_N)­PF_6_ in dry solvent was used as electrolyte, a platinum mesh as working electrode, a platinum wire as counter electrode and a silver wire enclosed in a sample holder (equipped with a Vycor glass frit) containing electrolyte solution as *pseudo*-reference electrode. Spectral data were analyzed using the BWSpec software. Results of UV/vis spectroelectrochemical measurements are shown in Section 8 of the Supporting Information.

### Synthetic Procedures

#### [LH_3_]­(OTf)_2_

3,6-di-*tert*-butyl-1,8-bis­(imidazol-1-yl)­carbazole (**1**; bimca) (2.00 g, 4.86 mmol, 1 equiv) and 1,6-bis­(bromomethyl)­pyridine (1.28 g, 4.86 mmol, 1 equiv) were mixed in a Schlenk flask equipped with reflux condenser, oil bubbler and magnetic stirring bar. The apparatus was degassed and 250 mL of dry and degassed CH_3_CN were added, giving a milky white suspension. The mixture was heated to reflux and stirred for 48 h, during which a white precipitate appeared. The mixture was then let to cool down to r.t., opened to air and the solvent was removed with a rotary evaporator. The yellowish residue of [LH_3_]­Br_2_ was washed multiple times with DCM and dried under vacuum to obtain 1.65 g of a yellowish solid (50% yield). Due to the low solubility of the product in most organic media, a subsequent counteranion exchange was performed without further purification: the solid was suspended in methanol and treated with AgOTf (1.32 g, 2.1 equiv), stirring the mixture for 2 h. After removal of AgBr by filtration, the solvent was evaporated, the off-white residue dissolved in CH_3_CN, filtered, and recrystallized with Et_2_O multiples times (yield: 1.55 g, 78%). Single crystals suitable for X-ray diffraction were obtained by slow diffusion of Et_2_O into a CH_3_CN solution of [LH_3_]­(OTf)_2_ at r.t. IR (ATR, solid): ν̃ [cm^–1^] = 3117 (w), 3049 (w), 2958 (w), 2908 (w), 2867 (w), 1597 (w), 1576 (w), 1553 (w), 1527 (w), 1494 (w), 1477 (w), 1455 (w), 1433 (w), 1363 (w), 1253 (s), 1223 (s), 1157 (s), 1137 (s), 1026 (s), 996 (m), 937 (w), 870 (m), 842 (w), 793 (w), 770 (w), 758 (m), 746 (w), 738 (w), 713 (w), 691 (w), 669 (m), 636 (s), 571 (s), 540 (m), 514 (s) cm^–1^. ^1^H NMR (500 MHz, DMSO-*d*_6_): δ [ppm] = 11.29 (H16, s, 1H), 10.01 (H6, t, 2H, *J* = 1.6 Hz), 8.57 (H12, d, 2H, *J* = 1.6 Hz), 8.29 (H7, H5, m, 4H), 8.09 (H1, t, 1H, *J* = 7.7 Hz), 7.87 (H2, d, 2H, *J* = 7.7 Hz), 7.82 (H10, d, 2H, *J* = 1.6 Hz), 5.59 (H4, s, 4H), 1.47 (H15, s, 18H). ^13^C­{^1^H}-NMR (126 MHz, DMSO-*d*_6_): δ [ppm] = 153.7 (C3), 144.1 (C11), 139.2 (C1), 137.1 (C6), 133.3 (C9), 125.5 (C13), 124.9 (C2), 123.0 (C5), 122.8 (C7), 119.6 (C8), 119.6 (C12), 119.3 (C10), 54.5 (C4), 35.0 (C14), 31.7 (C15). ESI­(+)-MS (CH_3_CN, *m*/*z*): 665 ([LH_3_(OTf)]^+^), 516 ([LH_3_]^+^), 258 ([LH_3_]^2+^). UV/vis (CH_3_CN): λ_max_ [nm] (ε [L·mol^–1^·cm^–1^]) = 226 (19780), 298 (7580), 337 (1870), 349 (1950).

#### [LFe­(CH_3_CN)_2_]­(OTf) (**2a**)

300.0 mg of [LH_3_]­(OTf)_2_ (0.368 mmol, 1 equiv) were dissolved in dry and degassed CH_3_CN in a nitrogen-filled glovebox. Solid {Fe­[N­(SiMe_3_)_2_]_2_}_2_ ({Fe­(hmds)_2_}_2_, 0.277 g, 0.368 mmol, 1 equiv) was added, and the mixture was stirred at r.t. for 48 h. An initial yellow precipitate formed, which dissolved overtime to give an orange-brown solution. The mixture was filtered, and the solvent removed under reduced pressure. The residue was dissolved in DCM, filtered, then the solvent was removed under reduced pressure. The orange residue was finally dissolved in 1,2-difluorobenzene (*o*-DFB), the solution filtered, the solvent was removed under reduced pressure and the orange powder dried. Dissolution in CH_3_CN and reprecipitation with Et_2_O afforded the orange-red crystalline product (yield: 214.0 mg, 73%). Single crystals suitable for X-ray diffraction were grown by slow diffusion of Et_2_O into a concentrated solution of the product in CH_3_CN inside a glovebox. IR (ATR, solid): ν̃ [cm^–1^] = 3162 (w), 3135 (w), 3102 (w), 2953 (w), 2902 (w), 2865 (w), 1583 (w), 1480 (w), 1448 (m), 1419 (m), 1363 (w), 1321 (w), 1304 (m), 1258 (s), 1223 (s), 1158 (m), 1148 (m), 1106 (m), 1072 (w), 1026 (s), 993 (w), 888 (w), 861 (w), 842 (m), 817 (w), 774 (w), 752 (m), 740 (w), 719 (w), 709 (w), 687 (w), 674 (w), 665 (w), 636 (s), 614. (m), 572 (m), 541 (w), 516 (m) cm^–1^. ^1^H NMR (500 MHz, CD_3_CN): δ [ppm] = 8.69 (H7, d, 2H, *J* = 2.1 Hz), 8.00 (H12, d, 2H, *J* = 1.6 Hz), 7.94 (H5, d, 2H, *J* = 2.1 Hz), 7.89 (H10, d, 2H, *J* = 1.5 Hz), 7.76 (H1, t, 1H, *J* = 7.7 Hz), 7.48 (H2, d, 2H, *J* = 7.7 Hz), 5.63 (H4, s, 4H), 1.54 (H15, s, 18H). ^13^C­{^1^H}-NMR (126 MHz, CD_3_CN): δ [ppm] = 202.2 (C6), 162.8 (C3), 138.8 (C11), 138.4 (C1), 137.5 (C9), 129.4 (C8), 126.0 (C13), 125.0 (C2), 124.5 (C5), 119.6 (C7), 114.6 (C12), 108.9 (C10), 54.6 (C4), 35.4 (C14), 32.6 (C15). UV/vis (CH_3_CN): λ_max_ [nm] (ε [L·mol^–1^·cm^–1^]) = 290 (∼28,560), 321 (10,100), 377 (4420), 480 (5320).

#### [LFe]­OTf (**2b**)

The synthesis of complex **2b** followed the same procedure as for complex **2a**, except for the last reprecipitation step, which for **2b** was performed by dissolving the orange powder in THF and precipitating the product with Et_2_O. Alternatively, the product could be obtained by dissolving **2a** in acetone, *o*-DFB, DMF or MeOH and reprecipitating it with Et_2_O, yielding a bright orange powder. Drying under vacuum afforded the pure product in yields similar to those of **2a**. Single crystals suitable for X-ray diffraction were grown by concentration of a saturated *o*-DFB solution of **2b** at r.t. inside a glovebox. Anal. Calcd for C_40_H_37_F_5_FeN_6_O_3_S (**2b** + *o*-DFB) [%]: C 57.7, H 4.48, N 10.09. Found: C 57.18, H 4.45, N 10.04. IR (ATR, solid): ν̃ [cm^–1^] = 2953 (w), 2902 (w), 2867 (w), 1593 (w), 1478 (w), 1438 (w), 1414 (w), 1406 (w), 1394 (w), 1363 (w), 1256 (m), 1233 (m), 1220 (s), 1155 (m), 1028 (s), 888 (w), 866 (w), 844 (w), 774 (w), 751 (w), 716 (w), 691 (w), 667 (w), 635 (s) cm^–1^. UV/vis (THF): λ_max_ [nm] (ε [L·mol^–1^·cm^–1^]) = 305 (13020), 353 (5790), 373 (6210), 389 (5810), 436 (4400), 483 (3150). SQUID: *S* = 1, *g* = 2.24, *D* = 41.8 cm^–1^, χ*T* = 1.24 cm^3^·K·mol^–1^ at 205 K.

#### [LFe­(PrCN)_2_]­(OTf)_2_ (**3a**)

Complex **2a** (15.0 mg, 1.87·10^–2^ mmol, 1 equiv) was dissolved in dry and degassed CH_3_CN in a nitrogen-filled glovebox. AgOTf (4.8 mg, 1.87·10^–2^ mmol, 1 equiv) was added and the color immediately changed from orange to blue while a precipitate of Ag appeared. The suspension was stirred at r.t. for 30 min, then Ag was removed by filtration. The solvent was removed under vacuum and the residue dissolved in PrCN and filtered. Precipitation with Et_2_O afforded a blue powder that was dried under vacuum (yield: 18.4 mg, 98%). X-ray diffraction quality single crystals were obtained by slow diffusion of Et_2_O into a concentrated PrCN solution of the product at r.t. inside a glovebox. Anal. Calcd for C_47_H_54_F_6_FeN_9_O_6_S_2_ (**3a** + PrCN) [%]: C 52.52, H 5.06, N 11.73. Found: C 52.24, H 5.17, N 11.80. IR (ATR, solid): ν̃ [cm^–1^] = 3149 (w), 3084 (w), 2951 (w), 2907 (w), 2868 (w), 2360 (w), 2331 (w), 2298 (w), 1618 (w), 1612 (w), 1595 (w), 1579 (w), 1551 (w), 1523 (w), 1472 (w), 1463 (w), 1437 (w), 1427 (w), 1405 (w), 1364 (w), 1327 (w), 1265 (s), 1252 (s), 1222 (s), 1556 (s), 1027 (s), 993 (m), 867 (m), 783 (w), 755 (m), 719 (w), 706 (w), 692 (w), 637 (s), 574 (m), 516 (s) cm^–1^. UV/vis (CH_3_CN), λ_max_ [nm] (ε [L·mol^–1^·cm^–1^]) = 262 (∼32570), 338 (8820), 419 (3220), 446 (2450), 602 (3120), 1005 (6500). EPR (frozen PrCN solution, 146 K): *g*_1_ = 2.132, *g*_2_ = 2.054, *g*_3_ = 1.957. SQUID: *S* = 1/2, *g* = 2.23, χ*T* = 0.54 cm^3^·K·mol^–1^ at 295 K.

#### [LFe]­(OTf)_2_ (**3b**)

Complex **2b** (15.0 mg, 2.09·10^–2^ mmol, 1 equiv) was dissolved in dry and degassed THF or acetone in a nitrogen-filled glovebox. AgOTf (5.4 mg, 2.1·10^–2^ mmol, 1 equiv) was added and the color of the solution immediately changed from orange to green while a green-gray precipitate appeared. The suspension was stirred at r.t. for 30 min, then the product was fully precipitated with Et_2_O. The residue was dissolved in acetone and filtered from solid Ag. Precipitation with Et_2_O afforded a green powder that was dried under vacuum (yield: 15.1 mg, 83%). X-ray diffraction quality single crystals were obtained by layering Et_2_O on top of a concentrated acetone solution at r.t. inside a glovebox. Anal. Calcd for C_35_H_33_F_6_FeN_6_O_6_S_2_ (**3b**) [%]: C 48.45, H 3.83, N 9.69. Found: C 48.15, H 3.84, N 9.58. IR (ATR, solid): ν̃ [cm^–1^] = 3128 (w), 2963 (w), 2871 (w), 1705 (w), 1610 (w), 1595 (w), 1493 (w), 1455 (w), 1440 (w), 1423 (w), 1408 (w), 1363 (w), 1298 (m), 1232 (m), 1211 (s), 1171 (m), 1115 (w), 1054 (w), 1014 (s), 894 (w), 876 (w), 868 (w), 855 (w), 844 (w), 774 (w), 760 (w), 747 (w), 666 (w), 632 (s), 579 (w), 513 (m) cm^–1^. UV/vis (acetone), λ_max_ [nm] (ε [L·mol^–1^·cm^–1^]) = 351 (8490), 405 (3320), 428 (4150), 703 (3140). SQUID: *S* = 3/2, *g* = 2.13, *D* = 9.0 cm^–1^, χ*T* = 2.12 cm^3^·K·mol^–1^ at 205 K.

#### [LFe­(CH_3_CN)_2_]­(OTf)_2_(PF_6_) (**4a**)

Complex **3a** (10.0 mg, 9.94·10^–3^ mmol, 1 equiv) was dissolved in dry and degassed CH_3_CN in a nitrogen-filled glovebox and the solution was cooled to −35 °C. Thianthrene radical cation hexafluorophosphate (3.6 mg, 9.96·10^–3^ mmol, 1 equiv) was added to the blue solution and the resulting purple solution was kept at −35 °C for 1 h. During the reaction a colorless precipitate of thianthrene appeared. The mixture was filtered, and the purple product was precipitated with Et_2_O. The resulting purple-gray solid was washed with THF to remove unreacted **3a**, then dissolved in CH_3_CN and precipitated again with Et_2_O to obtain a purple-gray powder (yield: 9.8 mg, 86%). Single crystals suitable for X-ray diffraction were obtained by slow diffusion of Et_2_O into an CH_3_CN solution of the product and 0.5 equiv. thianthrene radical cation hexafluorophosphate (to prevent slow decomposition of **4a** to **3a**) at −35 °C inside a glovebox. IR (ATR, solid): ν̃ [cm^–1^] = 2962 (w), 2937 (w), 2870 (w), 2328 (w), 2301 (w), 1616 (w), 1597 (w), 1579 (w), 1564 (w), 1539 (w), 1471 (w), 1461 (w), 1427 (w), 1398 (w), 1366 (w), 1360 (w), 1345 (w), 1255 (s), 1225 (m), 1198 (w), 1148 (s), 1109 (w), 1088 (m), 1075 (m), 1056 (m), 1029 (s), 1021 (m), 987 (m), 898 (w), 874 (w), 835 (s), 765 (w), 755 (w), 742 (m), 716 (w), 690 (w), 648 (m), 637 (s), 572 (m), 557 (s), 516 (m) cm^–1^. ^1^H NMR (400 MHz, CD_3_CN): δ [ppm] = 8.64 (t, H1, 1H, *J* = 7.8 Hz), 8.48 (2H), 8.36 (d, H2, 2H, *J* = 7.8 Hz), 8.18 (2H), 8.01 (2H), 7.35 (2H), 5.14 (H4, 4H), 1.42 (H15, 18H). UV/vis (CH_3_CN): λ_max_ [nm] (ε [L·mol^–1^·cm^–1^]) = 331 (11440), 377 (6500), 424 (4410), 511 (7670), 728 (11710), 804 (26670). SQUID: *S*_1_ = *S*_2_ = 1/2, *g*_1_ = *g*_2_ = 2.00 (fixed), |2*J*| ≥ 1000 cm^–1^.

#### [LFe­(OTf)_2_]­(PF_6_) (**4b**)

Complex **3b** (15.0 mg, 1.73·10^–2^ mmol, 1 equiv) was partially dissolved in dry and degassed THF in a nitrogen-filled glovebox and the suspension was cooled down to −35 °C. Thianthrene radical cation hexafluorophosphate (6.87 mg, 1.90·10^–2^ mmol, 1.1 equiv) was added to the green suspension and the resulting orange-brown suspension was kept at −35 °C for 1 h. The mixture was filtered, and the product precipitated from the solution by the addition of pentane. The resulting orange-brown solid was redissolved in acetone and precipitated again with pentane to obtain a purple powder (yield: 8.5 mg, 49%). IR (ATR, solid): ν̃ [cm^–1^] = 2962 (w), 2870 (w), 1596 (w), 1566 (w), 1541 (w), 1459 (w), 1439 (w), 1431 (w), 1362 (w), 1292 (w), 1230 (m), 1205 (m), 1153 (m), 1091 (w), 1077 (w), 1059 (w), 1021 (m), 835 (s), 760 (s), 752 (s), 716 (w), 635 (s), 557 (s) cm^–1^. UV/vis (acetone): λ_max_ [nm] (ε [L·mol^–1^·cm^–1^]) = 350 (17680), 409 (9230), 420 (9130), 512 (5910), 667 (5970), 739 (5810), 834 (9520). SQUID: *S*_1_ = 3/2, *S*_2_ = 1/2, *g*_1_ = 2.22, *g*_2_ = 2.00 (fixed), *D*_1_ = −9.6 cm^–1^, |2*J*| ≥ 1200 cm^–1^.

### X-ray Crystallography

Crystallographic details can be found in Section 9 of the Supporting Information: crystal data and details of the data collections are given in Tables S1 and S2, selected bond lengths and angles in Table S3, molecular structures are shown in Figures S70–S75. X-ray data were collected on a STOE IPDS II or a BRUKER D8-QUEST diffractometer (monochromated Mo–Kα radiation, λ = 0.71073 Å) by use of ω or ω and φ scans at low temperature. The structures were solved with SHELXT and refined on *F* ^2^ using all reflections with SHELXL.^111,112^ Non-hydrogen atoms were refined anisotropically. Hydrogen atoms were placed in calculated positions and assigned to an isotropic displacement parameter of 1.5/1.2 *U*_eq_(C/N).

One ^*t*^Bu group (occupancy factors: 0.692(4)/0.308(4)) and a CF_3_SO_3_^–^ anion (occupancy factors: 0.730(12)/0.270(12)) have been found disordered in case of [LH_3_]­(OTf)_2_. SADI restraints (*d*(C–CH_3_), *d*(C^ar^–C), *d*(C···C)) in case of the ^*t*^Bu group and SAME, RIGU restraints and EADP constraints in case of CF_3_SO_3_^–^ were applied to model the disordered parts. Two disordered Et_2_O solvent molecules (occupancy factors: 0.80(2)/0.20(2) and 0.825(3)/0.175(3)) and three F atoms of a CF_3_SO_3_^–^ anion (occupancy factors: 0.928(19)/0.072(19)) have been found disordered in **2a**. DFIX (*d*(CH_2_–CH_3_) = 1.51 Å, *d*(CH_2_–O) = 1.43 Å), SAME, SIMU, DELU, BUMP restraints and EADP constraints have been applied for the Et_2_O molecules and SADI (*d*(C–F), *d*(F···F)) restraints and EADP constraints for the CF_3_SO_3_^–^ anion. In **4a** an Et_2_O solvent molecule (occupancy factors: 0.55(2)/0.45(2)) and a CF_3_SO_3_^–^ anion (occupancy factors: 0.679(13)/0.321(13)) have been found disordered. SAME and RIGU restraints were used in both cases. A disorder of the two ^*t*^Bu groups of the ligand (occupancy factors: 0.624(5)/0.376(5) and 0.584(4)/0.416(4)), a disorder of a coordinating butyronitrile molecule (occupancy factors: 0.635(9)/0.365(9)) and a CF_3_SO_3_^–^ anion/butyronitrile lattice solvent molecule disorder (occupancy factors: 0.478(2)/0.522(2)) have been refined in case of **3a**. The following restraints and constraints have been applied to model the disordered parts: SADI (*d*(C–CH_3_), *d*(C^ar^–C), *d*(C···C)), DELU, RIGU and EADP for the ^*t*^Bu groups, SAME, DELU, RIGU and EADP for the coordinating butyronitrile molecule and SAME, DELU, RIGU and EADP for CF_3_SO_3_^–^/butyronitrile.

Face-indexed absorption corrections were performed numerically with the program X-RED^113^ or by the multiscan method with SADABS.^114^

### Computational Details

Density functional theory (DFT) computations were performed with the ORCA 5.0.4 quantum chemical package,^98,99^ using the BP86 and B3LYP functionals,^115,116^ and def2-tzvp and def2/J basis sets,^117,118^ as well as the atom-pairwise dispersion correction with Becke-Johnson damping scheme (D3BJ).^119,120^ Geometries of **2a**, **2b**, **3a** and **3b** were optimized from the corresponding crystal structures coordinates. The broken symmetry (BS) geometry optimization of complex **4a** was run starting from the crystal structure coordinates of **3a**, while optimizations of the singlet and triplet state were run starting from coordinates of **4a**. The optimization of the structure of complex **4b** was run starting from the coordinates of complex **3b**. True minima at the optimized geometries were confirmed via calculation of vibrational frequencies, which resulted in no imaginary modes. For the calculation of ^57^Fe Mössbauer parameters, the CP­(PPP) basis set was used for the iron atoms. UV/vis spectra were calculated by time-dependent DFT (TD-DFT)^121,122^ computations at the B3LYP/D3BJ def2-tzvp def2/J level of theory, employing 80 states and the CPCM continuum solvation model for solvent contributions, after previous optimization of the structure within the solvation model. Additional information is provided in Section 10 in the Supporting Information.

## Supplementary Material





## Notes

ESI mass spectra, UV/vis spectra, NMR spectra, IR spectra, Mössbauer spectra, magnetic susceptibility measurements, cyclovoltammograms and UV/vis spectroelectrochemical measurements, crystallographic and computational details (PDF)
Computationally optimized geometries of all complexes (XYZ)

## §

Department of Chemistry, Indian Institute of Technology Delhi, 110016 New Delhi, India

## Notes

Dedicated in memoriam Prof. George M. Sheldrick, a pioneer in the field of structural chemistry and X-ray diffraction methods.
