# Supplementary material for: Redox and Spin States Series of an Organometallic Heme Analogue Based on a Non-Innocent NHC/N-Donor Hybrid Macrocycle
Source: Inorg Chem. 2026 Jan 20;65(4):2184–202. doi: 10.1021/acs.inorgchem.5c04599 (PMC12869501; doi:10.1021/acs.inorgchem.5c04599)
Supplement: Supplementary file 1 [file ic5c04599_si_001.pdf]

Supporting Information

Redox and Spin States Series of an Organometallic Heme Analogue Based on a Non-Innocent NHC/N-Donor Hybrid Macrocycle

Massimiliano Morganti,[a] Jan C. Kruse,[a] Sandeep K. Gupta,[a] Sebastian Dechert,[a] Serhiy Demeshko[a] and Franc Meyer[a,b,*]

[a] University of Göttingen, Institute of Inorganic Chemistry, Tammannstraße 4, 37077 Göttingen, Germany

[b] University of Göttingen, International Center for Advanced Studies of Energy Conversion (ICASEC), Tammannstraße 6, D-37077 Göttingen, Germany

* to whom correspondence should be addressed; email: franc.meyer@chemie.uni-goettingen.de

Table of Contents

1. ESI Mass Spectrometry S2
2. UV/Vis Spectroscopy S3
3. NMR Spectroscopy S19
4. IR Spectroscopy S27
5. Mössbauer Spectroscopy S30
6. Magnetic Susceptibility Measurements S30
7. Electrochemistry S32
8. UV/Vis Spectroelectrochemistry S37
9. Crystallographic Details S39
10. DFT Calculations S46
10.1. Complex **2a** S46
10.2. Complex **2b** S49
10.3. Complex **3a** S53
10.4. Complex **3b** S56
10.5. Complex **4a** S59
10.6. Complex **4b** S64

1. ESI Mass Spectrometry

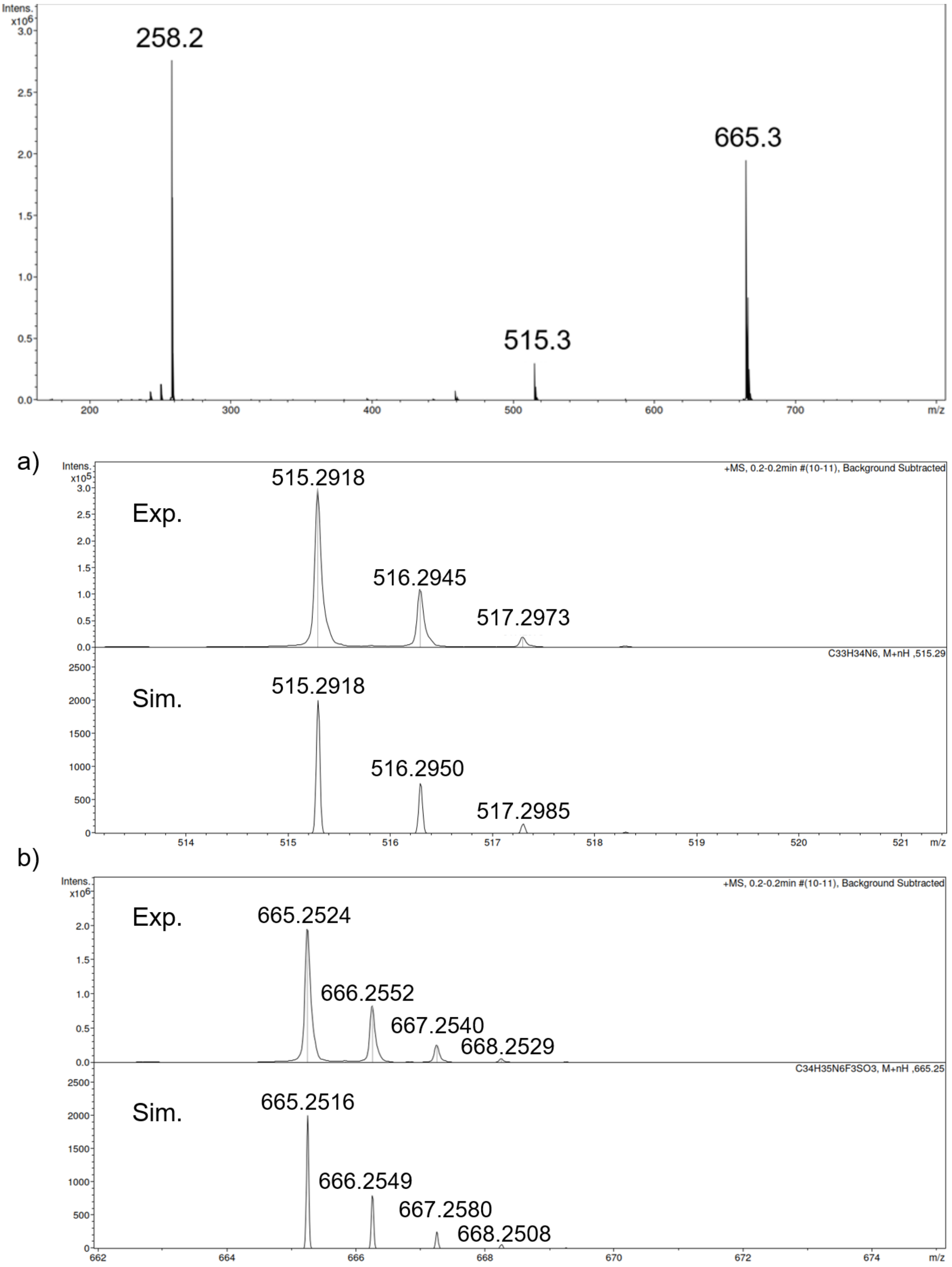

Figure S1. *ESI(+) mass spectrum of a CH_3CN solution of $[LH_3](OTf)_2$ (top) and experimental and simulated isotopic pattern of the signals at m/z 515 (middle, a) and 665 (bottom, b) corresponding to the ions $[LH_3]^+$ and $[LH_3(OTf)]^+$, respectively.*

2. UV/Vis Spectroscopy

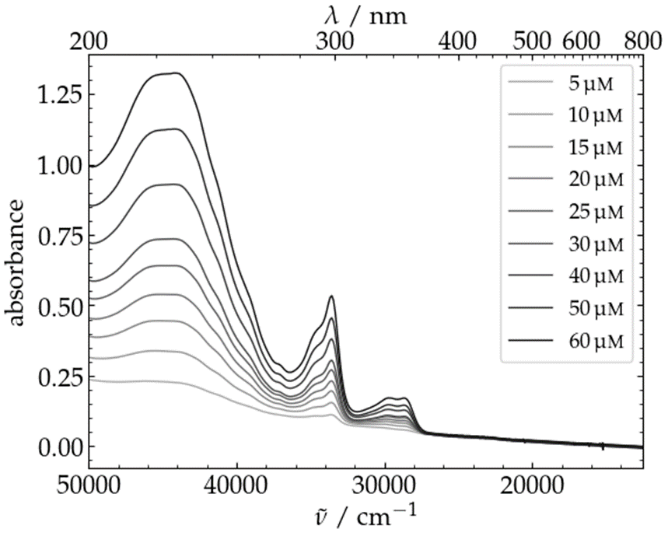

Figure S2. *UV/Vis spectra of CH_3CN solutions of $[LH_3](OTf)_2$ at different concentrations at room temperature.*

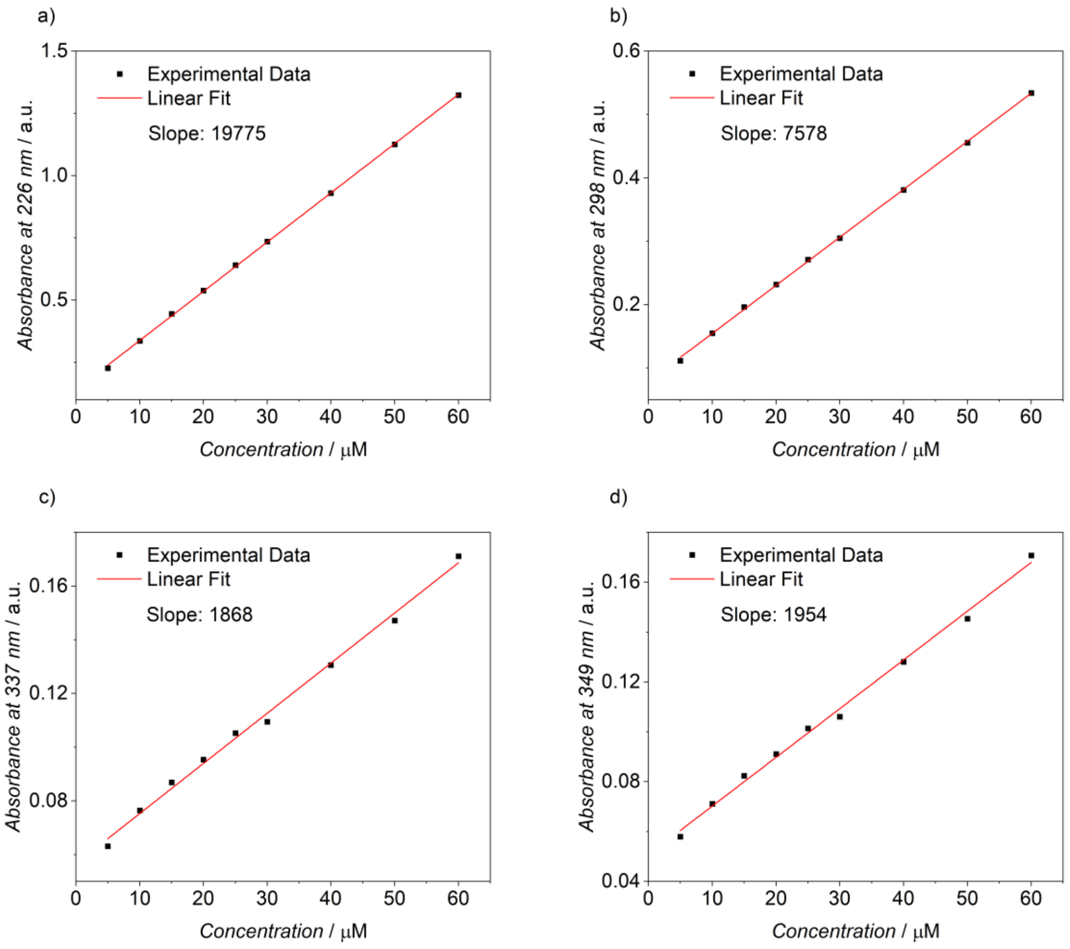

Figure S3. *Plots and linear fits of absorbance at a) 226, b) 298, c) 337, d) 349 nm versus concentrations of $[LH_3](OTf)_2$ in CH_3CN at room temperature. The slope* (in *$L{\cdot}mol^{-1}$*) of the linear fit *has been used as an estimation of the molar extinction coefficients of the compound at the given wavelengths.*

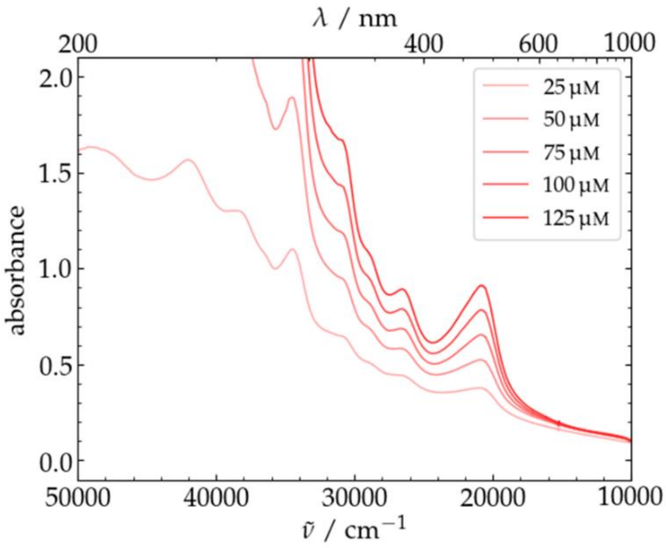

Figure S4. *UV/Vis spectra of CH_3CN solutions of* ***2a*** *at different concentrations at room temperature.*

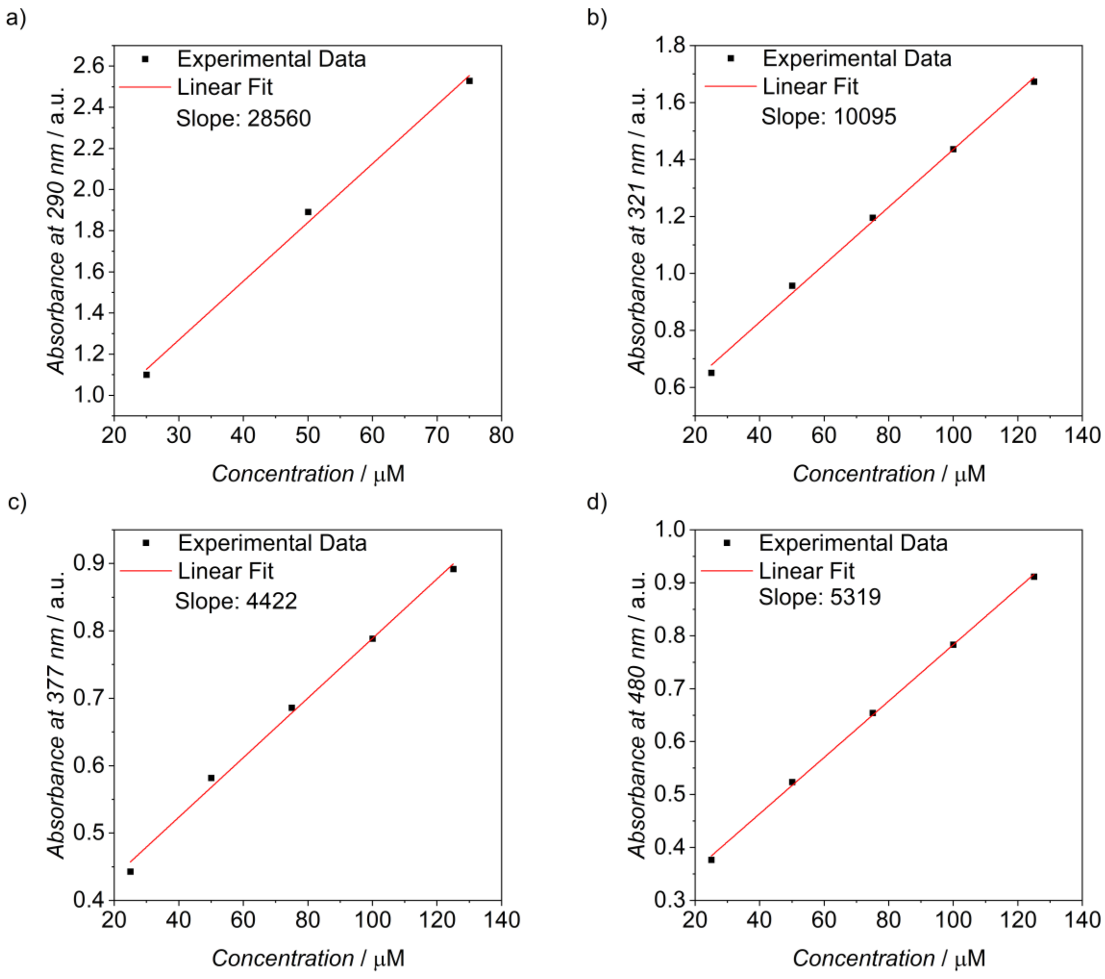

Figure S5. *Plots and linear fits of absorbance at a) 290, b) 321, c) 377, d) 480 nm versus concentrations of* ***2a*** *in CH_3CN at room temperature. The slope (in $L{\cdot}mol^{-1}$) of the linear fit has been used as an estimation of the molar extinction coefficients of the complex at the given wavelengths.*

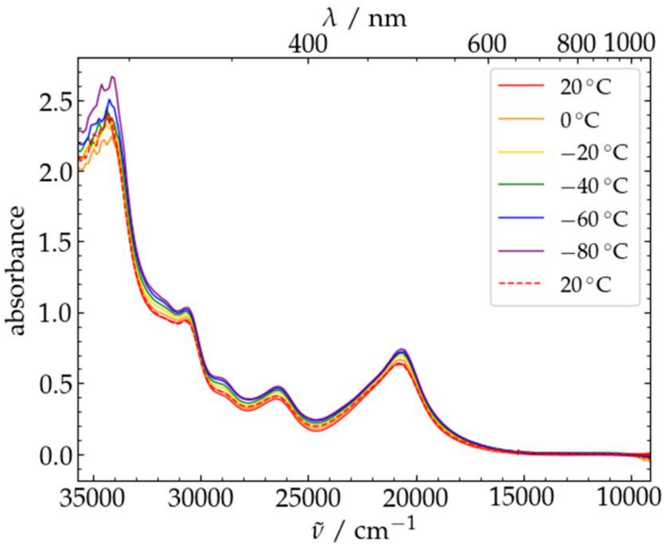

Figure S6. *UV/Vis spectra of a 0.1 mM PrCN solution of* ***2a*** *at different temperatures while cooling down as well as after heating up back to 20 °C (dashed line).*

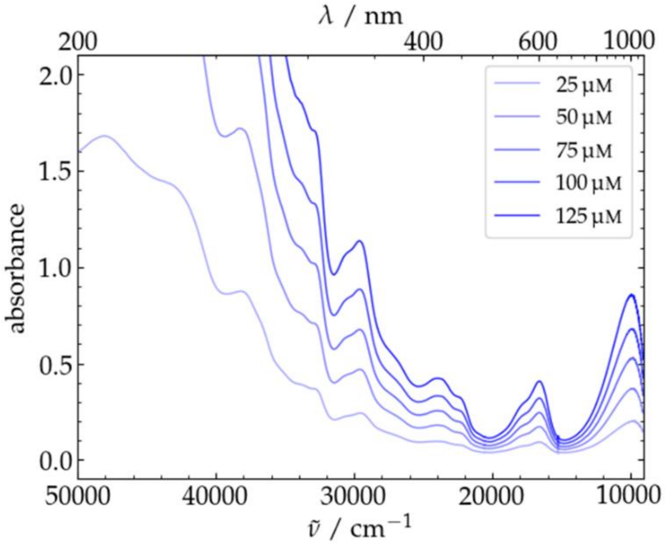

Figure S7. *UV/Vis spectra of* CH_3CN *solutions of* ***3a*** *at different concentrations at room temperature.*

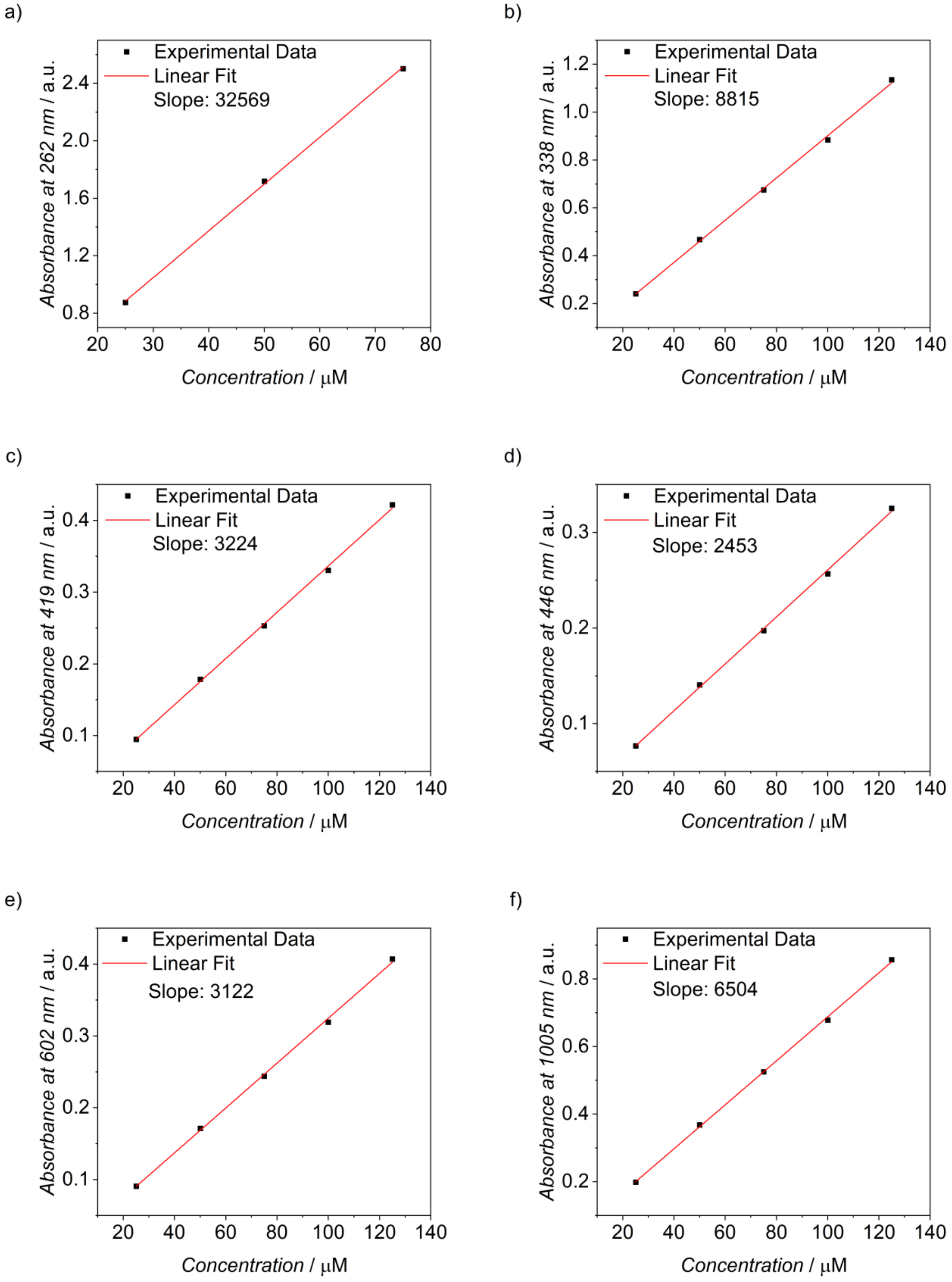

Figure S8. *Plots and linear fits of absorbance at a) 262, b) 338, c) 419, d) 446, e) 602, f) 1005 nm versus concentrations of* ***3a*** *in CH_3CN at room temperature. The slope (in $L\cdot mol^{-1}$) of the linear fit has been used as an estimation of the molar extinction coefficients of the complex at the given wavelengths.*

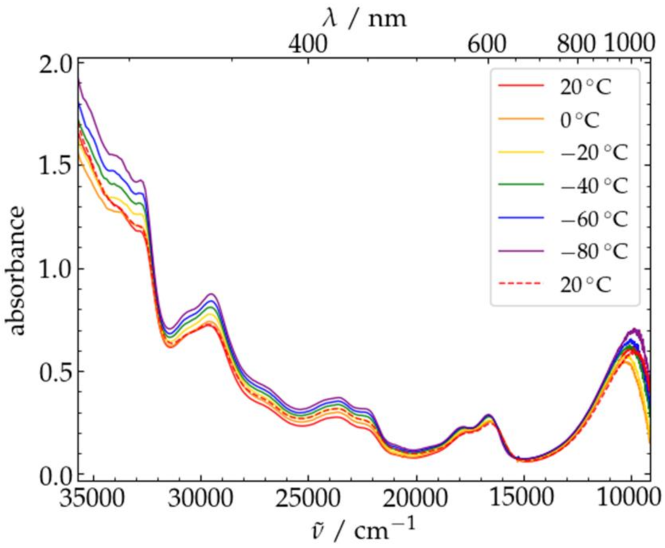

Figure S9. *UV/Vis spectra of a 0.1 mM PrCN solution of* ***3a*** *at different temperatures while cooling down as well as after heating up back to 20 °C (dashed line).*

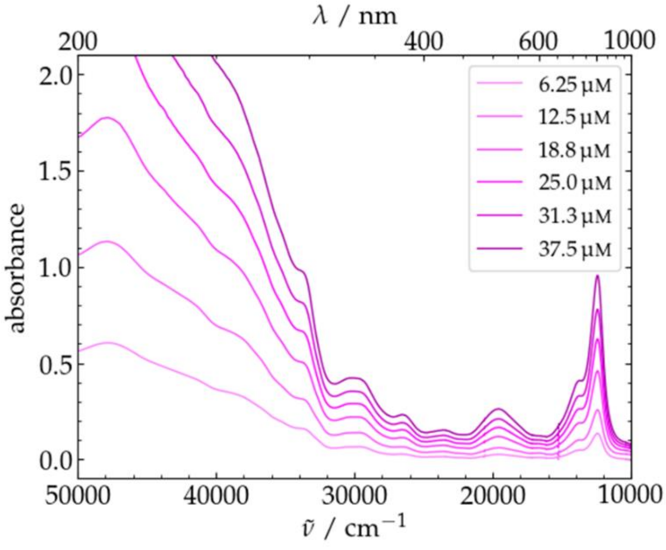

Figure S10. *UV/Vis spectra of CH_3CN solutions of* ***4a*** *at different concentrations at −40 °C.*

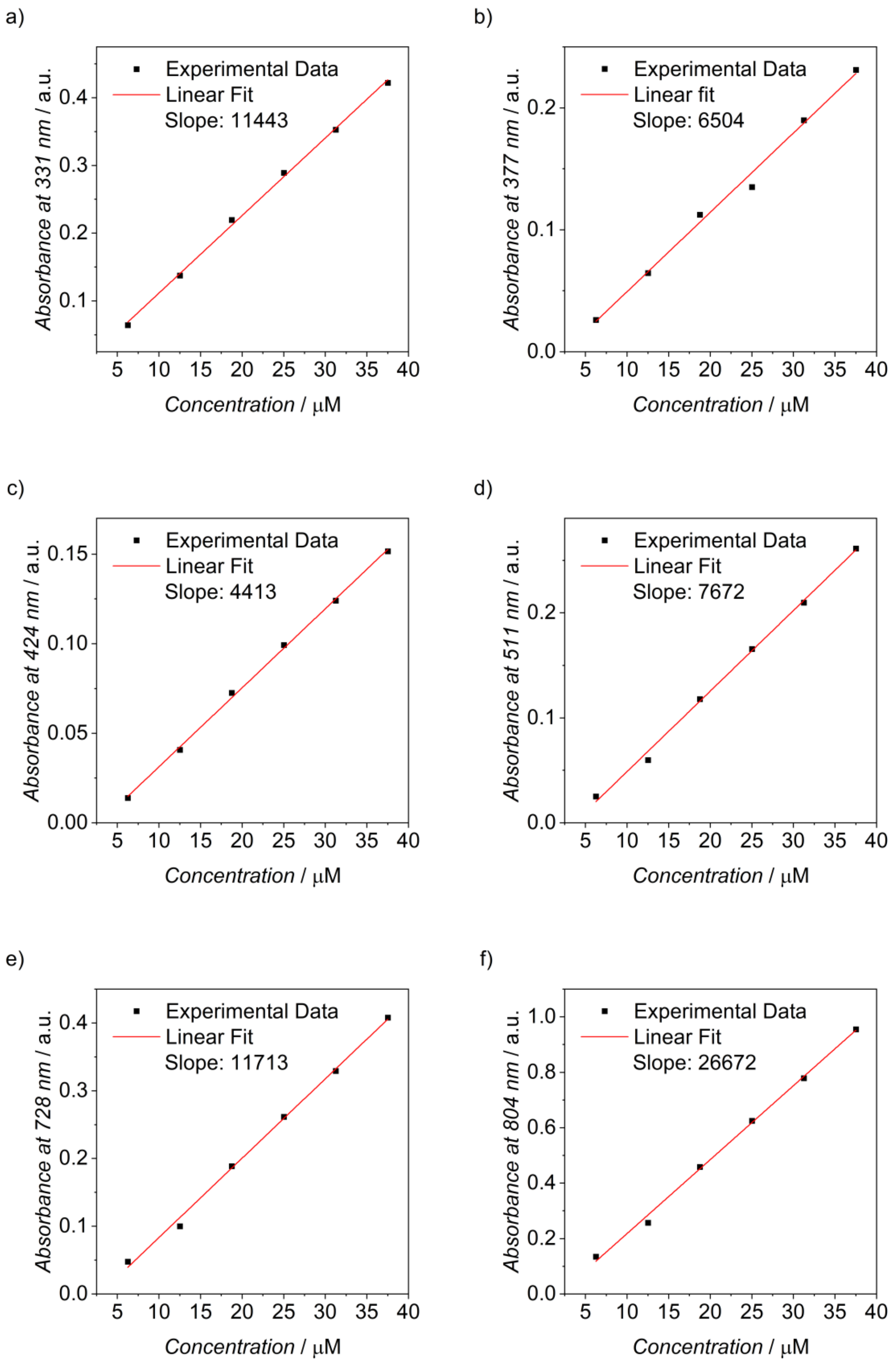

Figure S11. *Plots and linear fits of absorbance at a) 331, b) 377, c) 424, d) 511, e) 728, f) 804 nm versus concentrations of* ***4a*** *in* CH_3CN *at −40 °C. The slope (in* $L{\cdot}mol^{-1}$*) of the linear fit has been used as an estimation of the molar extinction coefficients of the complex at the given wavelengths.*

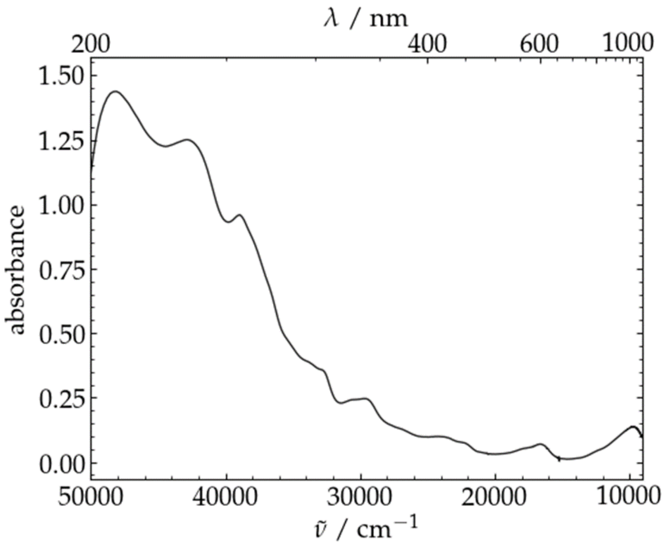

Figure S12. *UV/Vis spectrum of a CH_3CN solution of **4a** that was left at r.t. for 30 minutes, showing decomposition to **3a**.*

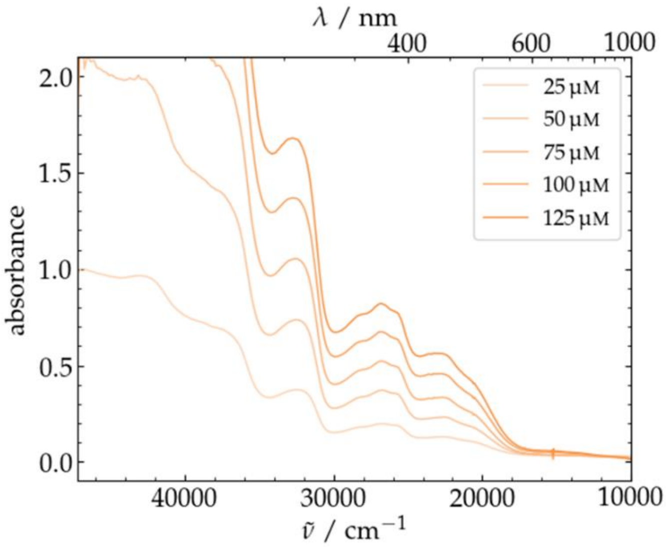

Figure S13. *UV/Vis spectra of THF solutions of **2b** at different concentrations at room temperature.*

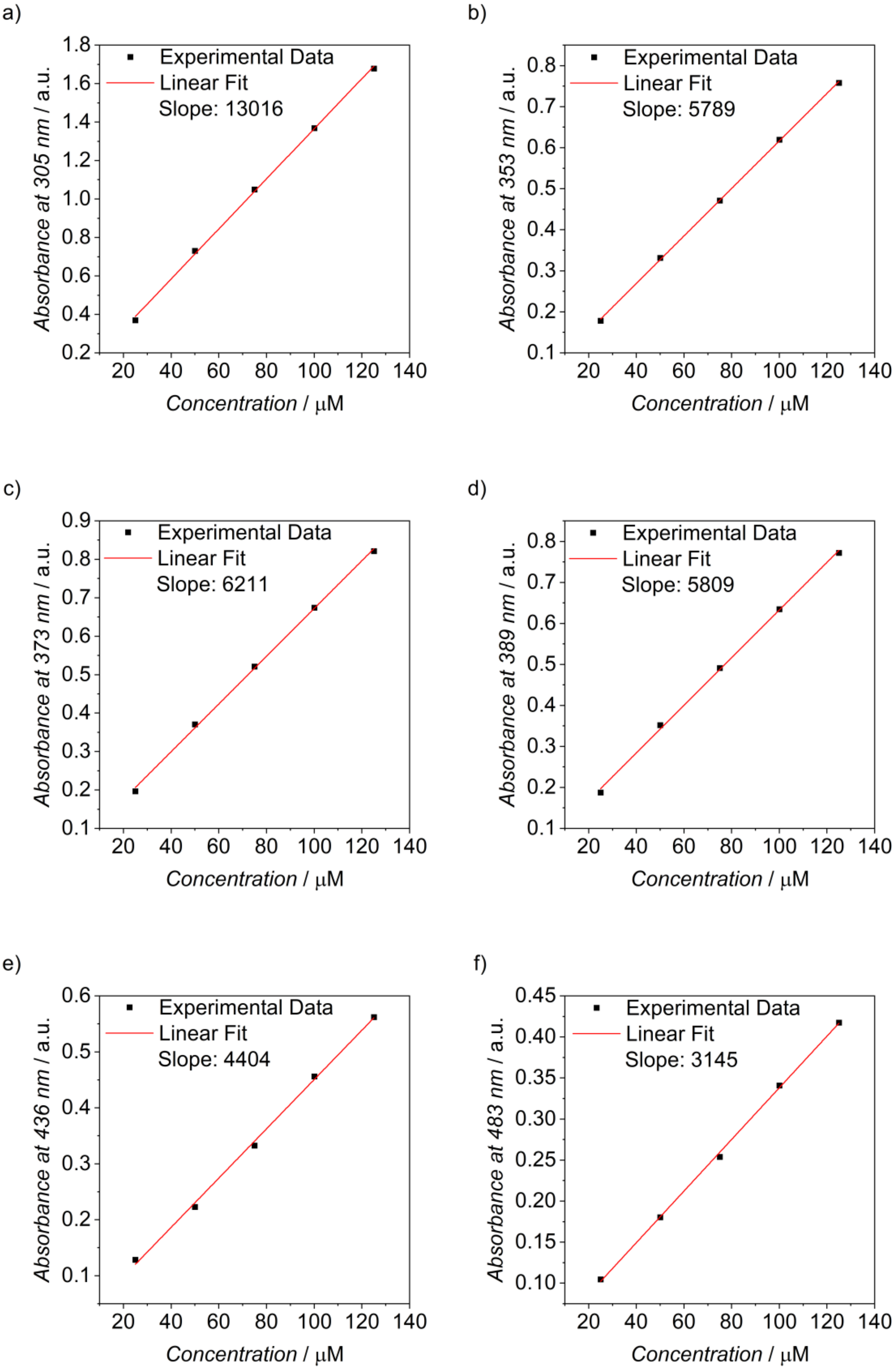

Figure S14. *Plots and linear fits of absorbance at a) 305, b) 353, c) 373, d) 389, e) 436, f) 483 nm versus concentrations of* ***2b*** *in THF at room temperature. The slope (in* $L\cdot mol^{-1}$*) of the linear fit has been used as an estimation of the molar extinction coefficients of the complex at the given wavelengths.*

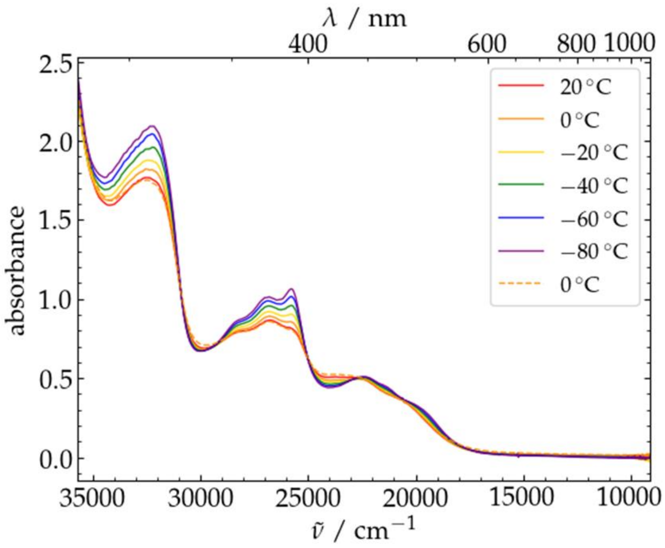

Figure S15. *UV/Vis spectra of a 0.1 m*M *THF solution of* ***2b*** *at different temperatures while cooling down as well as after heating up back to 0 °C (dashed line).*

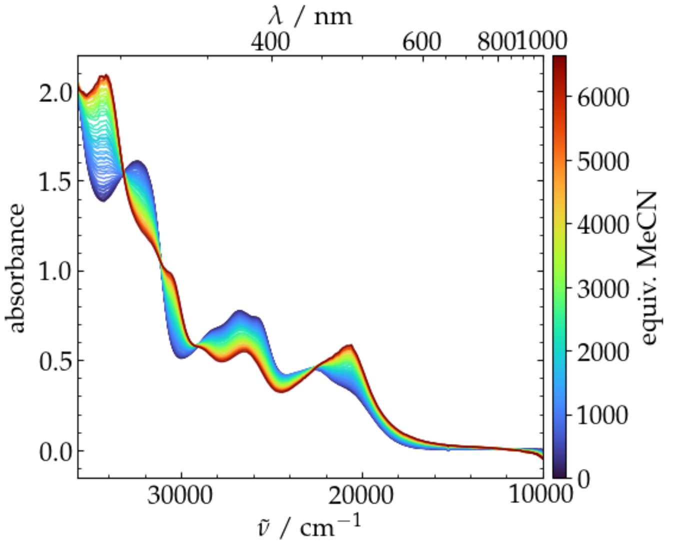

Figure S16. *UV/Vis spectra of a 0.1 m*M *THF solution of* ***2b*** *at 20 °C during addition of* CH_3CN.

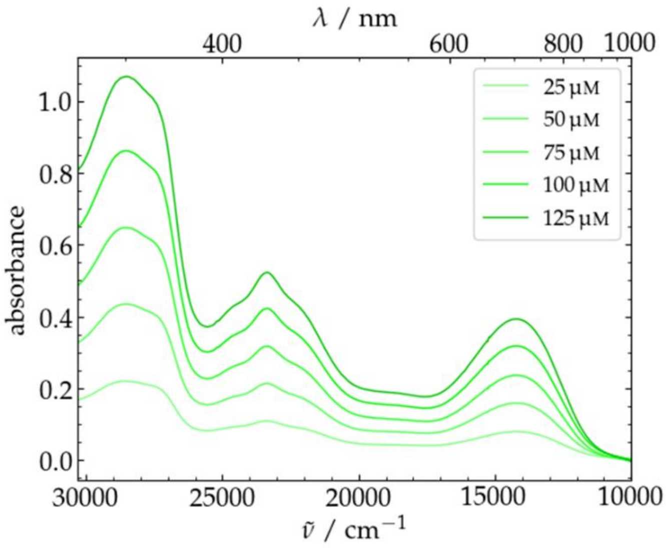

Figure S17. *UV/Vis spectra of acetone solutions of* ***3b*** *at different concentrations at room temperature.*

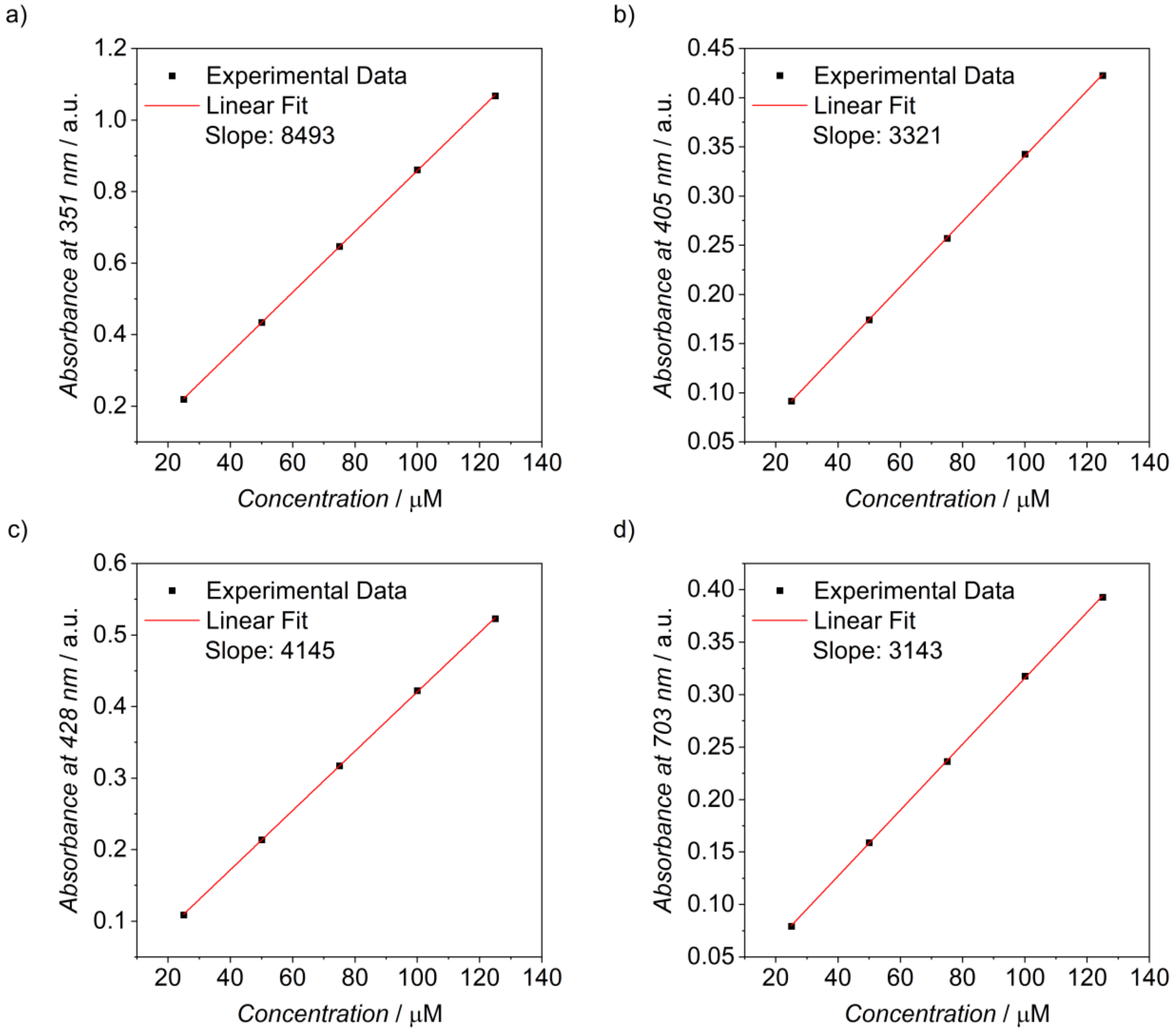

Figure S18. *Plots and linear fits of absorbance at a) 351, b) 405, c) 428, d) 703 nm versus concentrations of* ***3b*** *in acetone at room temperature. The slope (in L·mol^{-1}) of the linear fit has been used as an estimation of the molar extinction coefficients of the complex at the given wavelengths.*

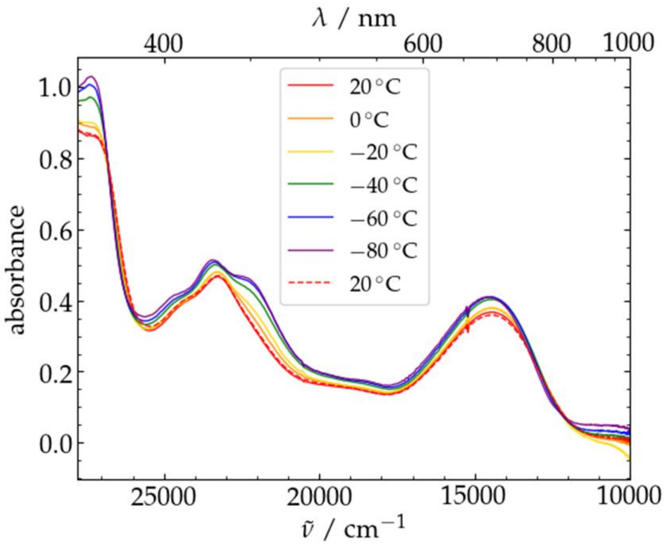

Figure S19. *UV/Vis spectra of a 0.1 mM THF solution of **3b** at different temperatures while cooling down as well as after heating up back to 20 °C (dashed line).*

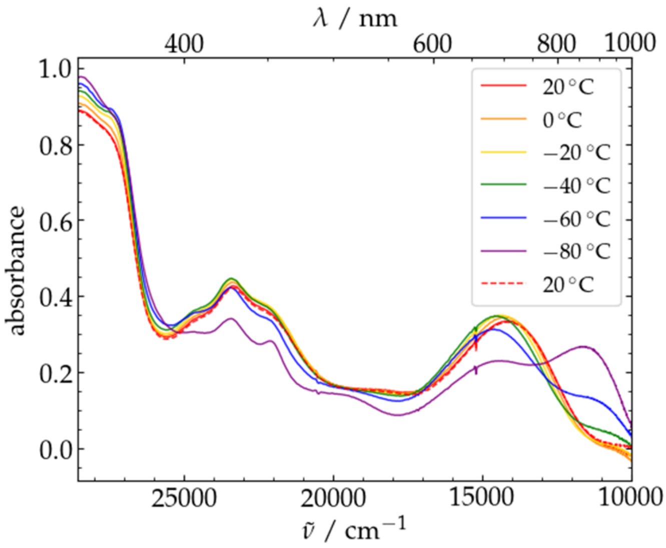

Figure S20. *UV/Vis spectra of a 0.1 mM acetone solution of **3b** at different temperatures while cooling down as well as after heating up back to 20 °C (dashed line).*

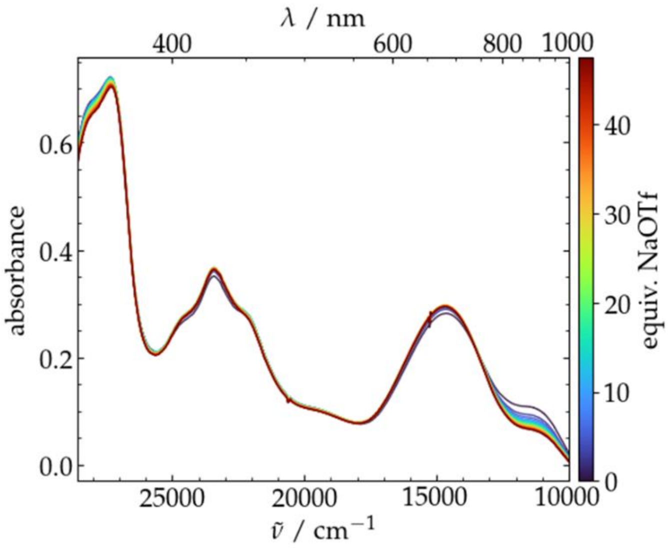

Figure S21. *UV/Vis spectra of a 0.1 mM acetone solution of* ***3b*** *at −60 °C during addition of NaOTf.*

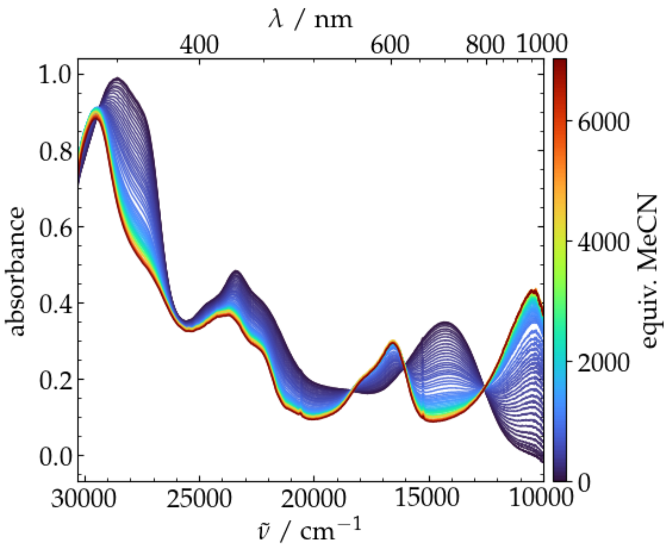

Figure S22. *UV/Vis spectra of a 0.1 mM acetone solution of* ***3b*** *at 20 °C during addition of* CH_3CN.

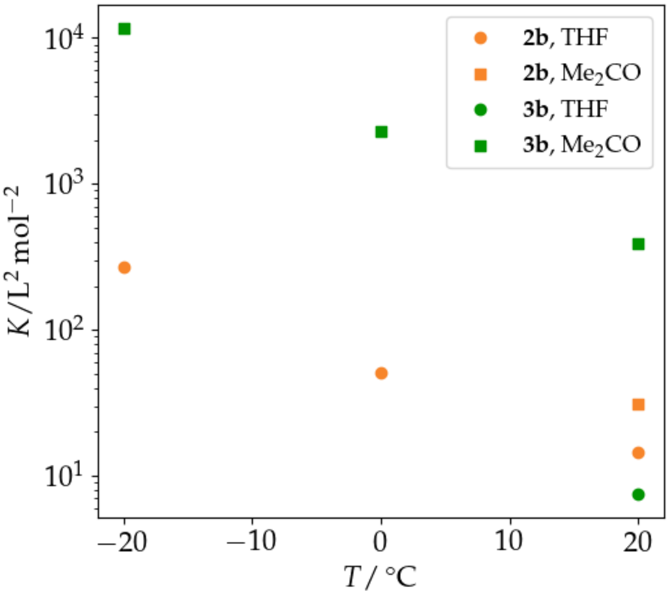

Figure S23. *Plot of the complex formation constants K_2 for the coordination of CH_3CN to* ***2b*** *and* ***3b*** *to give* ***2a*** *and* ***3a****, respectively, in different solvents at different temperatures as indicated in the inset.*

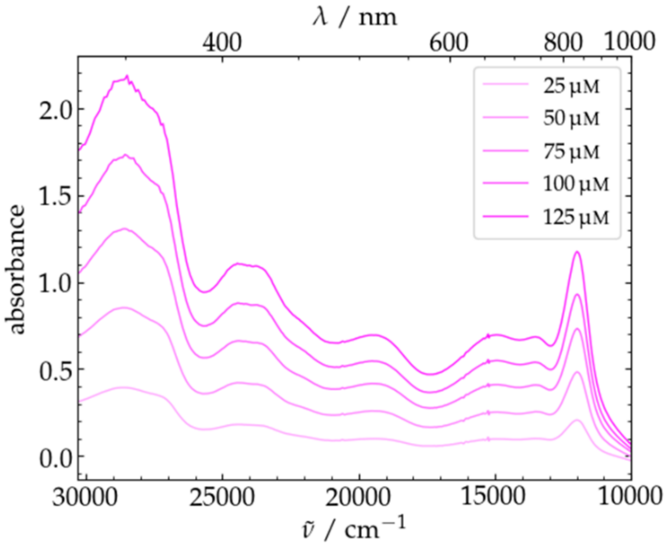

Figure S24. *UV/Vis spectra of acetone solutions of* ***4b*** *at different concentrations at −35 °C.*

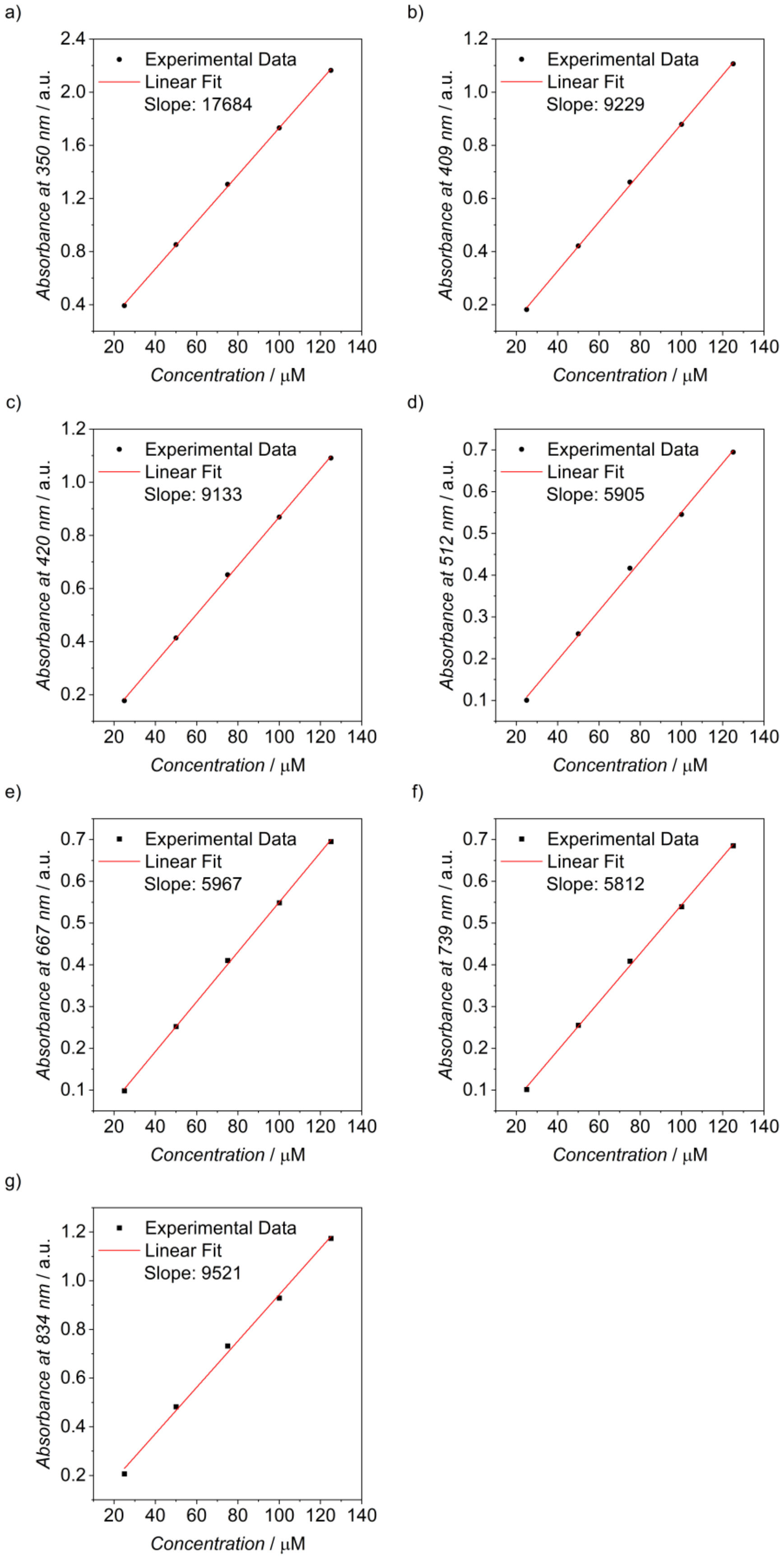

Figure S25. *Plots and linear fits of absorbance at a) 350, b) 409, c) 420, d) 512, e) 667, f) 739, g) 834 nm versus concentrations of* ***4b*** *in acetone at −35 °C. The slope (in L·mol*$^{-1}$*) of the linear fit has been used as an estimation of the molar extinction coefficients of the complex at the given wavelengths.*

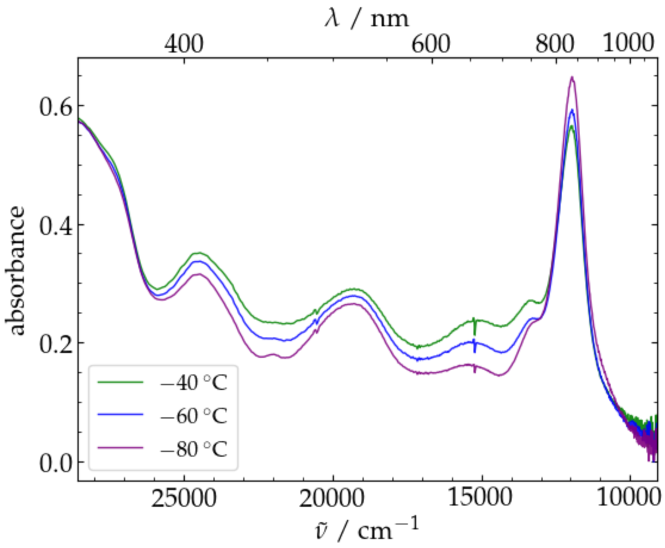

Figure S26. *UV/Vis spectra of an acetone solution of **4b** at different temperatures while warming from –80°C to –40°C.*

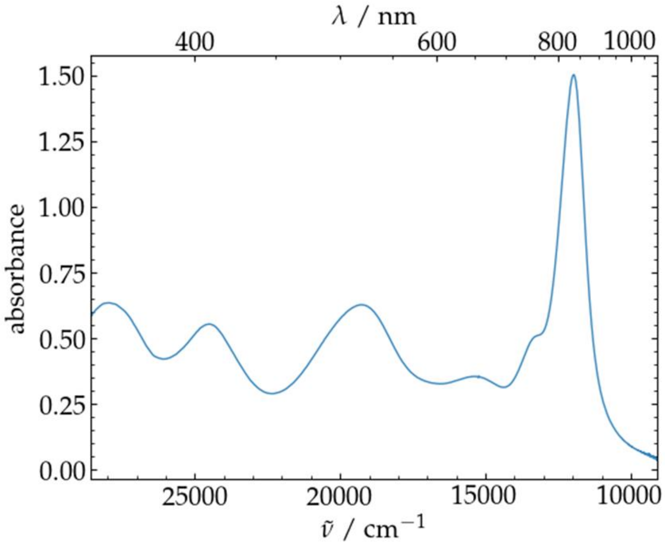

Figure S27. *UV/Vis spectrum of a 0.1 mM acetone solution of **4a** at −20 K.*

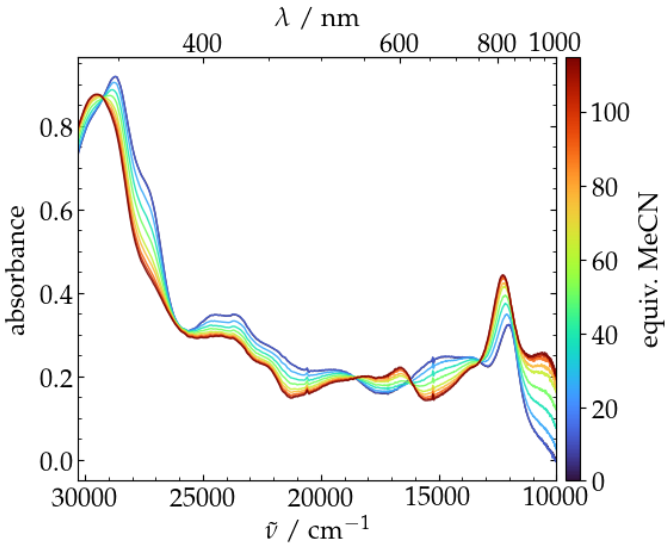

Figure S28. UV/Vis spectra of an ~0.1 mM acetone solution of **4b** at −40 °C during addition of CH_3CN.

3. NMR Spectroscopy

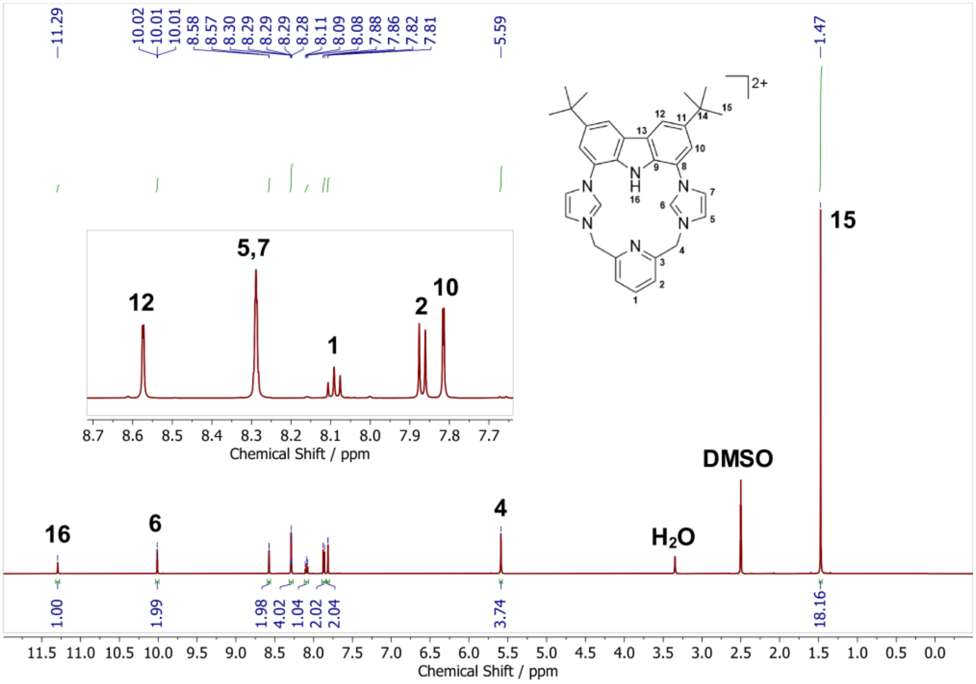

Figure S29. *^{1}H-NMR spectrum (500 MHz) of [LH_3](OTf)$_2$ in DMSO-d6 at room temperature.*

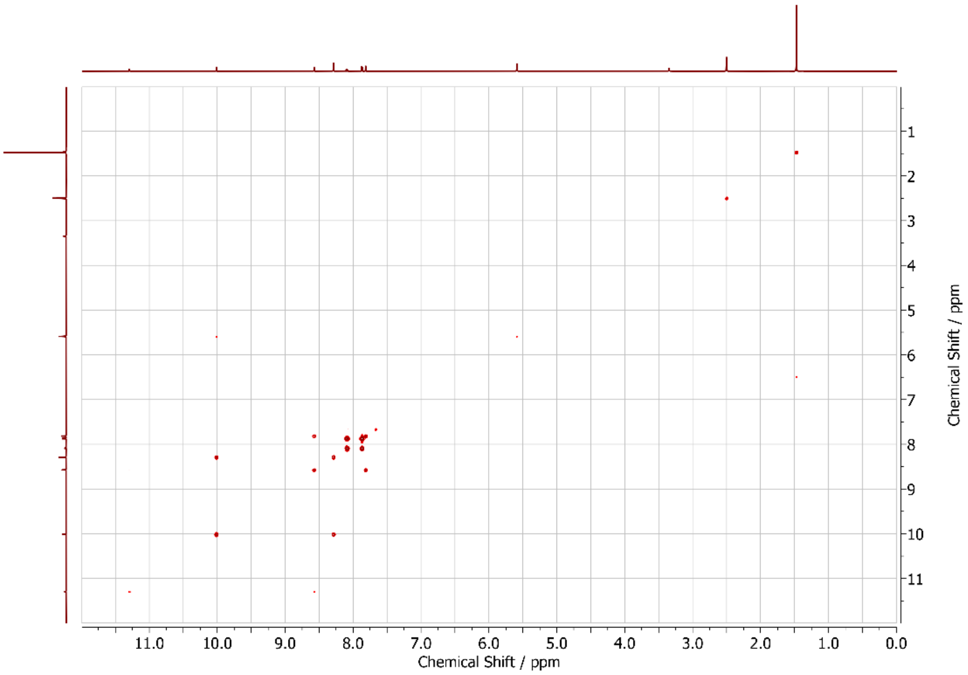

Figure S30. *^{1}H-^{1}H COSY NMR spectrum (600 MHz) of [LH_3](OTf)$_2$ in DMSO-d6 at room temperature.*

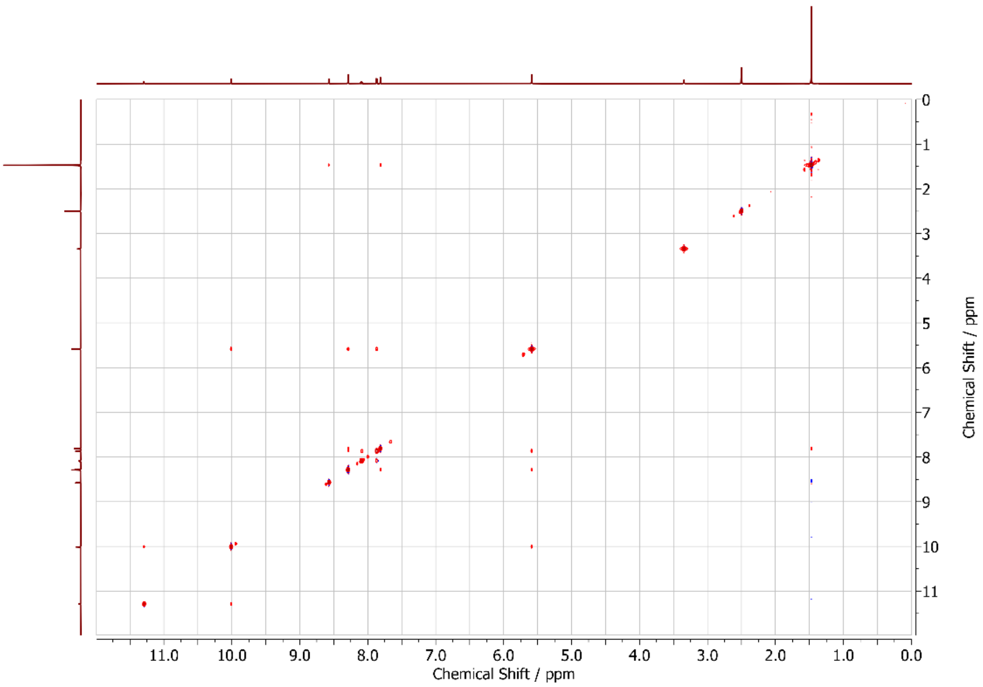

Figure S31. *^{1}H NOESY NMR spectrum (600 MHz) of $[LH_3](OTf)_2$ in DMSO-d6 at room temperature.*

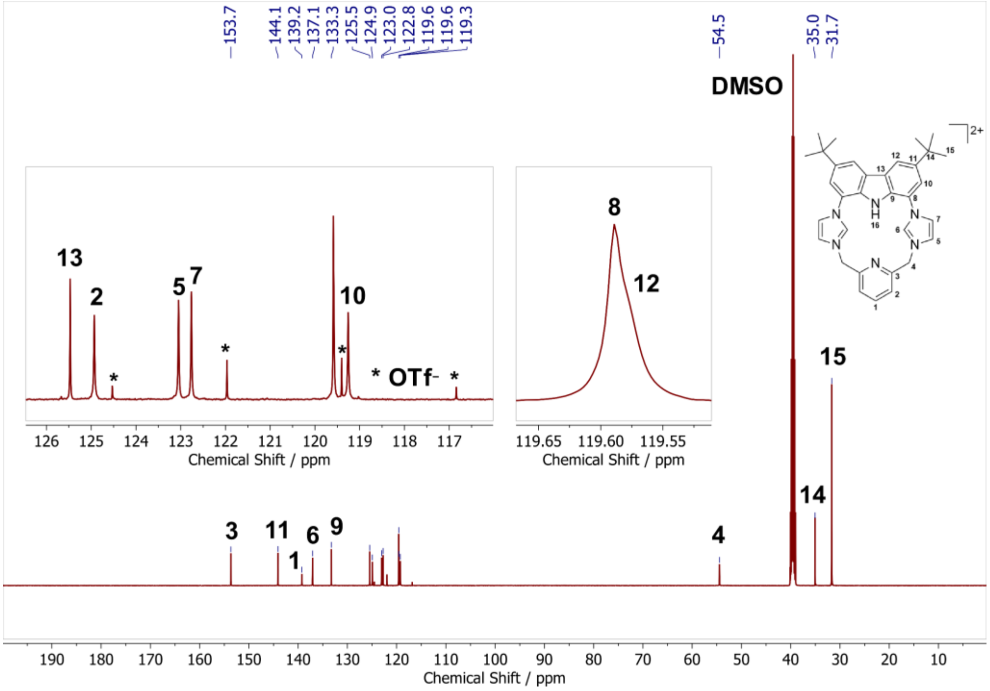

Figure S32. *$^{13}C\{^{1}H\}$ NMR spectrum (126 MHz) of $[LH_3](OTf)_2$ in DMSO-d6 at room temperature.*

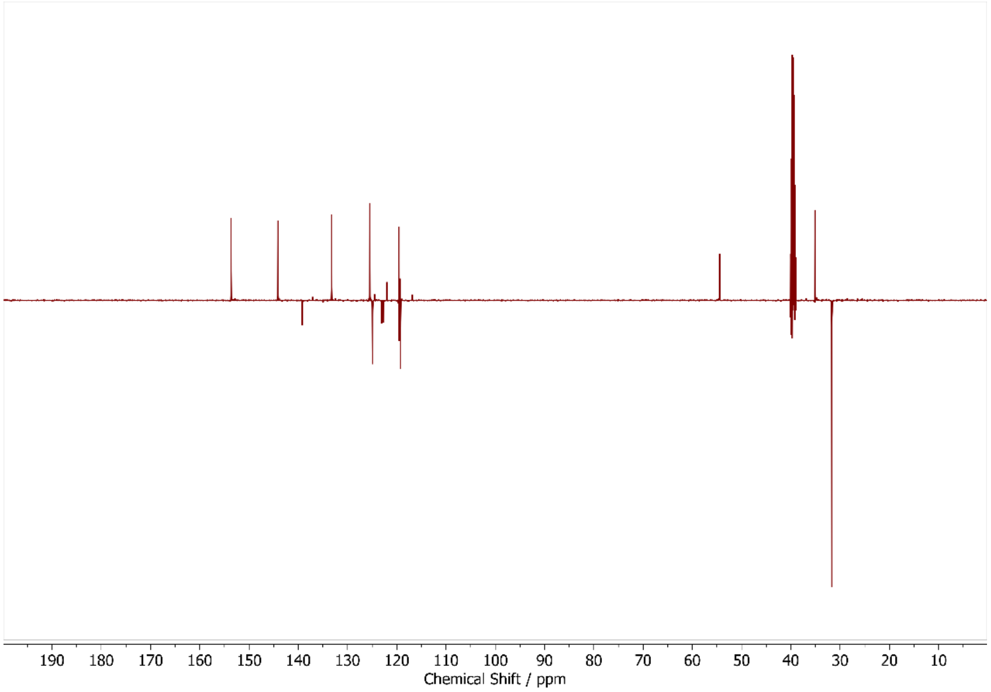

Figure S33. *$^{13}C\{^{1}H\}$ APT NMR spectrum (126 MHz) of $[LH_3](OTf)_2$ in DMSO-d6 at room temperature.*

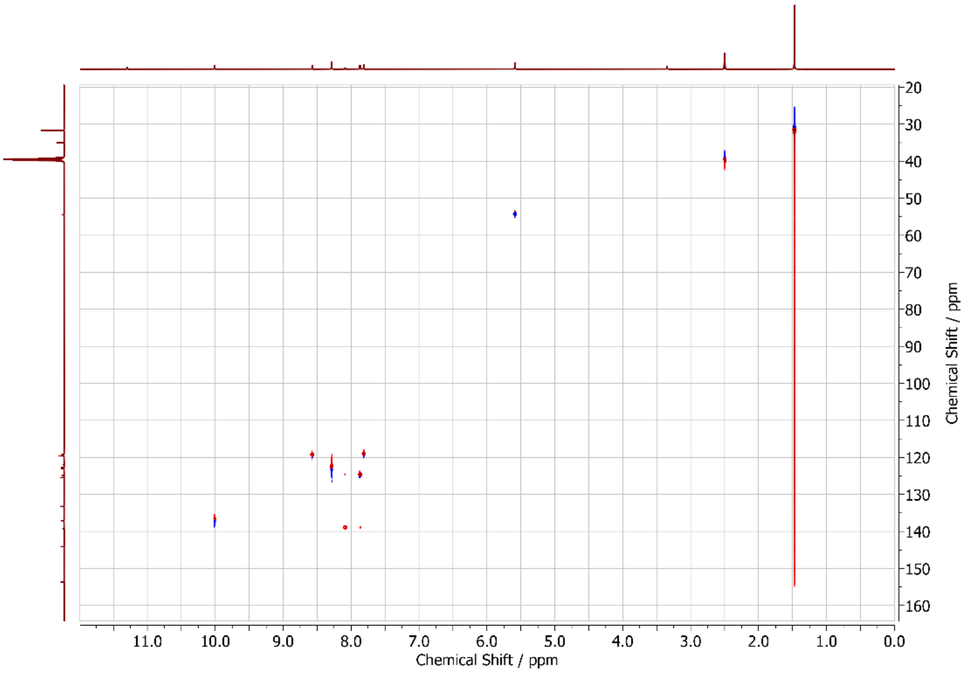

Figure S34. *^{1}H-^{13}C HSQC NMR spectrum (600 MHz) of $[LH_3](OTf)_2$ in DMSO-d6 at room temperature.*

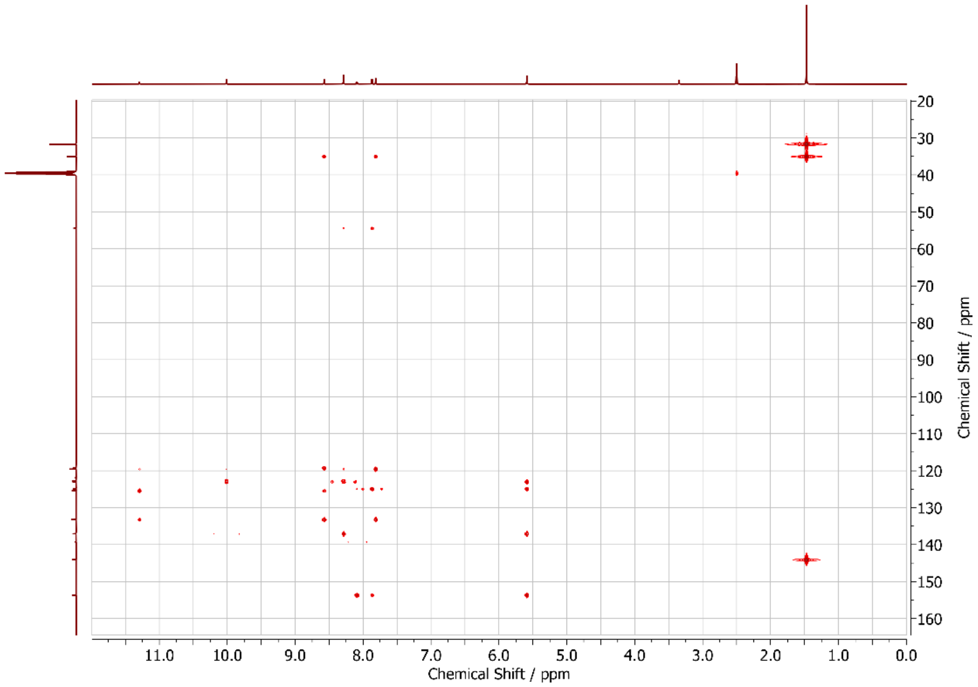

Figure S35. *^{1}H-^{13}C HMBC NMR spectrum (600 MHz) of $[LH_3](OTf)_2$ in DMSO-d6 at room temperature.*

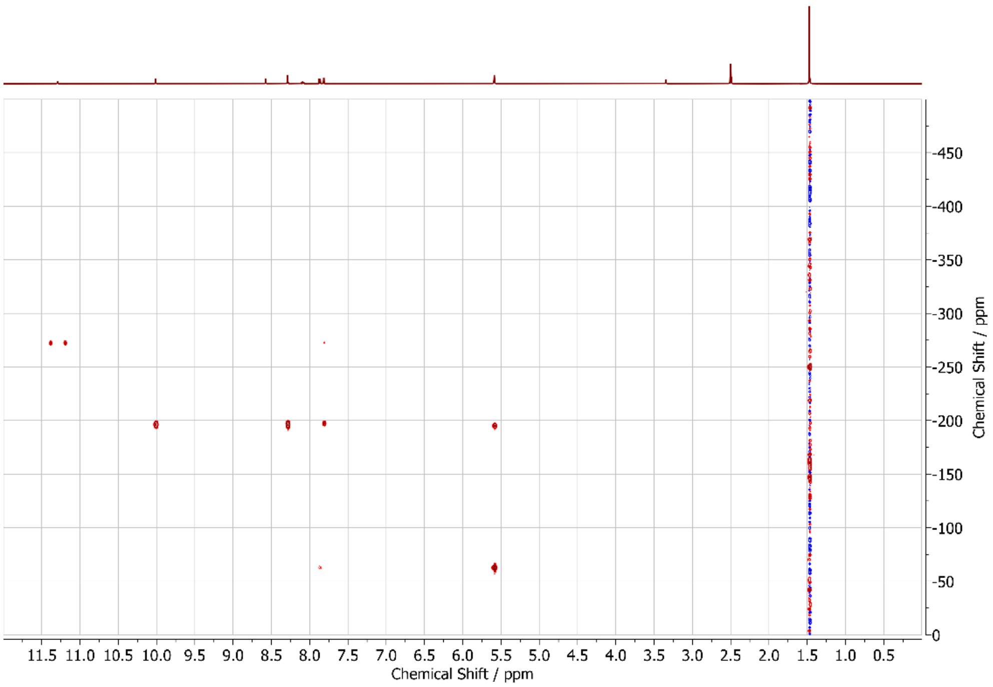

Figure S36. *^{1}H-^{15}N HMBC NMR spectrum (500 MHz) of $[LH_3](OTf)_2$ in DMSO-d6 at room temperature.*

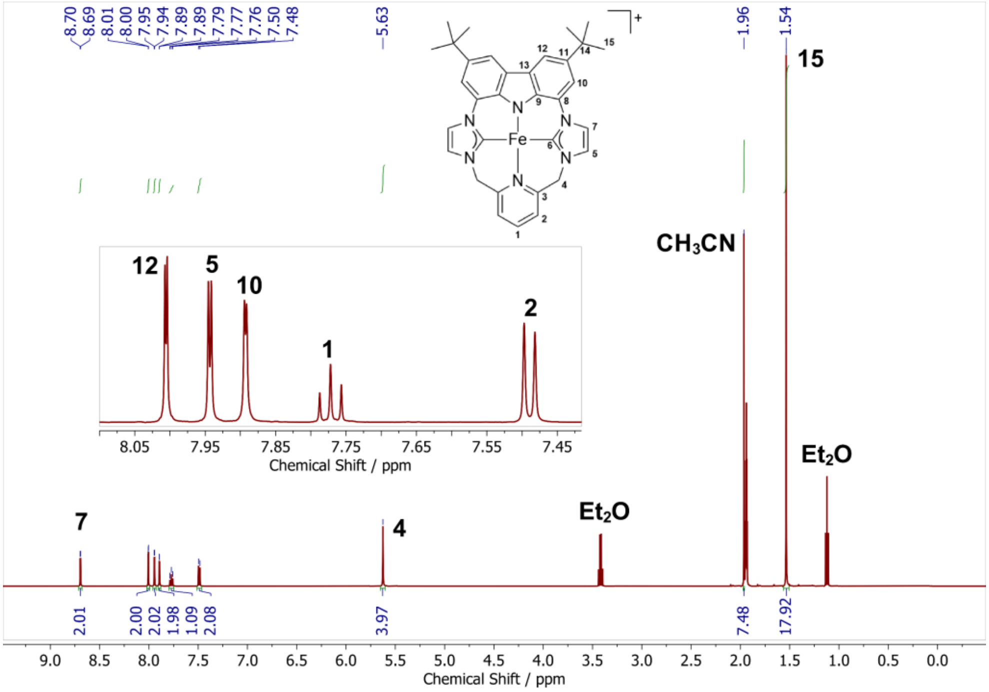

Figure S37. *^{1}H-NMR spectrum (500 MHz) of* ***2a*** *in CD_3CN at room temperature.*

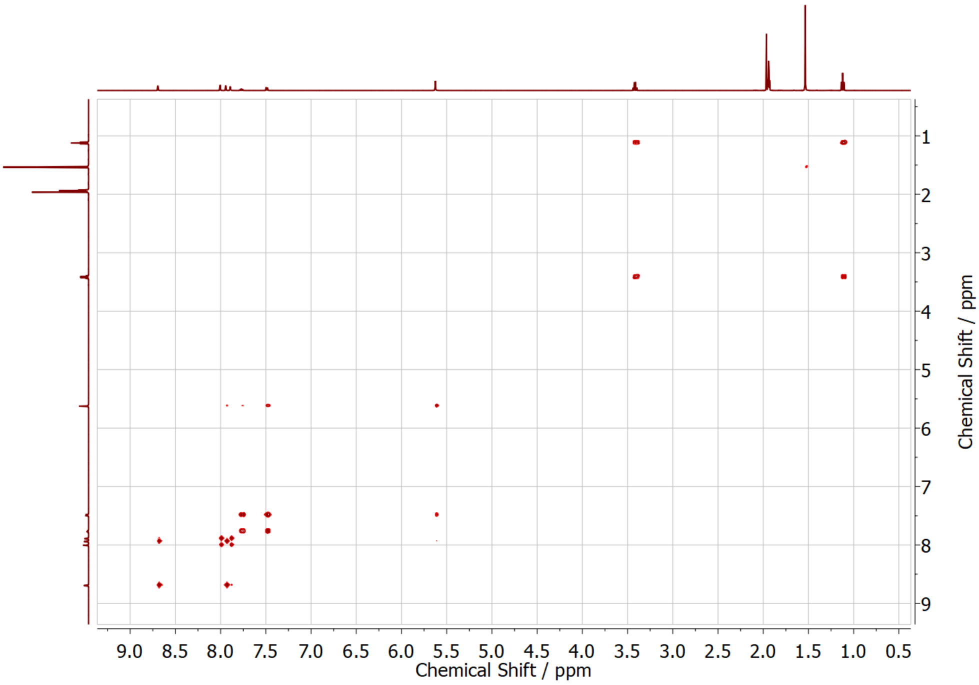

Figure S38. *^{1}H-^{1}H COSY NMR spectrum (500 MHz) of* ***2a*** *in CD_3CN at room temperature.*

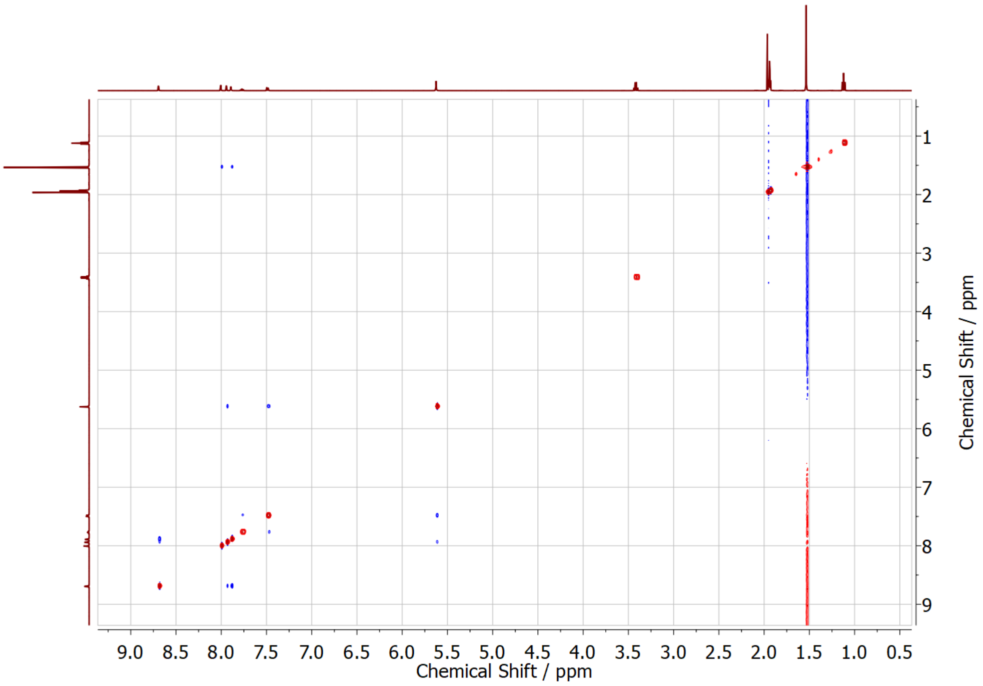

Figure S39. *^{1}H NOESY NMR spectrum (500 MHz) of **2a** in CD_3CN at room temperature.*

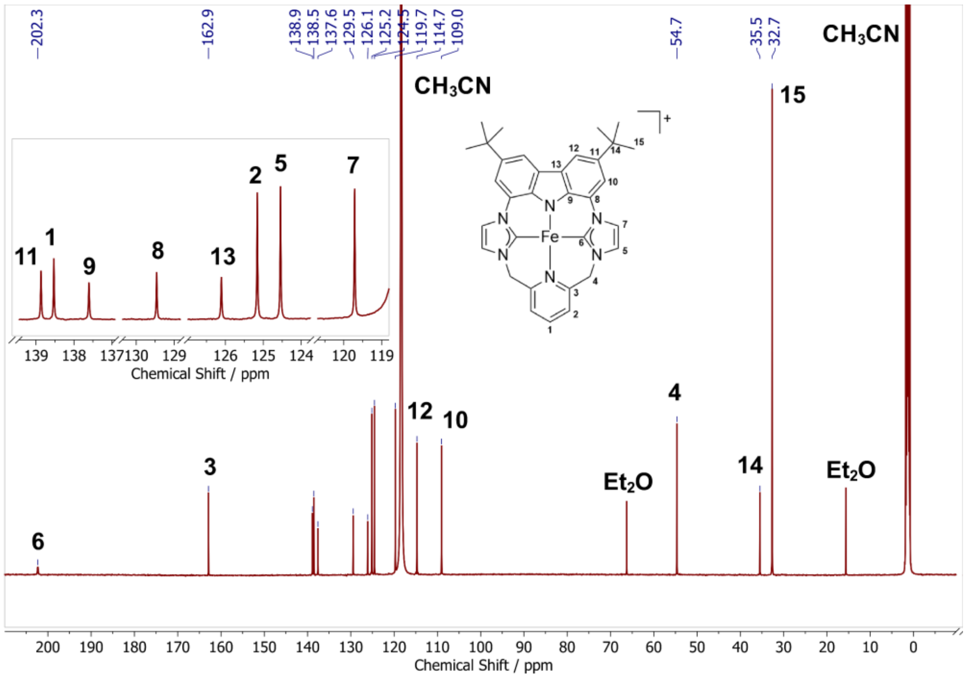

Figure S40. *^{13}C{^{1}H}-NMR spectrum (126 MHz) of **2a** in CD_3CN at room temperature.*

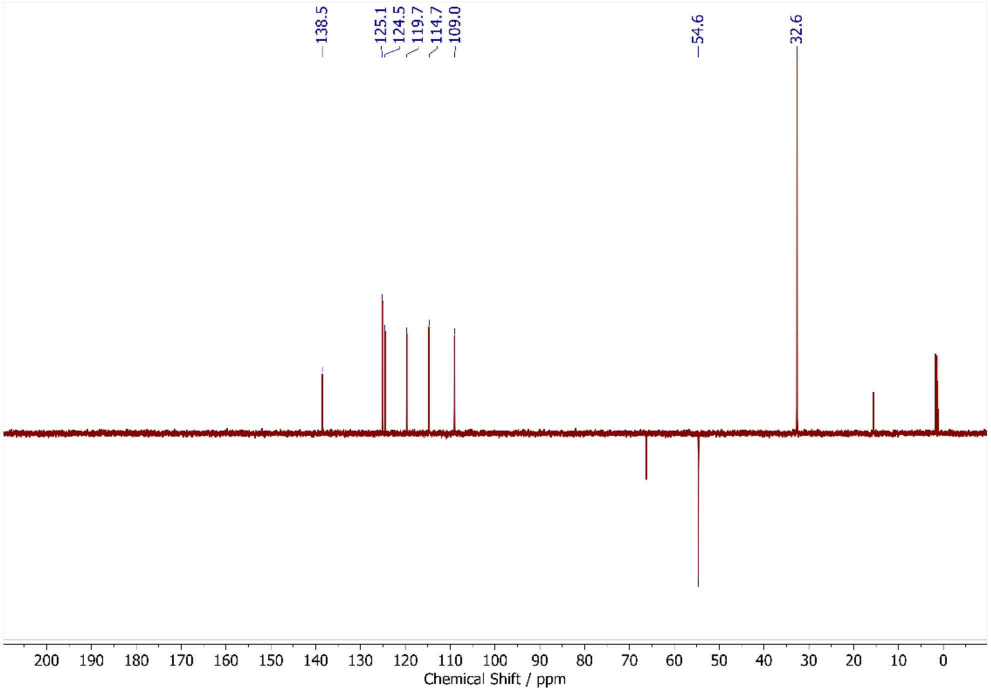

Figure S41. *$^{13}C\{^{1}H\}$ DEPT135 NMR spectrum (126 MHz) of* ***2a*** *in CD_3CN at room temperature.*

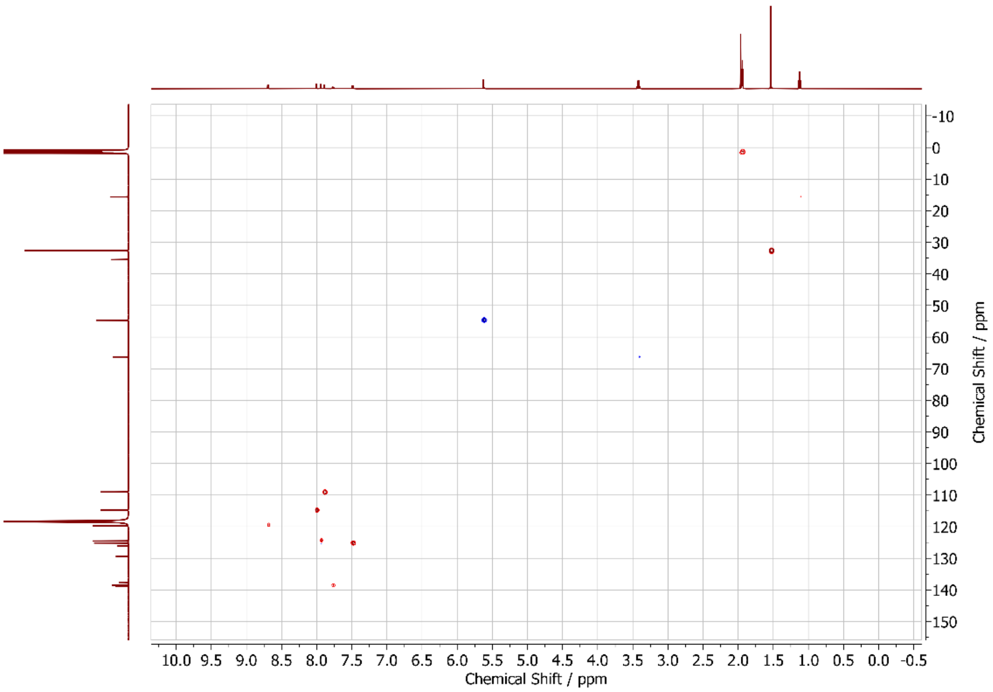

Figure S42. *^{1}H-^{13}C HSQC NMR spectrum (500 MHz) of* ***2a*** *in CD_3CN at room temperature.*

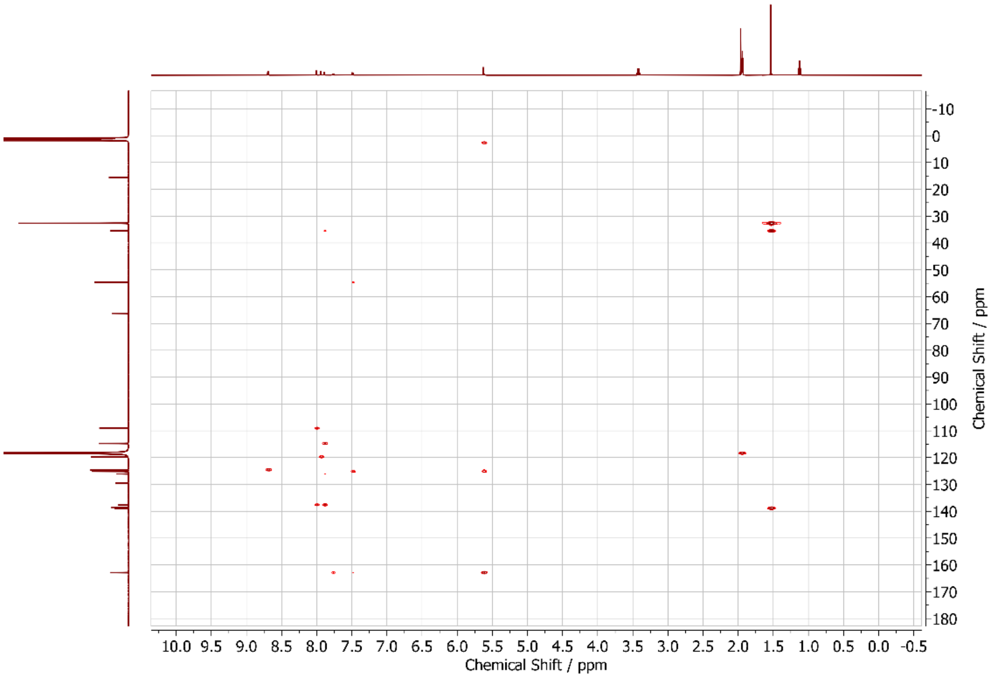

Figure S43. *^{1}H-^{13}C HMBC NMR spectrum (500 MHz) of* ***2a*** *in CD_3CN at room temperature.*

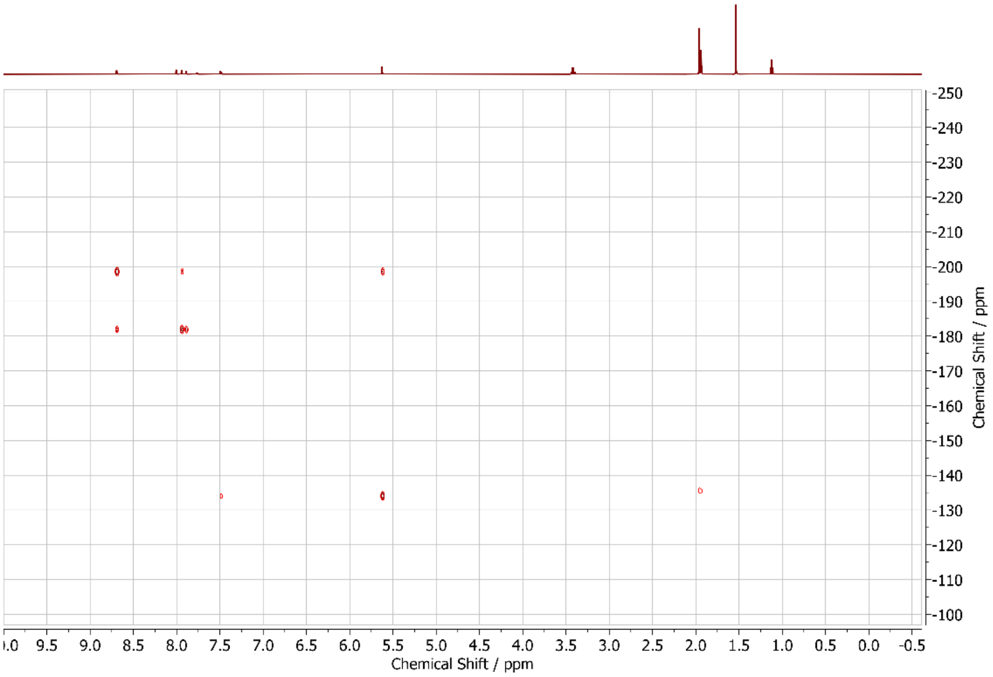

Figure S44. *^{1}H-^{15}N HMBC NMR spectrum (500 MHz) of* ***2a*** *in CD_3CN at room temperature.*

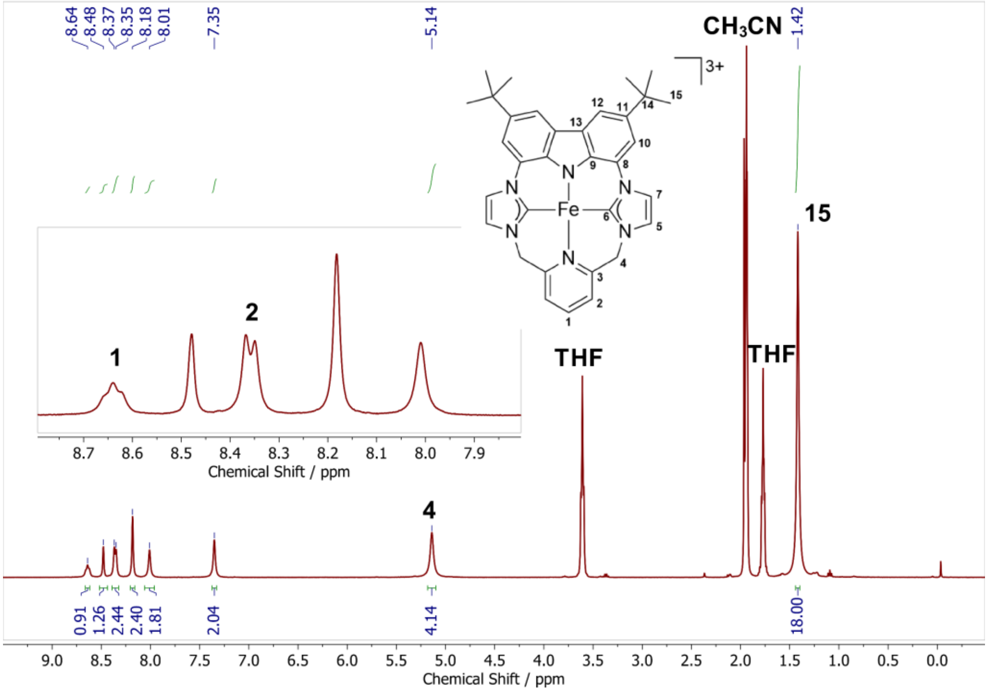

Figure S45. *^{1}H-NMR spectrum (400 MHz) of **4a** in CD_3CN at −35 °C.*

4. IR Spectroscopy

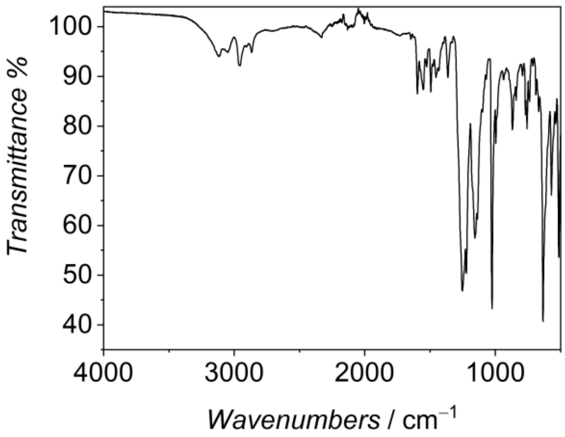

Figure S46. *ATR-IR spectrum of solid $[LH_3](OTf)_2$.*

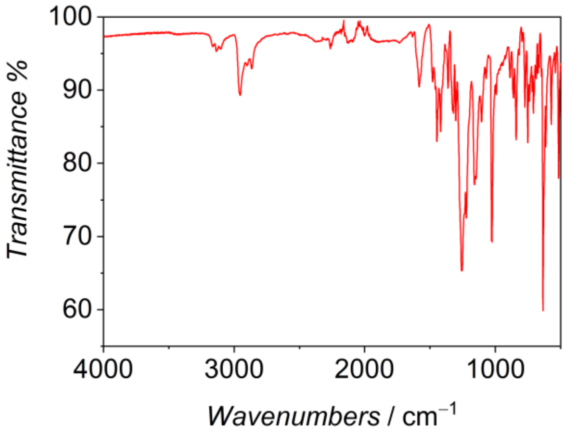

Figure S47. *ATR-IR spectrum of solid **2a**.*

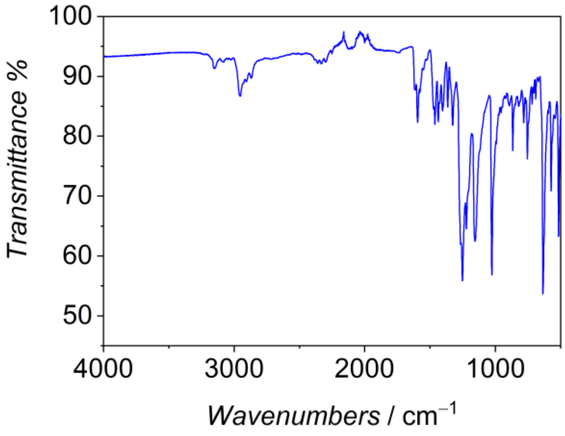

Figure S48. *ATR-IR spectrum of solid **3a**.*

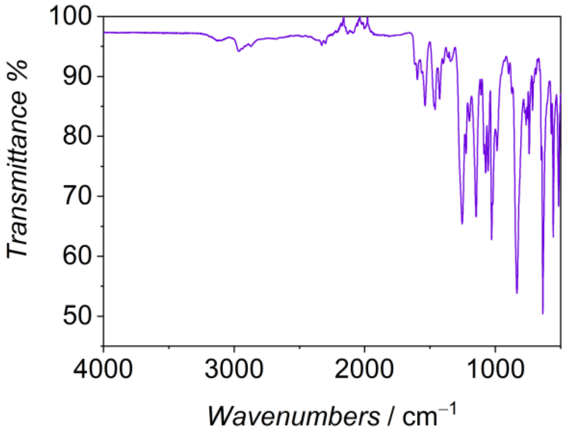

Figure S49. *ATR-IR spectrum of solid **4a**.*

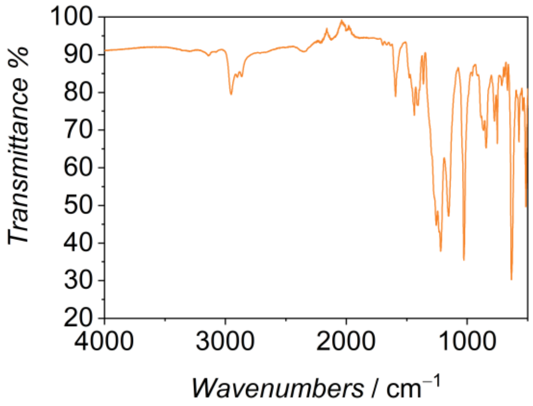

Figure S50. *ATR-IR spectrum of solid **2b**.*

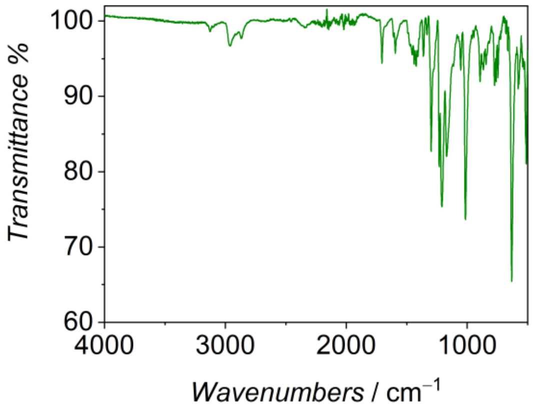

Figure S51. *ATR-IR spectrum of solid **3b**.*

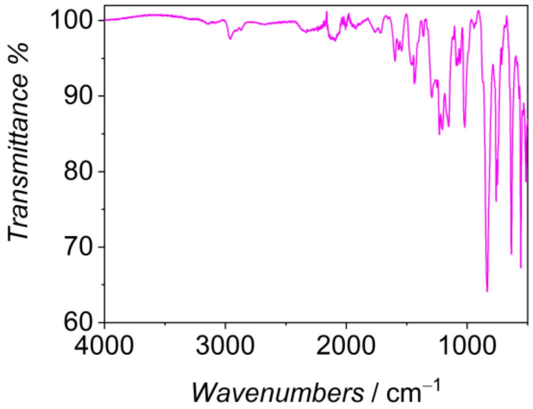

Figure S52. *ATR-IR spectrum of solid **4b**.*

5. Mössbauer Spectroscopy

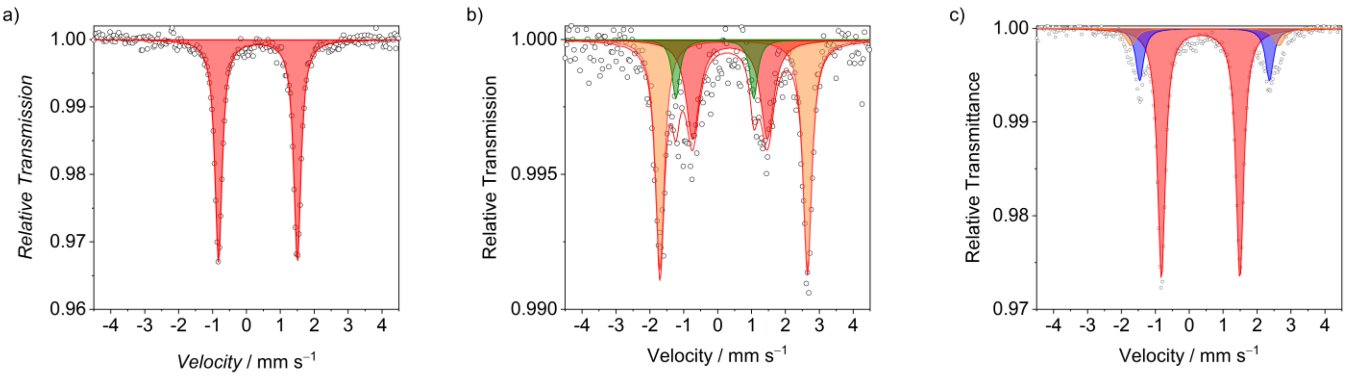

Figure S53. *Left: ^{57}Fe Mössbauer (MB) spectrum of solid* ***2a*** *at 80 K. Center: MB spectrum at 80 K of the product (**2a'**) obtained by exposing* ***2a*** *to vacuum for 3 h. Right: MB spectrum at 80 K of the product of dissolution of* ***2a'*** *in CH_3CN followed by reprecipitation.*

6. Magnetic Susceptibility Measurements

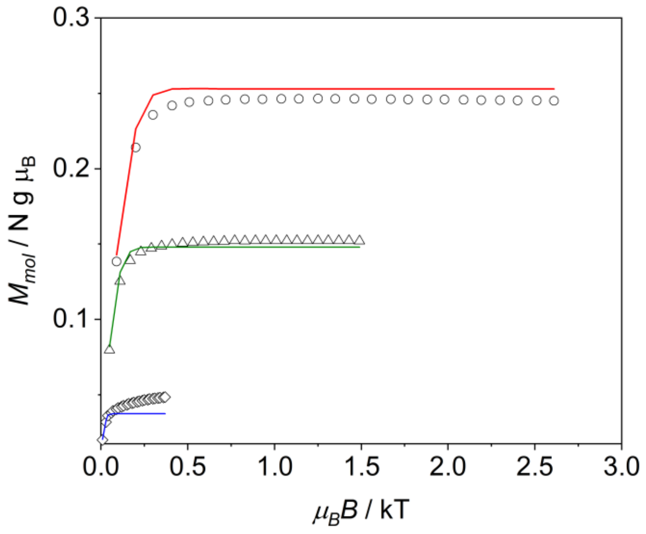

Figure S54. *Variable temperature – variable field (VTVH) magnetization measurements of a solid sample of* ***2b*** *at applied fields of 1.0 (diamonds and blue fit), 4.0 (triangles and green fit) and 7.0 T (circles and red fit).*

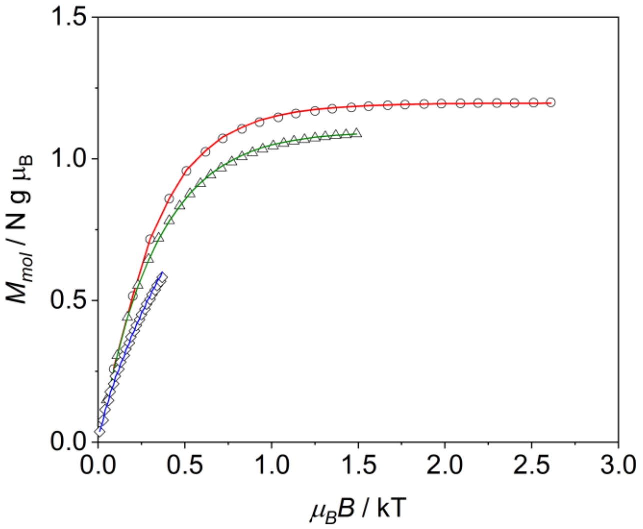

Figure S55. *Variable temperature – variable field (VTVH) magnetization measurements of a solid sample of* ***3b*** *at applied fields of 1.0 (diamonds and blue fit), 4.0 (triangles and green fit) and 7.0 T (circles and red fit).*

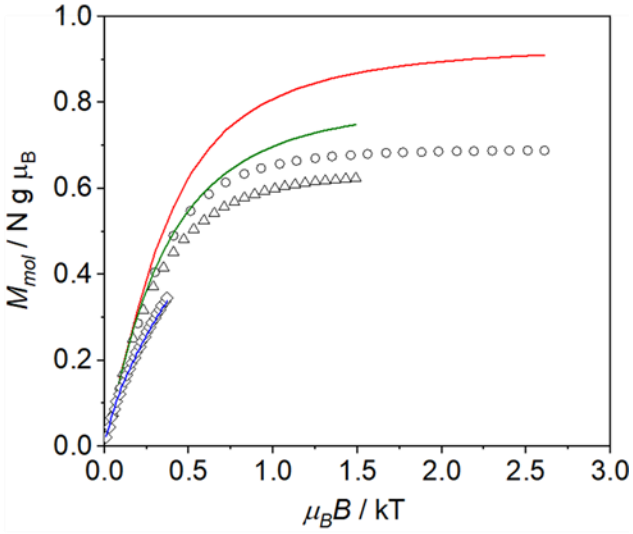

Figure S56. *Variable temperature – variable field (VTVH) magnetization measurements of a solid sample of* ***4b*** *at applied fields of 1.0 (diamonds and blue fit), 4.0 (triangles and green fit) and 7.0 T (circles and red fit)*

7. Electrochemistry

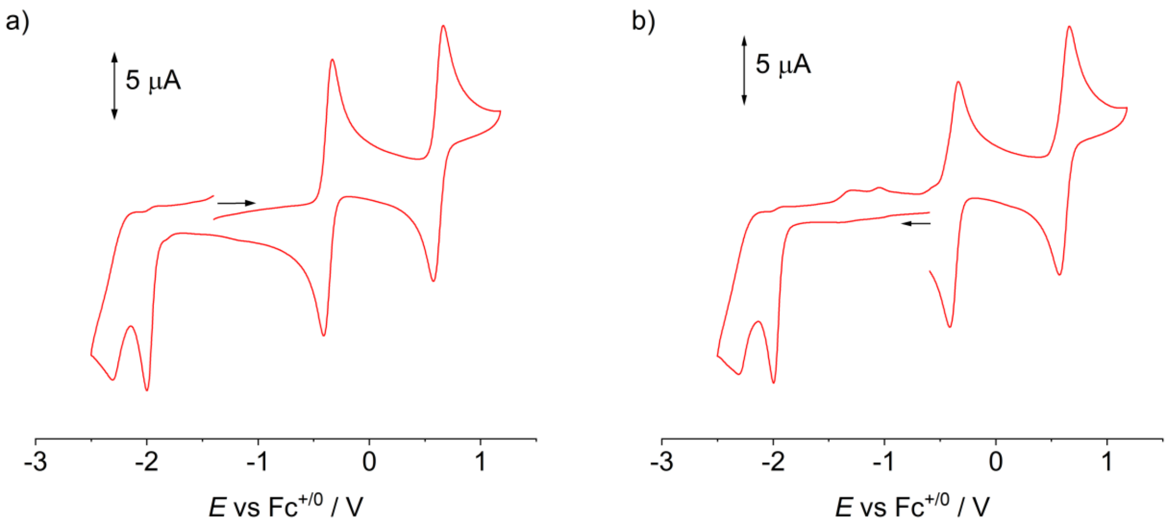

Figure S57. *Cyclic voltammograms of a 1 m*M *CH_3CN solution of crystalline* ***2a****, in 0.1* M *$^nBu_4NPF_6$ at room temperature: a) complete window scanned at 0.1 $V{\cdot}s^{-1}$ starting from −1.4 V and scanning anodically first; b) complete window scanned at 0.1 $V{\cdot}s^{-1}$ starting at the OCP and scanning cathodically first.*

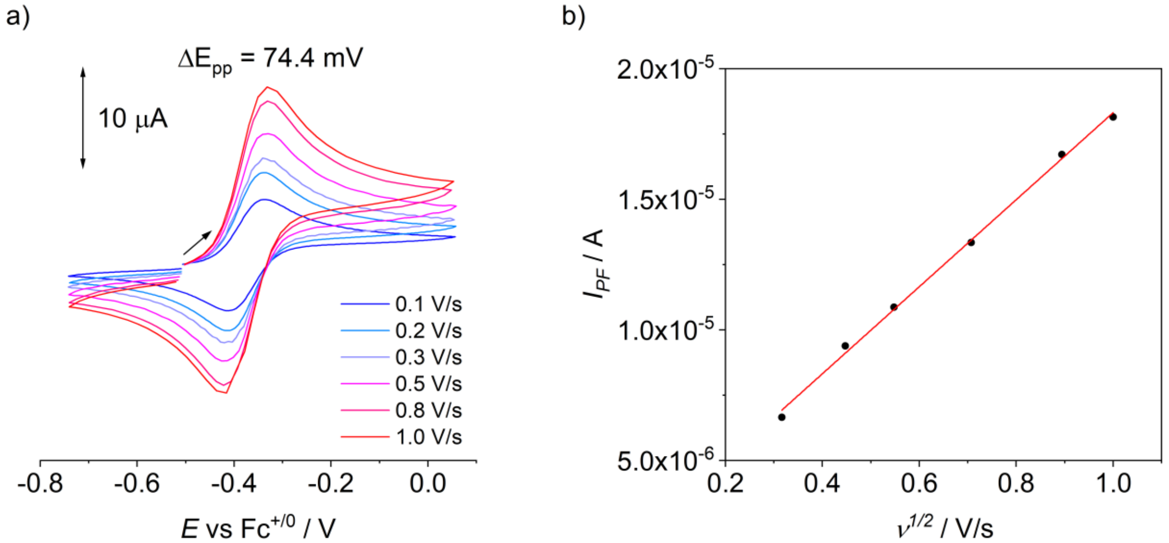

Figure S58. *Scan rate dependence of the first oxidative wave in the cyclic voltammogram of a 1 m*M *CH_3CN solution of crystalline* ***2a****, in 0.1* M *$^nBu_4NPF_6$ at room temperature: a) cyclic voltammograms and reported peak-to-peak separation of the first oxidative event (ΔE_{pp}) at 0.1 $V{\cdot}s^{-1}$; b) plot and linear fit of the maximum of current intensity for the forward peak (I_{PF}) versus the square root of the scan rate ($\nu^{1/2}$).*

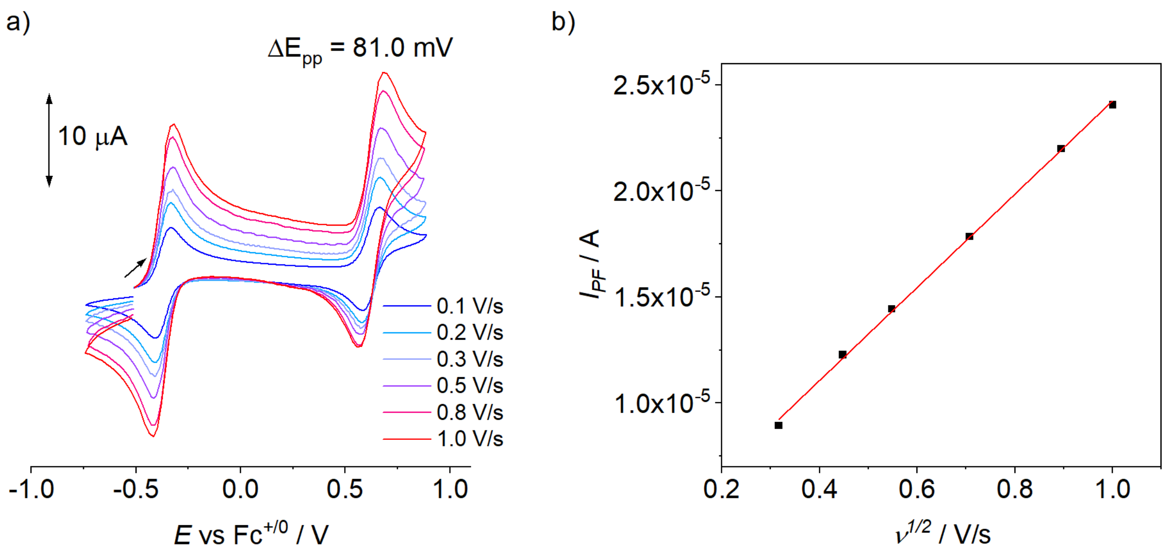

Figure S59. *Scan rate dependence of the oxidative waves in the cyclic voltammogram of a 1 mM CH_3CN solution of crystalline* ***2a****, in 0.1 M $^nBu_4NPF_6$ at room temperature. a) cyclic voltammograms and reported peak-to-peak separation of the second oxidative event (ΔE_{pp}) at 0.1 $V{\cdot}s^{-1}$; b) plot and linear fit of the maximum of current intensity for the forward peak (I_{PF}) versus the square root of the scan rate ($\nu^{1/2}$) for the second oxidation.*

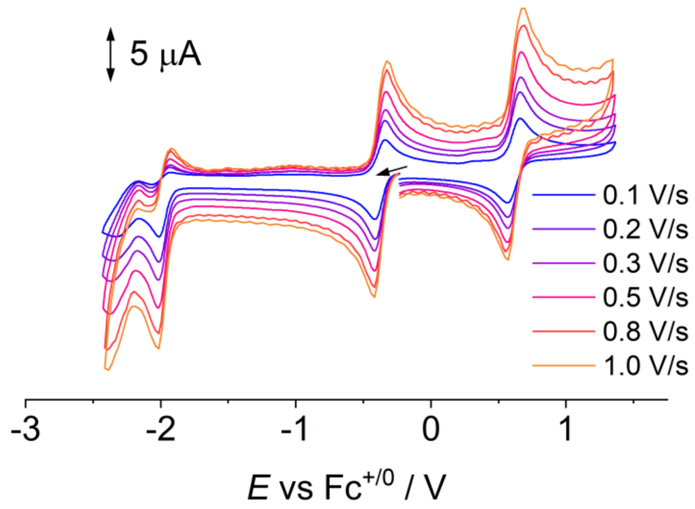

Figure S60. *Cyclic voltammograms of a 1 mM CH_3CN solution of crystalline* ***3a*** *in 0.1 M $^nBu_4NPF_6$ at room temperature and their scan rate dependence. The measurements were started at the OCP and scanned cathodically first.*

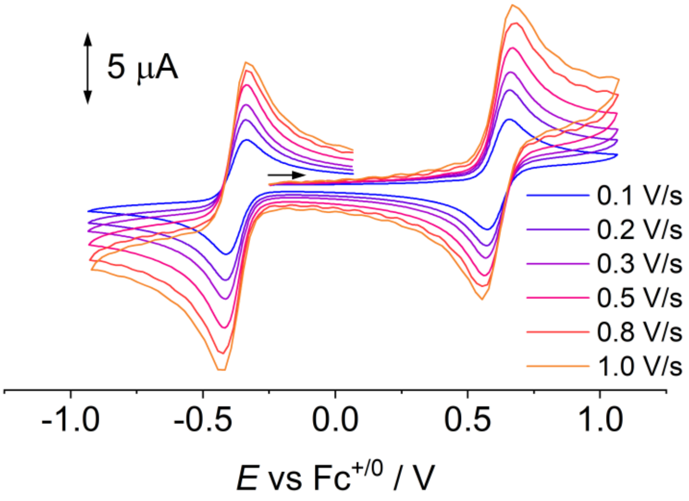

Figure S61. *Cyclic voltammograms of a 1 mM CH_3CN solution of crystalline* ***3a****, in 0.1 M $^nBu_4NPF_6$ at room temperature and their scan rate dependence. The measurements were started at the OCP and scanned anodically first, extending the measurement beyond the first reduction.*

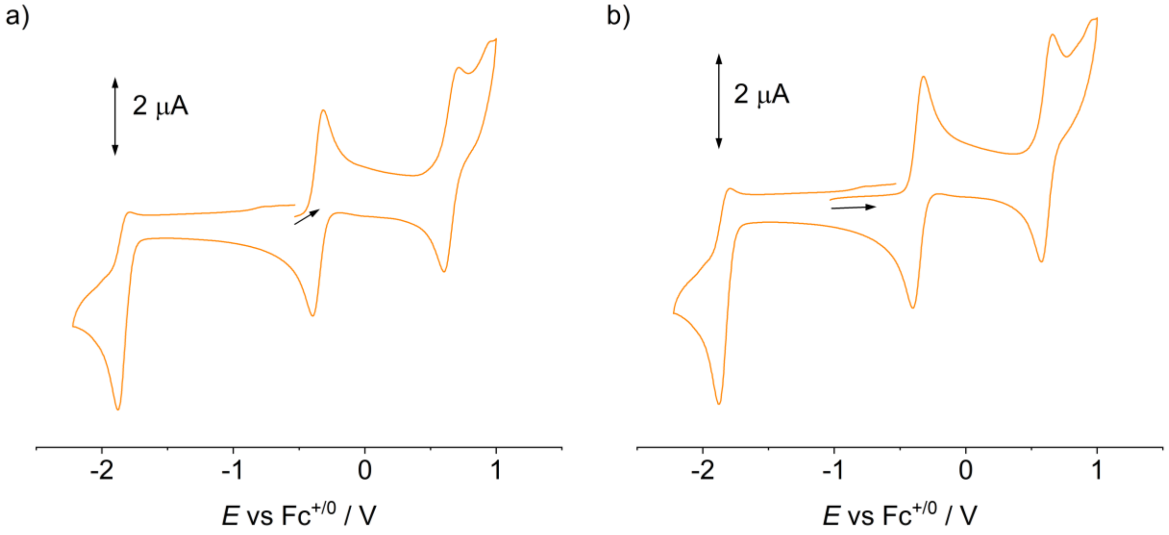

Figure S62. *Cyclic voltammograms of a 1 mM THF solution of* ***2b****, in 0.2 M $^nBu_4NPF_6$ at room temperature: a) complete window scanned at 0.1 $V{\cdot}s^{-1}$ starting at OCP and scanning anodically first; b) complete window scanned at 0.1 $V{\cdot}s^{-1}$ starting at −1.0 V and scanning anodically first.*

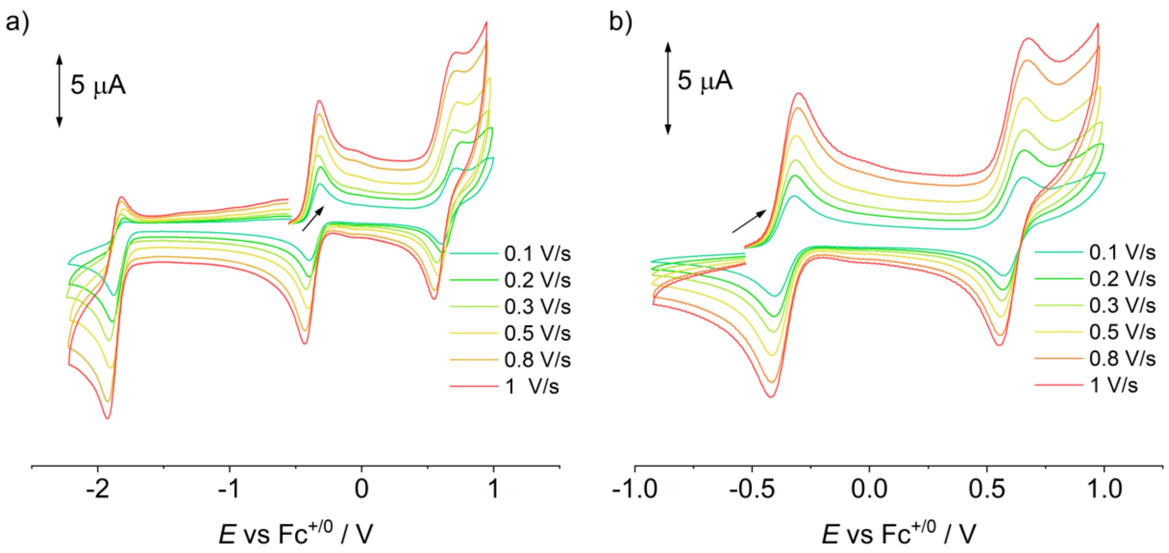

Figure S63. *Cyclic voltammograms of a 1 m*M *THF solution of crystalline* ***2b*** *in 0.2* M $^{n}Bu_4NPF_6$ *at room temperature and their scan rate dependence: a) complete window scanned starting from OCP; b) anodic portion only.*

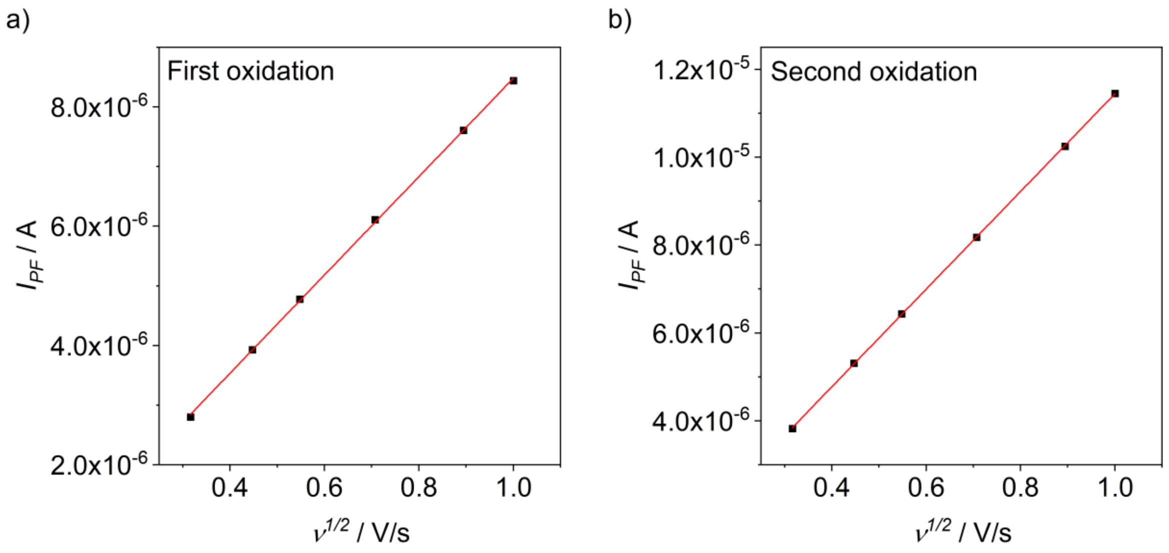

Figure S64. *Plot and linear fit of the maximum of current intensity for the forward peak (I_{PF}) versus the square root of the scan rate ($\nu^{1/2}$) for the first (a) and second oxidation (b) of complex* ***2b****.*

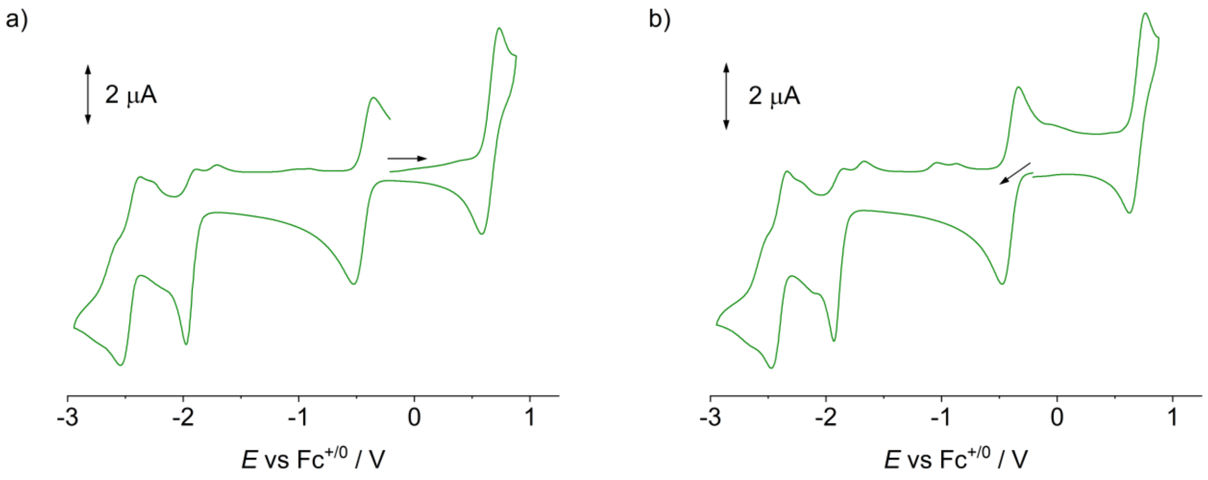

Figure S65. *Cyclic voltammograms of a 1 mM acetone solution of crystalline **3b**, in 0.1 M $^{n}Bu_4NPF_6$ at room temperature: a) complete window scanned at 0.1 V·s^{-1} starting from OCP and scanning anodically first; b) complete window scanned at 0.1 V·s^{-1} starting at the OCP and scanning cathodically first.*

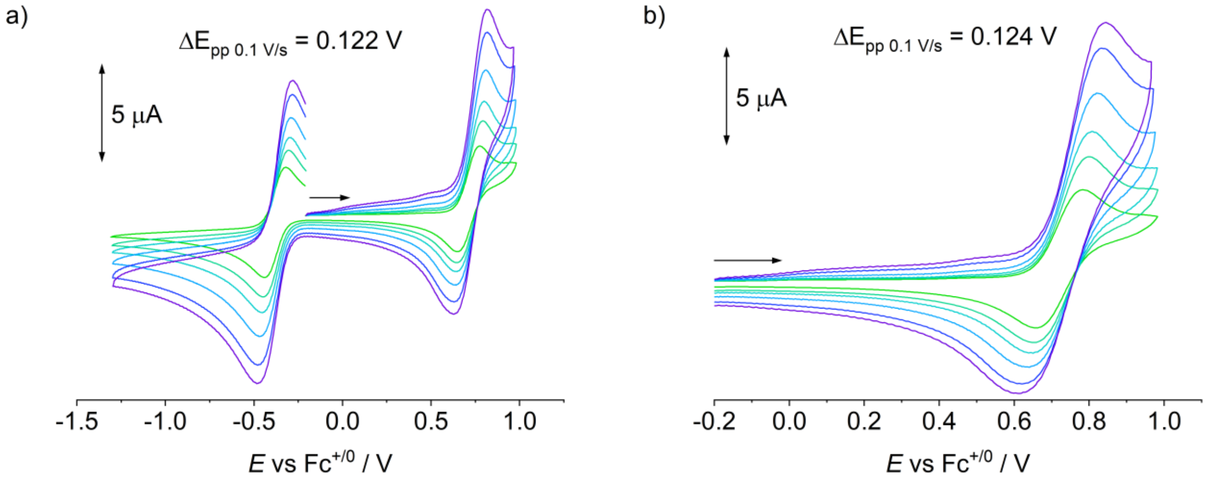

Figure S66. *Cyclic voltammograms of a 1 mM acetone solution of crystalline **3b**, in 0.1 M $^{n}Bu_4NPF_6$ at room temperature and their scan rate dependence: a) the measurement was started at the OCP and scanned anodically first, extending the measurement beyond the first reduction; b) anodic portion only.*

8. UV/Vis Spectroelectrochemistry

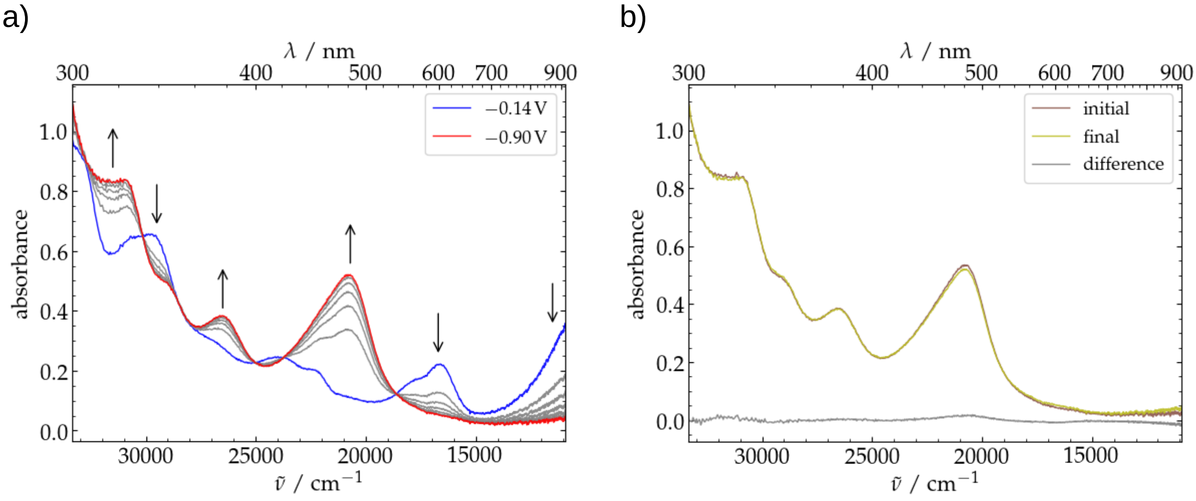

Figure S67. *UV/Vis spectroelectrochemistry of a 1 mM solution of complex* ***2a*** *in* CH_3CN *with 0.1 M* $^{n}Bu_4NPF_6$ *as electrolyte: a) re-reduction at an applied potential of −0.90 V (300 s), of the intermediate obtained oxidizing* ***2a*** *at −0.14 V (Figure 5) for 300 s; b) overlay of the initial spectrum of* ***2a*** *with the final spectrum obtained at the end of the experiment (600 s).*

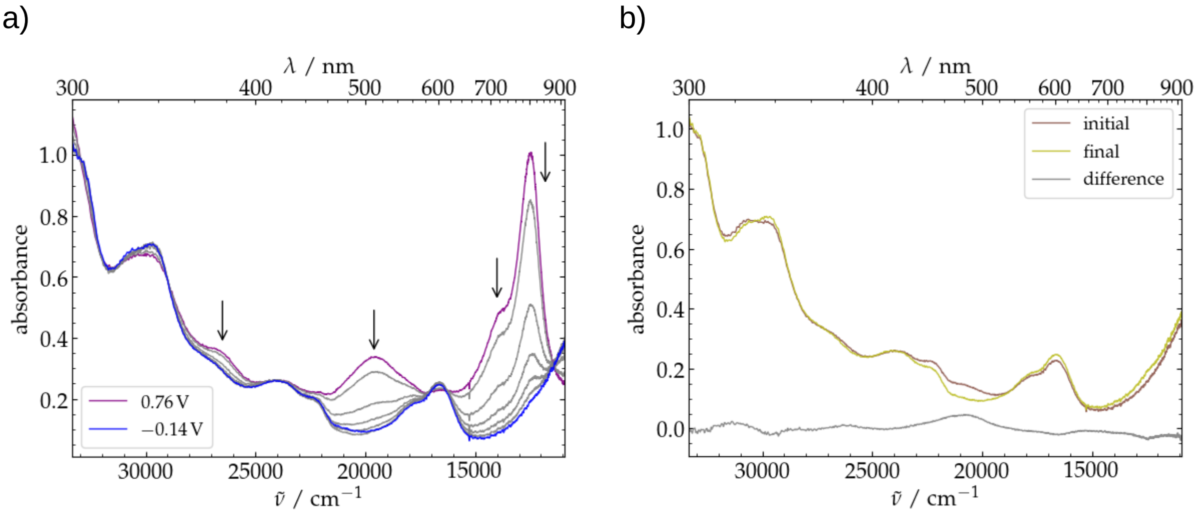

Figure S68. *UV/Vis spectroelectrochemistry of a 1 mM solution of complex* ***2a*** *in* CH_3CN *with 0.1 M* $^{n}Bu_4NPF_6$ *as electrolyte: a) re-reduction at an applied potential of −0.14 V (300 s), of the intermediate obtained by oxidizing* ***2a*** *at −0.14 V for 300 seconds and applying a potential of 0.76 V for additional 300 s (purple spectrum, Figure 7b); b) overlay of the initial spectrum at 300 s with the final spectrum at 900 s obtained after re-reduction.*

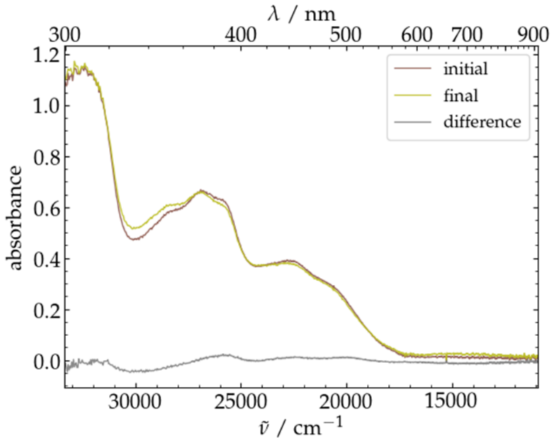

Figure S69. *UV/Vis spectroelectrochemistry of a 1 mM solution of complex* ***2b*** *in THF with 0.2 M* $^{n}Bu_4NPF_6$ *as electrolyte: overlay of the initial spectrum of* ***2b*** *with the final spectrum obtained after re-reduction of the product of the oxidation at −0.13 V vs* $Fc^{+/0}$.

9. Crystallographic Details

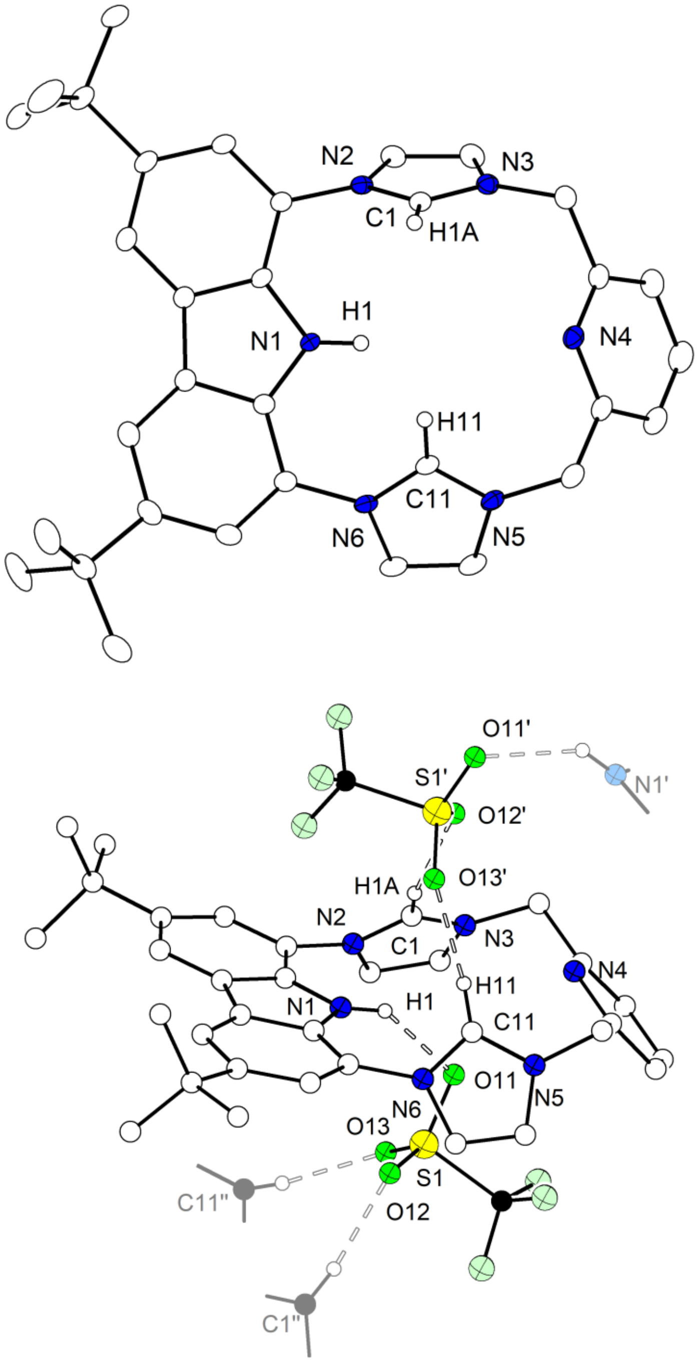

Figure S70. *Top: Plot (30 % probability thermal ellipsoids) of the cationic part of [LH$_3$](OTf)$_2$ (most hydrogen atoms and disorder omitted for clarity); Bottom: Hydrogen bonds in [LH$_3$](OTf)$_2$. Selected bond lengths [Å] and angles [°]: N1···O11 2.8662(16), C1···O12' 2.9596(18), C11···O13' 2.9873(19); N1–H1···O11 142.0, C1–H1···O12' 151.2, C11–H11···O12' 153.9. Symmetry transformations used to generate equivalent atoms: (') 3/2–x, –1/2+y, 3/2–z; ('') 3/2–x, 1/2+y, 3/2–z.*

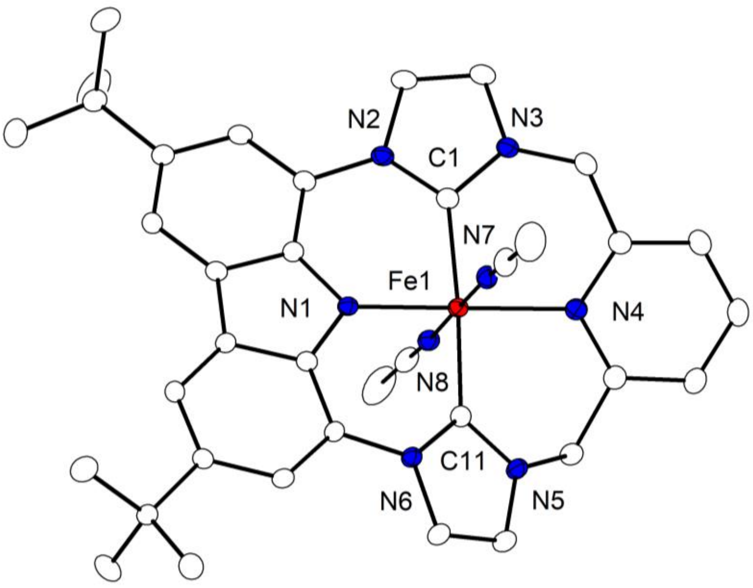

Figure S71. *Plot (30 % probability thermal ellipsoids) of the molecular structure of the cationic part of **2a** (hydrogen atoms omitted for clarity). Selected bond lengths [Å] and angles [°]: Fe1–N1 1.9380(16), Fe1–C1 1.942(2), Fe1–C11 1.941(2), Fe1–N4 2.0713(17), Fe1–N7 1.9338(18), Fe1–N8 1.9377(18); N7–Fe1–N8 178.83(7), N7–Fe1–N1 91.22(7), N8–Fe1–N1 88.49(7), N7–Fe1–C11 88.12(8), N8–Fe1–C11 90.73(8), N1–Fe1–C11 87.90(7), N7–Fe1–C1 91.37(8), N8–Fe1–C1 89.76(8), N1–Fe1–C1 88.01(8), C11–Fe1–C1 175.86(8), N7–Fe1–N4 88.68(7), N8–Fe1–N4 91.61(7), N1–Fe1–N4 179.88(7), C11–Fe1–N4 92.04(7), C1–Fe1–N4 92.06(8), C20–N1–Fe1 127.11(13), C19–N1–Fe1 127.59(13), C5–N4–Fe1 122.09(14), C9–N4–Fe1 121.82(14), C34–N7–Fe1 176.95(19), C36–N8–Fe1 175.78(17).*

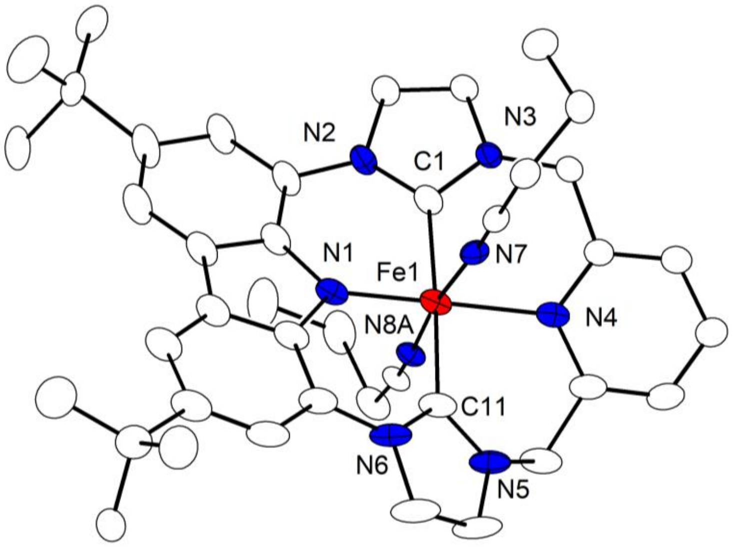

Figure S72. *Plot (30 % probability thermal ellipsoids) of the cationic part of **3a** (hydrogen atoms and disorder omitted for clarity). Selected bond lengths [Å] and angles [°]: Fe1–N1 1.859(3), Fe1–C1 1.956(3), Fe1–C11 1.958(3), Fe1–N4 2.104(3), Fe1–N7 1.937(2), Fe1–N8A 1.950(6); N1–Fe1–N7 92.48(11), N1–Fe1–N8A 95.0(4), N7–Fe1–N8A 172.5(4), N1–Fe1–C1 89.11(13), N7–Fe1–C1 90.45(11), N8A–Fe1–C1 90.2(3), N1–Fe1–C11 88.48(13), N7–Fe1–C11 90.66(11), N8A–Fe1–C11 89.0(3), C1–Fe1–C11 177.39(15), N1–Fe1–N4 179.19(10), N7–Fe1–N4 88.32(10), N8A–Fe1–N4 84.2(4), C1–Fe1–N4 90.89(12), C11–Fe1–N4 91.50(13), C34–N7–Fe1 175.7(2), C38A–N8A–Fe1 174.2(9).*

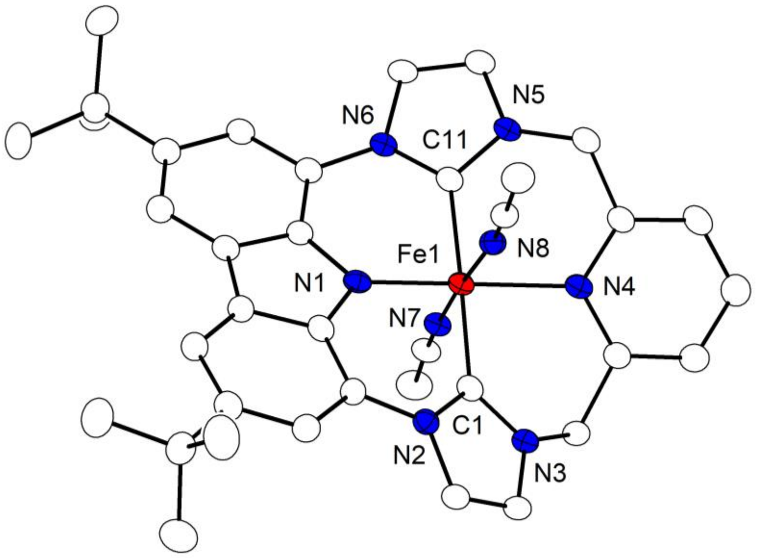

Figure S73. *Plot (30 % probability thermal ellipsoids) of the cationic part of* ***4a*** *(hydrogen atoms omitted for clarity). Selected bond lengths [Å] and angles [°]: Fe1–N1 1.837(3), Fe1–C1 1.942(3), Fe1–C11 1.944(3), Fe1–N4 2.087(3), Fe1–N7 1.917(3), Fe1–N8 1.921(3); N1–Fe1–N7 92.31(11), N1–Fe1–N8 91.91(11), N7–Fe1–N8 175.76(12), N1–Fe1–C1 89.60(12), N7–Fe1–C1 92.44(12), N8–Fe1–C1 87.98(12), N1–Fe1–C11 89.10(12), N7–Fe1–C11 87.50(12), N8–Fe1–C11 92.17(12), C1–Fe1–C11 178.70(14), N1–Fe1–N4 178.90(11), N7–Fe1–N4 86.65(11), N8–Fe1–N4 89.13(11), C1–Fe1–N4 90.77(12), C11–Fe1–N4 90.52(12), C34–N7–Fe1 177.2(3), C36–N8–Fe1 173.6(3).*

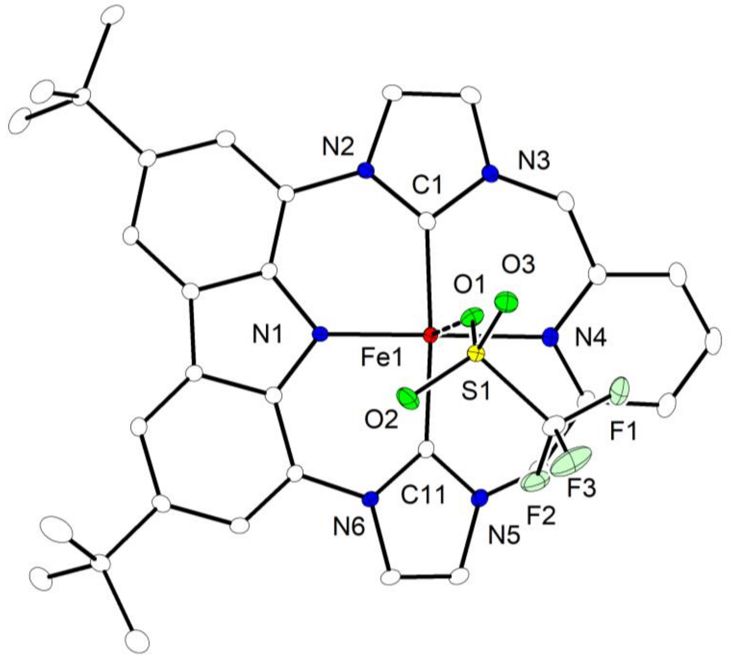

Figure S74. *Plot (30 % probability thermal ellipsoids) of the molecular structure of* ***2b*** *(hydrogen atoms omitted for clarity). Selected bond lengths [Å] and angles [°]: Fe1–N1 1.8994(12), Fe1–C1 1.9431(15), Fe1–C11 1.9443(15), Fe1–N4 2.1003(13), Fe1–O1 2.3221(11); N1–Fe1–C1 88.25(6), N1–Fe1–C11 88.40(6), C1–Fe1–C11 173.82(6), N1–Fe1–N4 173.64(5), C1–Fe1–N4 91.62(6), C11–Fe1–N4 91.12(6), N1–Fe1–O1 100.24(5), C1–Fe1–O1 87.50(5), C11–Fe1–O1 98.22(5), N4–Fe1–O1 86.10(4).*

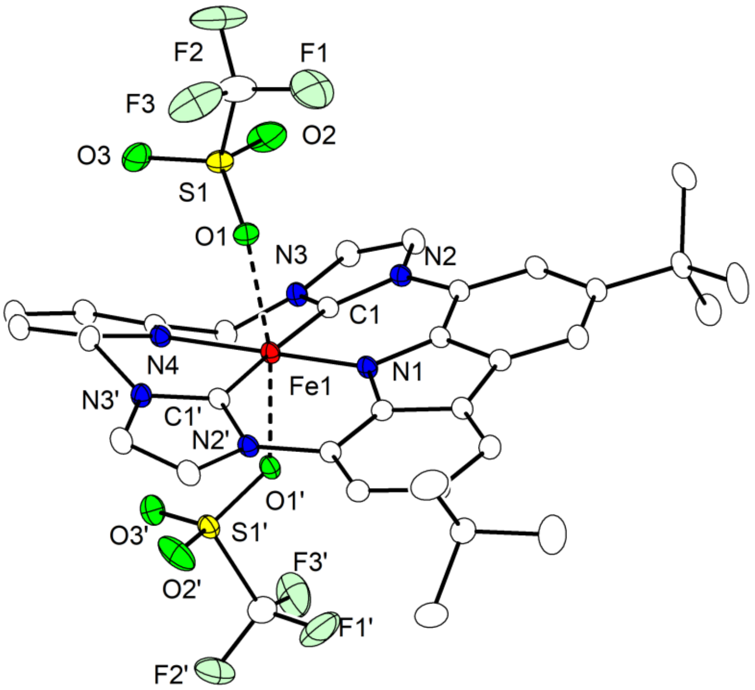

Figure S75. *Plot (30 % probability thermal ellipsoids) of the molecular structure of* ***3b*** *(hydrogen atoms omitted for clarity). Selected bond lengths [Å] and angles [°]: Fe1–N1 1.884(3), Fe1–C1 1.976(2), Fe1–N4 2.133(3), Fe1–O1 2.2261(17); N1–Fe1–C1 88.22(7), C1–Fe1–C1' 176.45(14), N1–Fe1–N4 180.0, C1–Fe1–N4 91.78(7), N1–Fe1–O1' 96.19(5), C1–Fe1–O1' 88.34(8), N1–Fe1–O1 96.19(5), C1–Fe1–O1 92.04(8), N4–Fe1–O1 83.81(5), O1'–Fe1–O1 167.62(9). Symmetry transformation used to generate equivalent atoms: (') 1–x, y, 3/2–z.*

Table S1. *Crystal data and refinement details for [LH$_3$](OTf)$_2$, **2b**, and **2a**.*

**compound**	**[LH$_3$](OTf)$_2$ (mm_l)**	**2b (mm2b2_20)**	**2a (mm94)**
empirical formula	$C_{39}H_{42}F_6N_8O_6S_2$	$C_{40}H_{37}F_5FeN_6O_3S$	$C_{46}H_{59}F_3FeN_8O_5S$
moiety formula	$C_{33}H_{36}N_6^{2+}$, 2($CF_3O_3S^-$), 2(C_2H_3N)	$C_{34}H_{33}F_3FeN_6O_3S$, $C_6H_4F_2$	$C_{37}H_{39}FeN_8^+$, $CF_3O_3S^-$, 2($C_4H_{10}O$)
formula weight	896.92	832.66	948.92
T [K]	100(2)	120(2)	133(2)
crystal size [mm³]	0.422 x 0.335 x 0.318	0.372 x 0.363 x 0.270	0.360 x 0.190 x 0.160
crystal system	monoclinic	monoclinic	monoclinic
space group	$P2_1/n$ (No. 14)	$P2_1/c$ (No. 14)	$P2_1/c$ (No. 14)
a [Å]	17.6175(4)	15.8121(5)	22.3544(7)
b [Å]	12.6128(3)	16.6628(5)	11.3554(2)
c [Å]	19.4018(6)	14.9904(5)	19.1330(6)
β [°]	96.791(1)	110.823(1)	100.480(2)
V [Å³]	4280.95(19)	3691.6(2)	4775.8(2)
Z	4	4	4
ρ [g·cm^{-3}]	1.392	1.498	1.320
F(000)	1864	1720	2000
μ [mm^{-1}]	0.206	0.539	0.424
T_{min} / T_{max}	0.91 / 0.94$^{\$}$	0.81 / 0.87$^{\$}$	0.8077 / 0.9246*
θ–range [°]	2.318 – 27.895	1.899 – 27.909	1.853 – 26.905
hkl–range	–21 to 23, ±16, ±25	±20, ±21, ±19	±28, –14 to 12, ±24
measured refl.	113224	100758	66852
unique refl. [R_{int}]	10227 [0.0329]	8819 [0.0455]	10176 [0.0494]
observed refl. ($I > 2\sigma(I)$)	8975	7702	7774
data / restr. / param.	10227 / 183 / 659	8819 / 0 / 511	10176 / 171 / 689
goodness–of–fit (F^2)	1.048	1.041	1.036
$R1$, $wR2$ ($I > 2\sigma(I)$)	0.0417 / 0.1116	0.0318 / 0.0721	0.0405 / 0.0952
$R1$, $wR2$ (all data)	0.0486 / 0.1192	0.0396 / 0.0768	0.0617 / 0.1067
res. el. dens. [e·Å^{-3}]	–0.529 / 0.472	–0.394 / 0.523	–0.567 / 0.401

*) X-RED. $^{\$}$) SADABS.

Table S2. *Crystal data and refinement details for* ***3b****,* ***4a****, and* ***3a****.*

**compound**	**3b (mm340)**	**4a (mm335)**	**3a (mm-ir20)**
empirical formula	$C_{45}H_{55}F_6FeN_6O_9S_2$	$C_{52}H_{65}F_9FeN_{10}O_{11}S_3$	$C_{47}H_{54}F_6FeN_9O_6S_2$
moiety formula	$C_{35}H_{33}F_6FeN_6O_6S_2$, $C_4H_{10}O$, 2(C_3H_6O)	$C_{37}H_{39}FeN_8^{3+}$, 3($CF_3O_3S^-$), 2($C_4H_{10}O$), 2(C_2H_3N)	$C_{41}H_{47}FeN_8^{2+}$, 2($CF_3O_3S^-$), C_4H_7N
formula weight	1057.92	1329.17	1074.96
T [K]	133(2)	133(2)	133(2)
crystal size [mm³]	0.500 x 0.240 x 0.200	0.480 x 0.340 x 0.180	0.500 x 0.140 x 0.080
crystal system	monoclinic	monoclinic	triclinic
space group	$C2/c$ (No. 15)	$P2_1/c$ (No. 14)	P–1 (No. 2)
a [Å]	13.4731(4)	17.1718(4)	9.7933(4)
b [Å]	27.1045(10)	20.3275(4)	11.1501(5)
c [Å]	14.2582(4)	18.5181(5)	23.3424(12)
α [°]	90	90	87.425(4)
β [°]	102.227(2)	105.770(2)	80.913(4)
γ [°]	90	90	77.856(4)
V [Å³]	5088.7(3)	6220.6(3)	2460.4(2)
Z	4	4	2
ρ [g·cm⁻³]	1.381	1.419	1.451
F(000)	2204	2760	1118
μ [mm⁻¹]	0.458	0.433	0.472
T_{min} / T_{max}	0.5707 / 0.8708*	0.7561 / 0.9344*	0.7746 / 0.9256*
θ–range [°]	1.503 – 26.928	1.232 – 25.820	1.767 – 25.712
hkl–range	±17, ±34, –18 to 17	–21 to 20, –22 to 24, ±22	±11, ±13, ±28
measured refl.	35310	76501	28152
unique refl. [R_{int}]	5412 [0.0728]	11802 [0.0582]	9229 [0.0408]
observed refl. ($I > 2\sigma(I)$)	4509	9571	6730
data / restr. / param.	5412 / 0 / 320	11802 / 226 / 910	9229 / 331 / 833
goodness–of–fit (F^2)	1.066	1.138	1.029
$R1$, $wR2$ ($I > 2\sigma(I)$)	0.0523 / 0.1349	0.0580 / 0.1418	0.0531 / 0.1268
$R1$, $wR2$ (all data)	0.0638 / 0.1440	0.0748 / 0.1540	0.0797 / 0.1413
res. el. dens. [e·Å⁻³]	–0.369 / 0.775	–0.436 / 0.885	–0.417 / 0.773

*) X-RED. $) SADABS.

Table S3. *Selected bond lengths [Å] and angles [°].*

	**2b**	**2a**	**3b**	**4a**	**3a**
Fe–N^{cbz}	1.8994(12)	1.9380(16)	1.884(3)	1.837(3)	1.859(3)
Fe–N^{py}	2.1003(13)	2.0713(17)	2.133(3)	2.087(3)	2.104(3)
Fe–C	1.9431(15) / 1.9443(15)	1.941(2) / 1.942(2)	1.976(2)	1.942(3) / 1.944(3)	1.956(3) / 1.958(3)
Fe–$N^{nitrile}$	2.1003(13)	1.9338(18) / 1.9377(18)	–	1.917(3) / 1.921(3)	1.902(10) – 1.950(6)*
Fe–O^{OTf}	2.3221(11)	–	2.2261(17)	–	–
N^{py}–Fe–N^{cbz}	173.64(5)	179.88(7)	180.0	178.90(11)	179.19(10)
C–Fe–C	173.82(6)	175.86(8)	176.45(14)	178.70(14)	177.39(15)
$N^{nitrile}$ –Fe–$N^{nitrile}$	–	178.83(7)	–	175.76(12)	171.0(6) / 172.5(4)
O^{OTf} –Fe–O^{OTf}	–	–	167.62(9)	–	–

*) one nitrile is disordered about two positions

10. DFT Calculations

Sample input files for the calculations:

Geometry optimisations

```
! UKS BP86 def2-TZVP RI def2/J D3BJ TightSCF Opt NumFreq
* xyzfile <charge> <multiplicity> <input coordinates>.xyz
```

Single-point calculations

```
! UKS B3LYP def2-TZVP RIJCOSX def2/J D3BJ UCO UNO TightSCF
* xyzfile <charge> <multiplicity> <optimised coordinates>.xyz
```

Calculation of MB parameters

```
! UKS B3LYP def2-TZVP NoRI D3BJ TightSCF
%basis NewGTO 26 "CP(PPP)" end
end
* xyzfile <charge> <multiplicity> <optimised coordinates>.xyz
%eprnmr nuclei = all Fe {rho, fgrad}
end
```

TD-DFT calculations

```
! UKS BP86 def2-TZVP RI def2/J D3BJ TightSCF TightOpt CPCM(<solvent>)
* xyzfile <charge> <multiplicity> <optimised coordinates>.xyz
```

```
! UKS B3LYP def2-TZVP RIJCOSX def2/J D3BJ TightSCF CPCM(<solvent>)
%tddft
  MaxDim 10
  NRoots 80
  DoNTO True
end
* xyzfile <charge> <multiplicity> <CPCM-optimised coordinates>.xyz
```

10.1. Complex **2a**

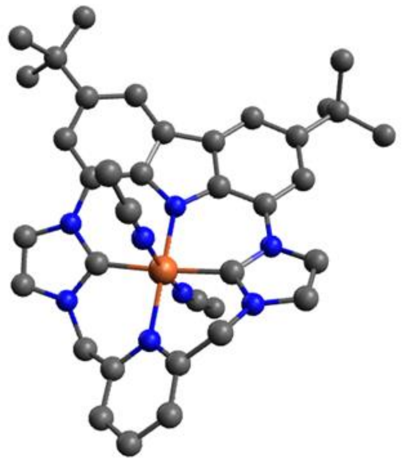

Figure S76. *Visualization of the optimized geometry of the cation of complex* ***2a*** *based on DFT calculation at the BP86 level (grey: C, blue: N, orange: Fe; hydrogen atoms omitted for clarity).*

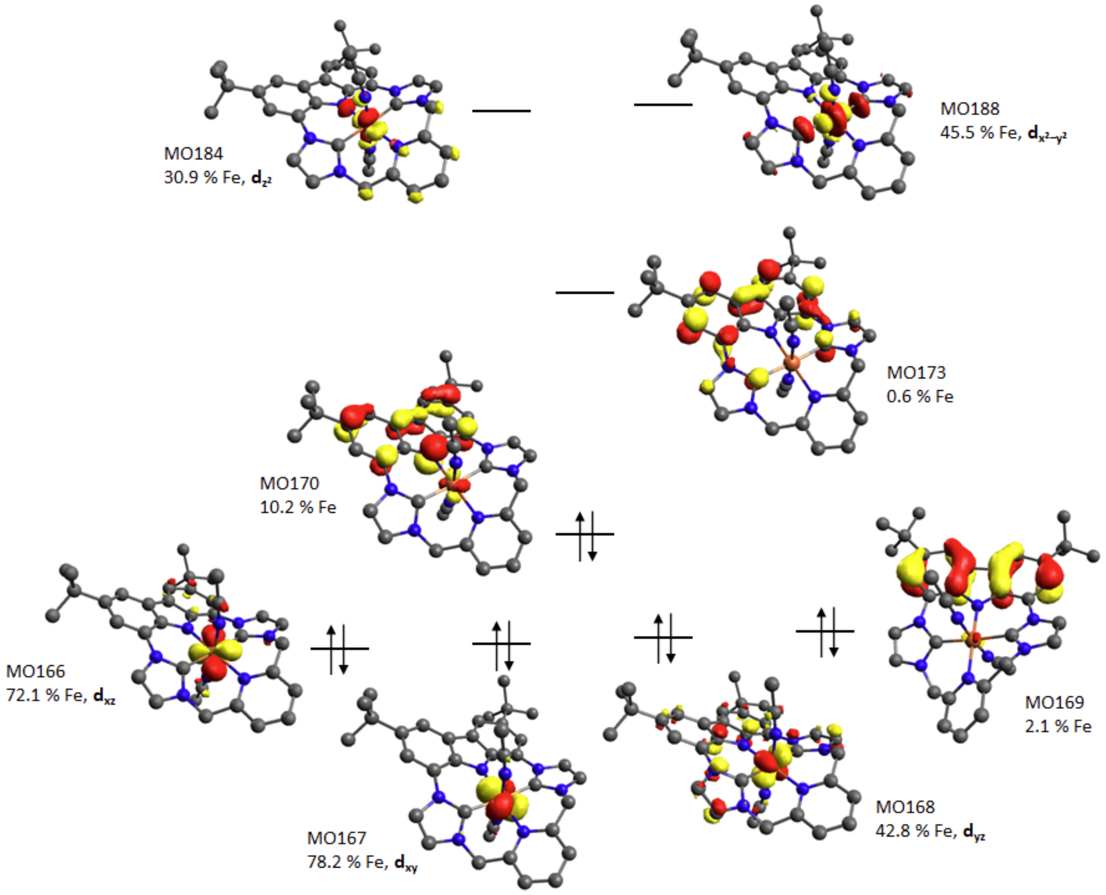

Figure S77. *Schematic molecular orbital diagram of* ***2a*** *represented by quasi-restricted orbitals (isovalue 0.05) at the B3LYP level. Iron contribution and atom orbital assignments are based on Löwdin reduced orbital populations per MO.*

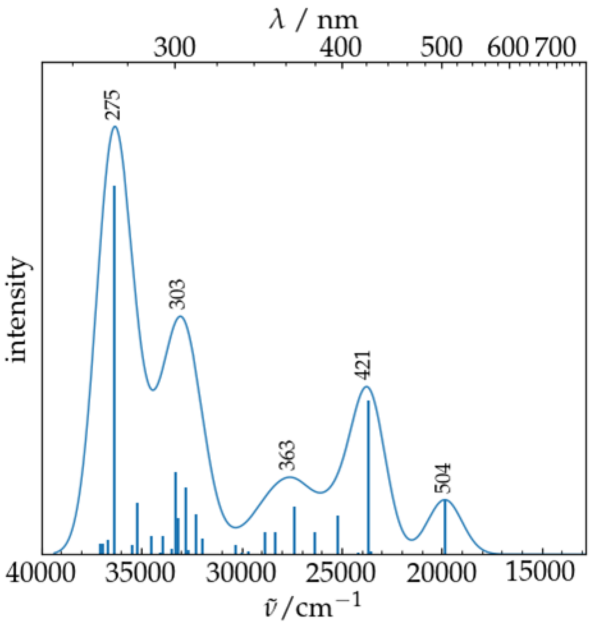

Figure S78. *Calculated absorption spectrum of the cation of complex* ***2a*** *in CH_3CN at the B3LYP level.*

Table S4. *Selected TD-DFT (B3LYP) calculated energies, oscillator strengths and major contributions of the main electronic transitions of complex* ***2a****.*

States	Energy (cm^{-1})	Wavelength (nm)	Osc. Strength	Major Contributions
5	19833.7	504.2	0.045360063	HOMO → LUMO (96 %)
12	23661.8	422.6	0.129987940	HOMO → LUMO+1 (94 %)

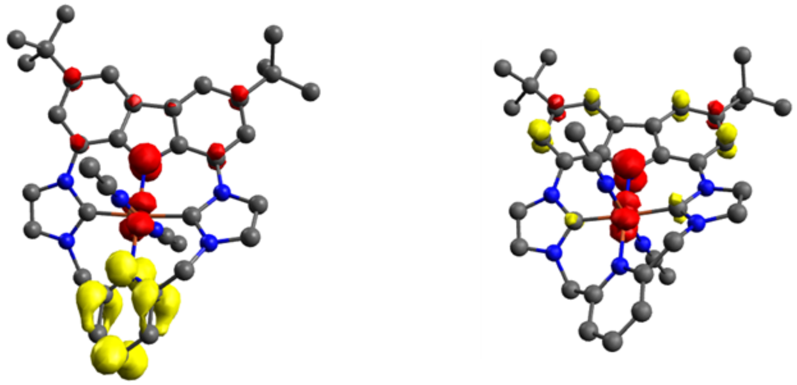

Figure S79. *Calculated TD-DFT difference density of complex* ***2a*** *(yellow/red indicates gain/loss of electron density) for states 5 (504 nm) and 12 (423 nm).*

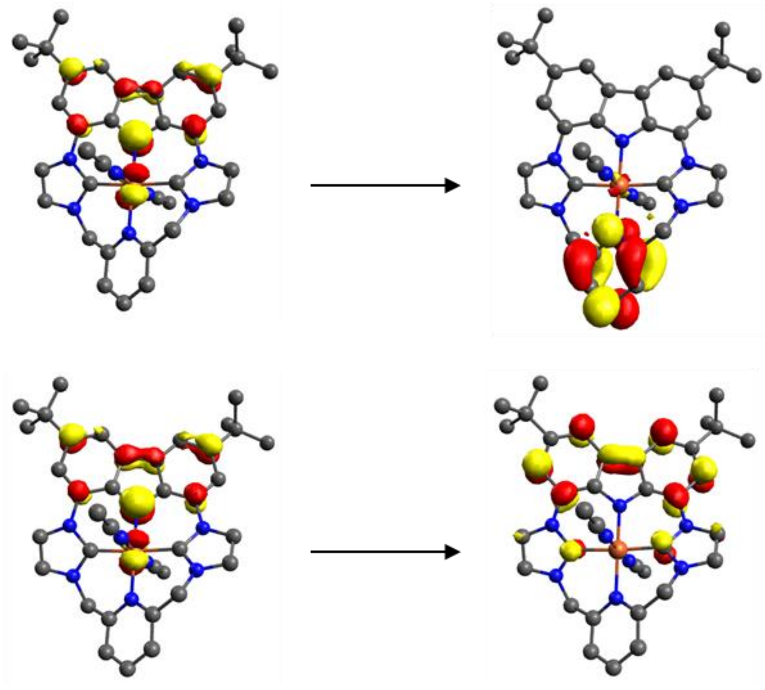

Figure S80. *Natural transition orbitals that mainly contribute to states 5 and 12 for complex* ***2a****.*

10.2. Complex **2b**

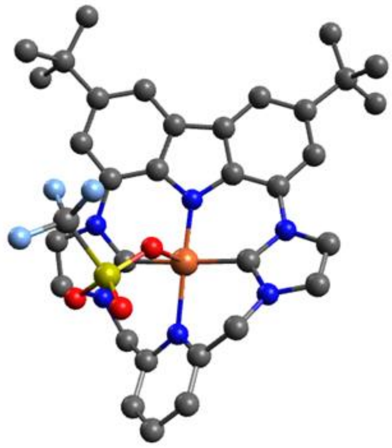

Figure S81. *Visualization of the optimized geometry of complex* ***2b*** *based on DFT calculation at the BP86 level (grey: C, blue: N, red: O, yellow: S, light blue: F, orange: Fe; hydrogen atoms omitted for clarity).*

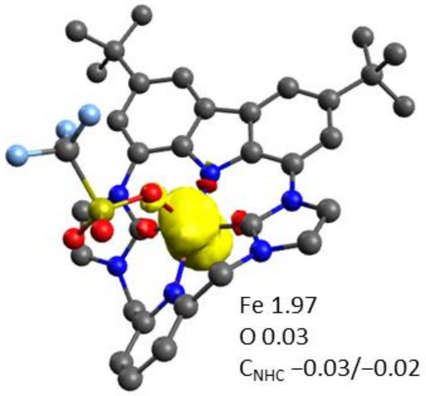

Figure S82. *DFT calculated spin density plot (isovalue 0.005) for complex* ***2b*** *at the B3LYP level. Spin distribution according to Löwdin population analysis.*

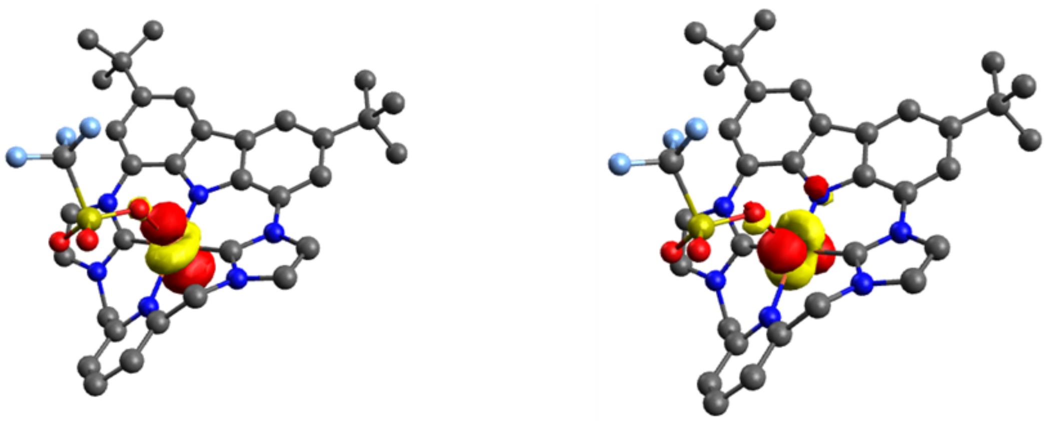

Figure S83. *Plots of the magnetic orbitals (MO 185/186a, isovalue 0.05) of complex* ***2b*** *at the B3LYP level.*

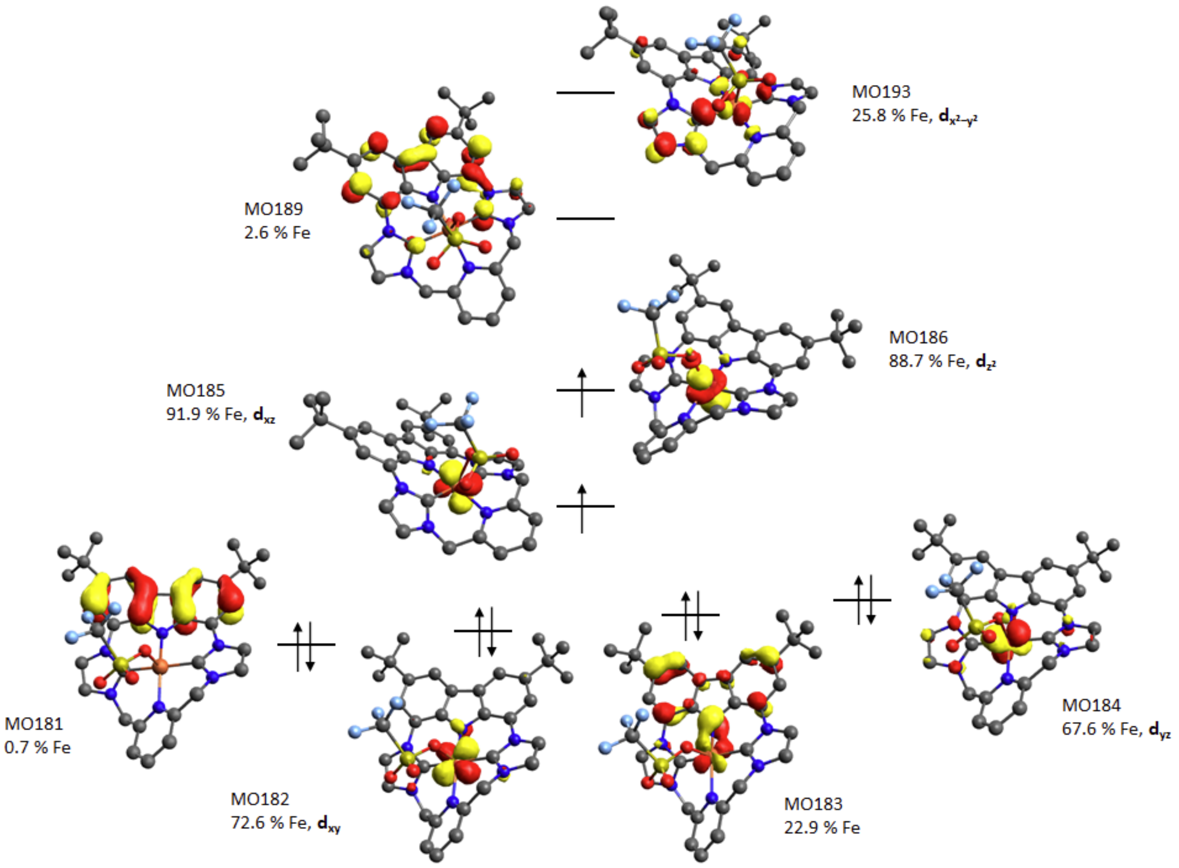

Figure S84. *Schematic molecular orbital diagram of* ***2b*** *represented by quasi-restricted orbitals (isovalue 0.05) at the B3LYP level. Iron contribution and atom orbital assignments are based on Löwdin reduced orbital populations per MO.*

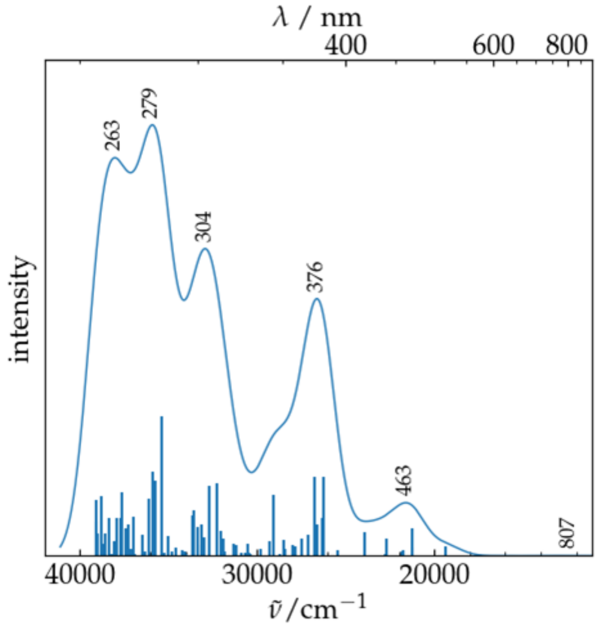

Figure S85. *Calculated absorption spectrum of the cation of complex* ***2b*** *in THF at the B3LYP level.*

Table S5. *Selected TD-DFT (B3LYP) calculated energies, oscillator strengths and major contributions of the main electronic transitions of complex* ***2b****.*

States	Energy (cm^{-1})	Wavelength (nm)	Osc. Strength	Major Contributions
7	21280.6	469.9	0.021268962	HOMO → LUMO (78 %)<br>HOMO → LUMO+1 (8 %)
10	22666.5	441.2	0.012972085	HOMO−2 → LUMO (53 %)<br>HOMO−1 → LUMO+3 (8 %)<br>HOMO−2 → LUMO+3 (8 %)
12	23964.7	417.3	0.017519645	HOMO → LUMO+1 (56 %)<br>HOMO → LUMO+4 (10 %)

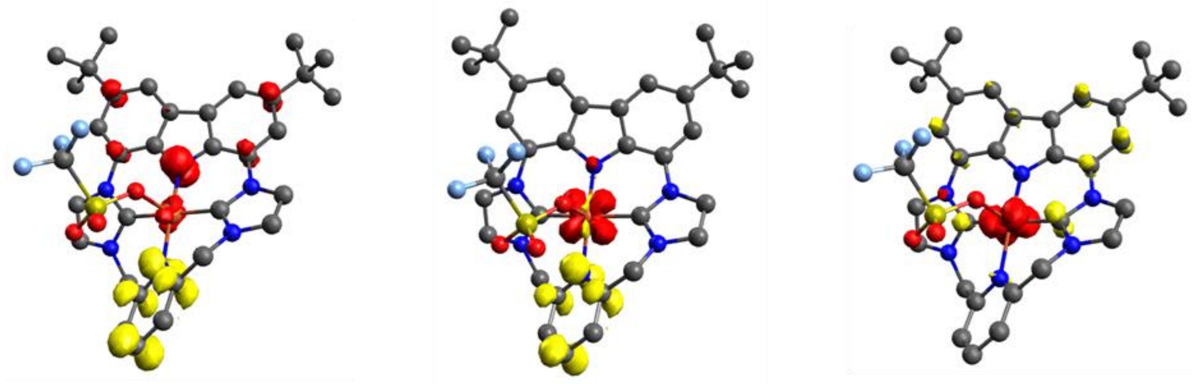

Figure S86. *Calculated TD-DFT difference density of complex* ***2b*** *(yellow/red indicates gain/loss of electron density) for states 7 (470 nm), 10 (441 nm) and 12 (417 nm).*

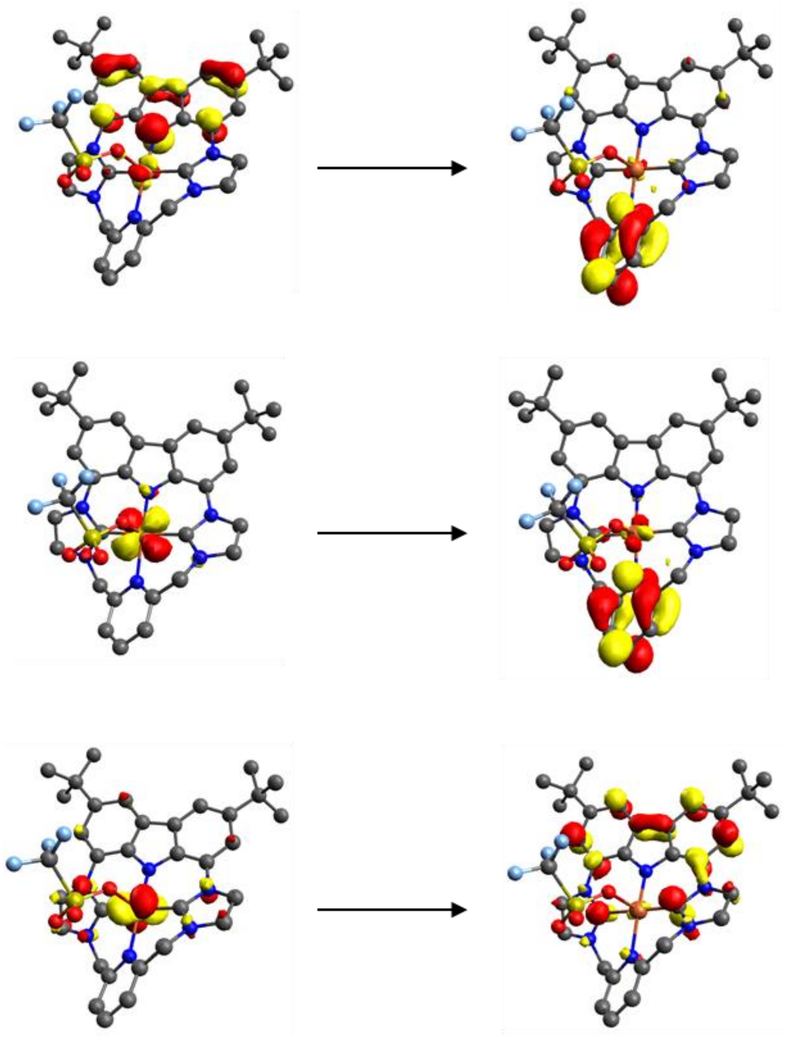

Figure S87. *Natural transition orbitals that mainly contribute to states 7, 10 and 12 for complex* ***2b***.

10.3. Complex **3a**

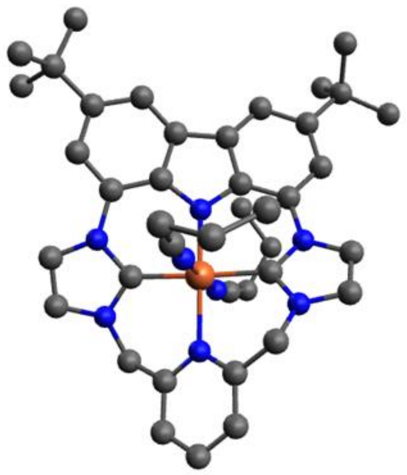

Figure S88. *Visualization of the optimized geometry of the cation of complex* ***3a*** *based on DFT calculation at the BP86 level (grey: C, blue: N, orange: Fe; hydrogen atoms omitted for clarity).*

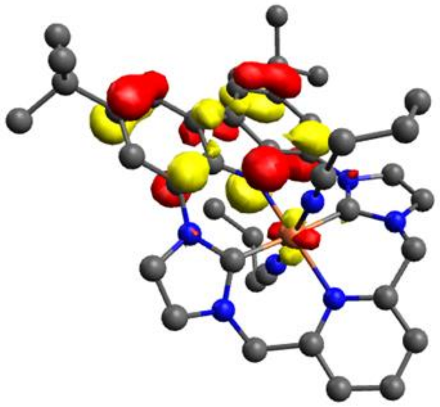

Figure S89. *Plot of the magnetic orbital (MO 186a, isovalue 0.05) of complex **3a** at the B3LYP level.*

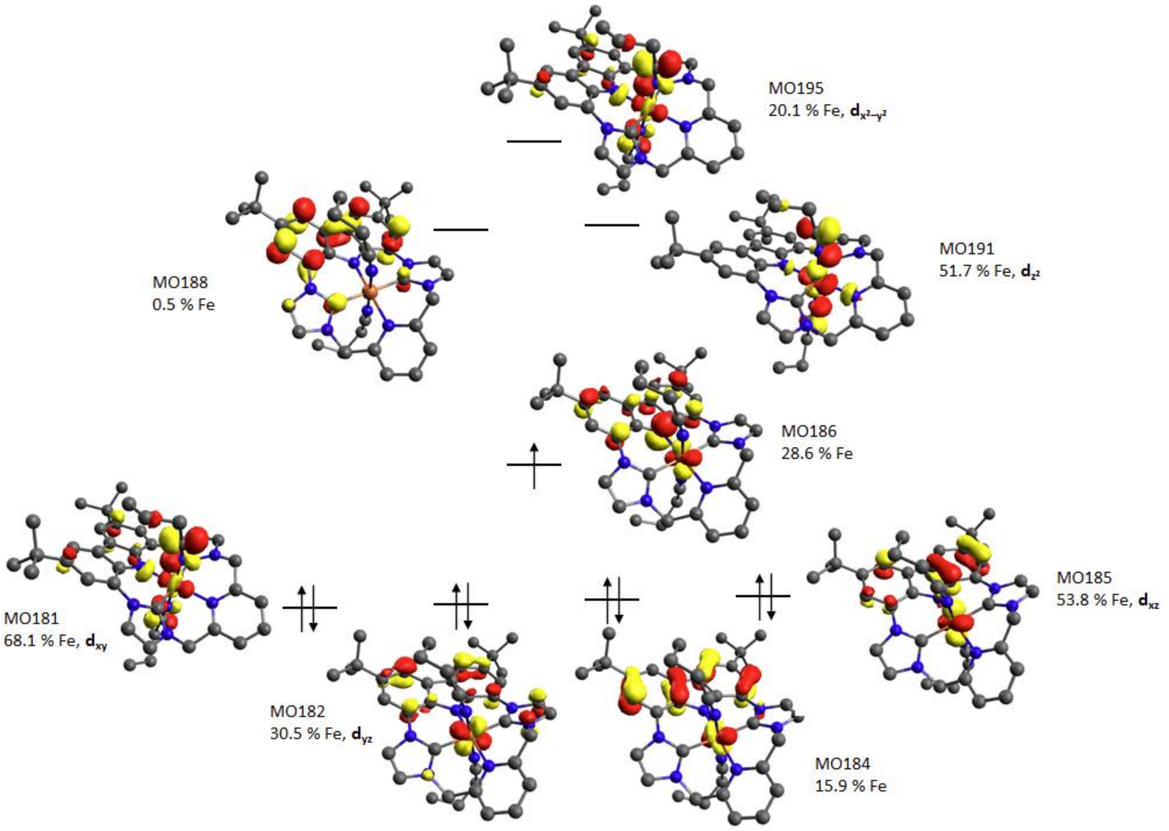

Figure S90. *Schematic molecular orbital diagram of **3a** represented by quasi-restricted orbitals (isovalue 0.05) at the B3LYP level. Iron contribution and atom orbital assignments are based on Löwdin reduced orbital populations per MO.*

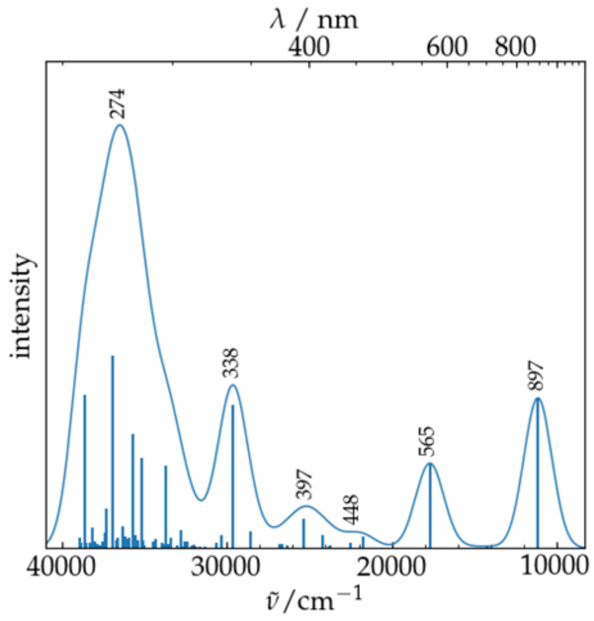

Figure S91. *Calculated absorption spectrum of the cation of complex **3a** in CH_3CN at the B3LYP level.*

Table S6. *Selected TD-DFT (B3LYP) calculated energies, oscillator strengths and major contributions of the main electronic transitions of complex **3a**.*

States	Energy (cm^{-1})	Wavelength (nm)	Osc. Strength	Major Contributions
3	11148.1	897.0	0.134380045	HOMO → LUMO (88 %)
7	17693.1	565.2	0.075940500	HOMO−3 → LUMO (51 %)<br>HOMO−2 → LUMO (25 %)<br>HOMO−1 → LUMO (13 %)

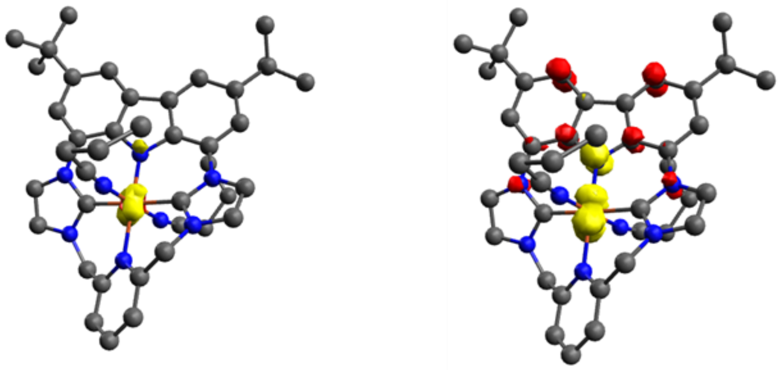

Figure S92. *Calculated TD-DFT difference density of complex **3a** (yellow/red indicates gain/loss of electron density) for states 3 (897 nm) and 7 (565 nm).*

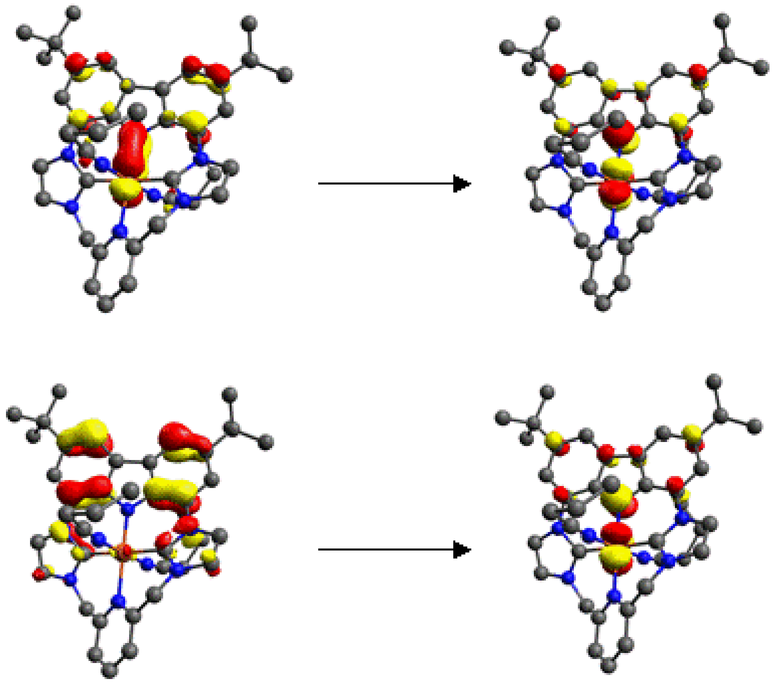

Figure S93. *Natural transition orbitals that mainly contribute to states 3 and 7 for complex* ***3a****.*

10.4. Complex **3b**

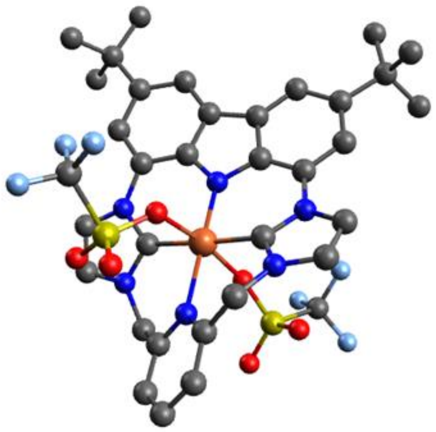

Figure S94. *Visualization of the optimized geometry of complex* ***3b*** *based on DFT calculation at the BP86 level (grey: C, blue: N, red: O, yellow: S, light blue: F, orange: Fe; hydrogen atoms omitted for clarity).*

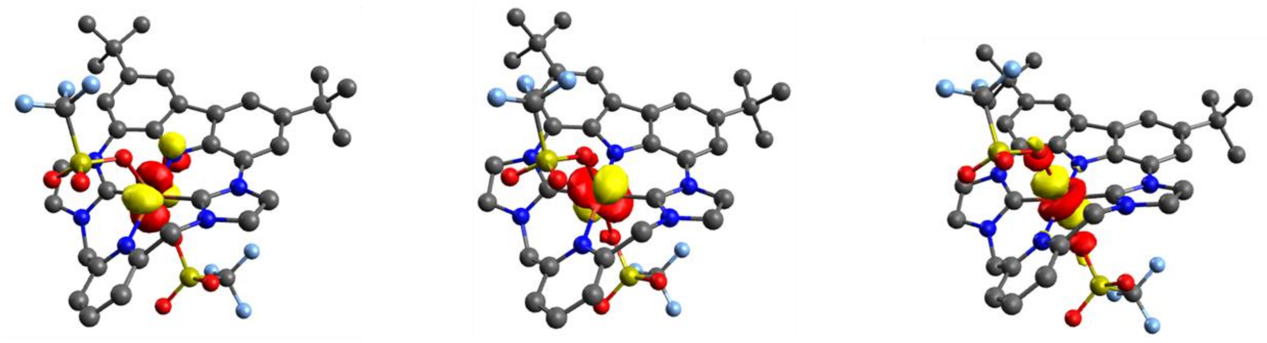

Figure S95. *Plots of the magnetic orbitals (MO 221/222/223a, isovalue 0.05) of complex* ***3b*** *at the B3LYP level.*

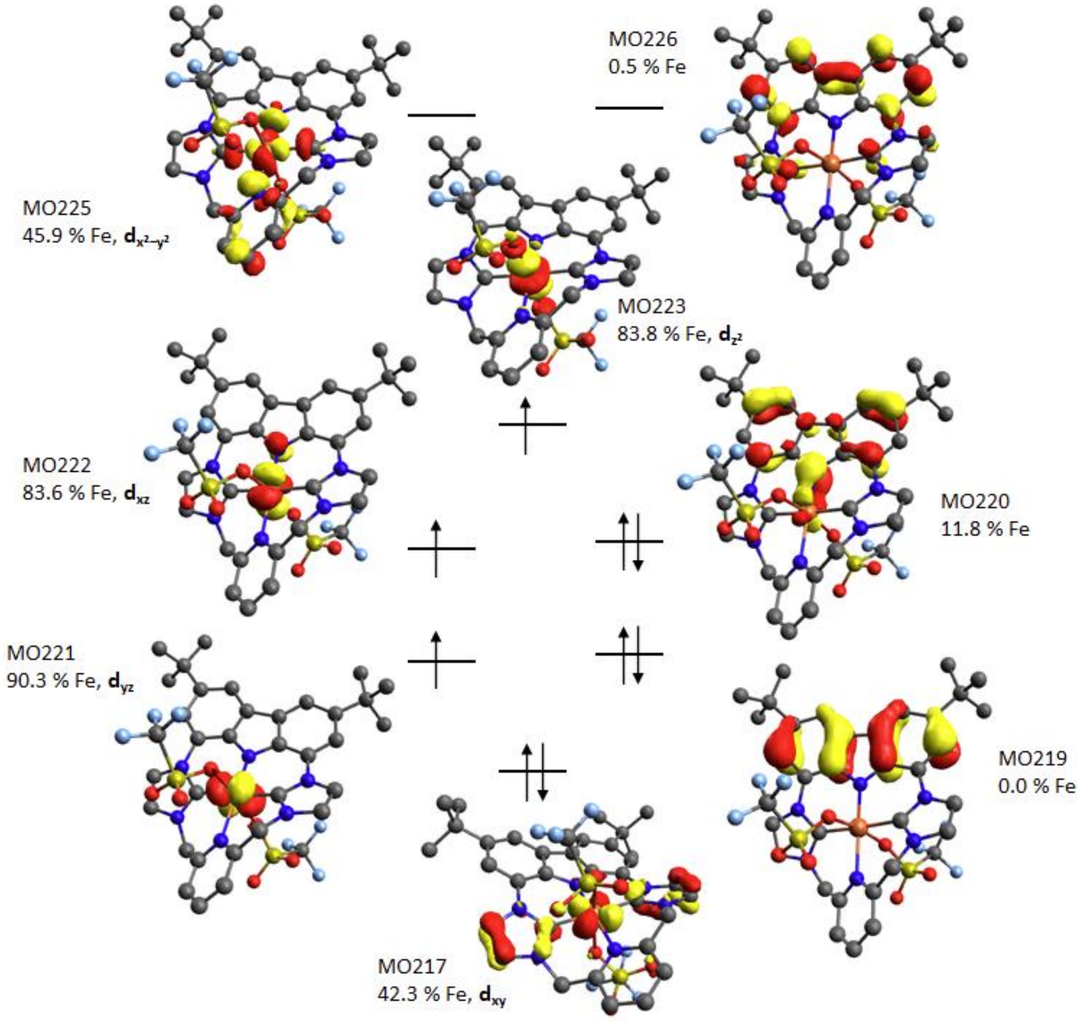

Figure S96. *Schematic molecular orbital diagram of* ***3b*** *represented by quasi-restricted orbitals (isovalue 0.05) at the B3LYP level. Iron contribution and atom orbital assignments are based on Löwdin reduced orbital populations per MO.*

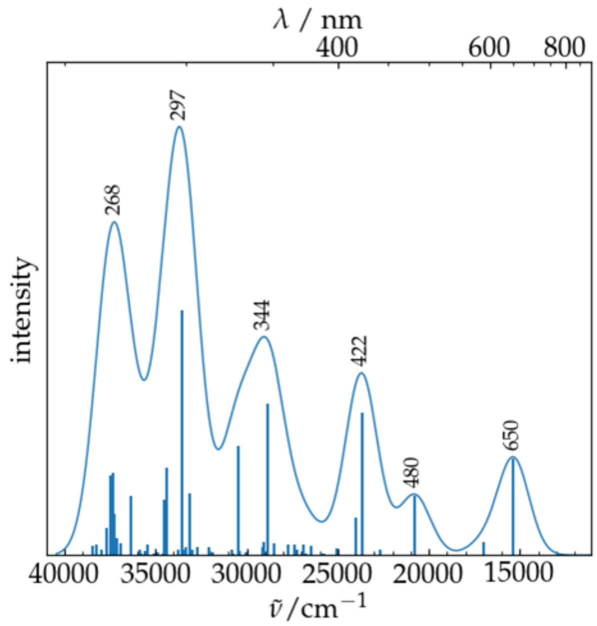

Figure S97. *Calculated absorption spectrum of complex* ***3b*** *in acetone at the B3LYP level.*

Table S7. *Selected TD-DFT (B3LYP) calculated energies, oscillator strengths and major contributions of the main electronic transitions of complex* ***3b****.*

States	Energy (cm^{-1})	Wavelength (nm)	Osc. Strength	Major Contributions
5	15350.8	651.4	0.069635168	HOMO → LUMO+1 (82 %)
10	20792.9	480.9	0.042697452	HOMO−2 → LUMO (87 %)
12	23623.4	423.3	0.103743226	HOMO−3 → LUMO (53 %)<br>HOMO−4 → LUMO (26 %)

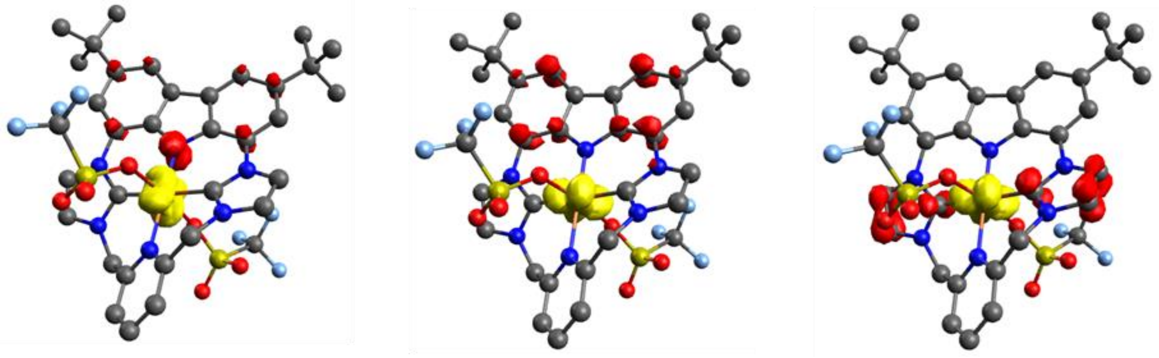

Figure S98. *Calculated TD-DFT difference density of complex* ***3b*** *(yellow/red indicates gain/loss of electron density) for states 5 (651 nm), 10 (481 nm) and 12 (423 nm).*

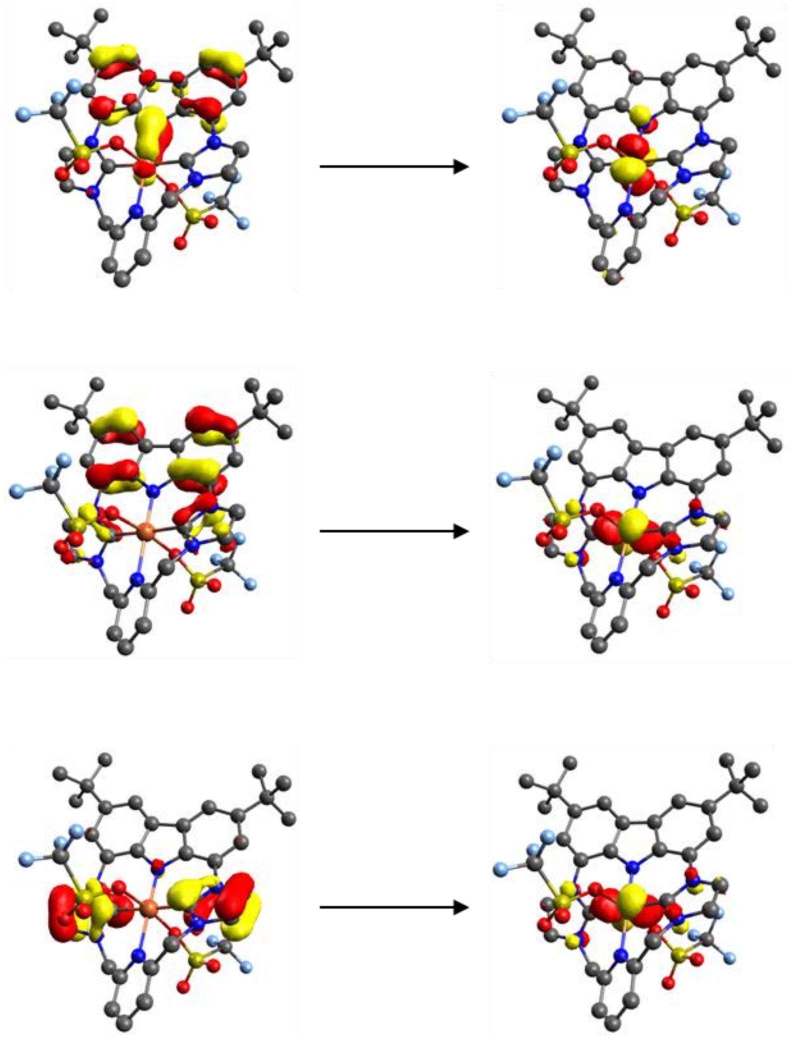

Figure S99. *Natural transition orbitals that mainly contribute to states 5, 10 and 12 for complex* ***3b****.*

10.5. Complex **4a**

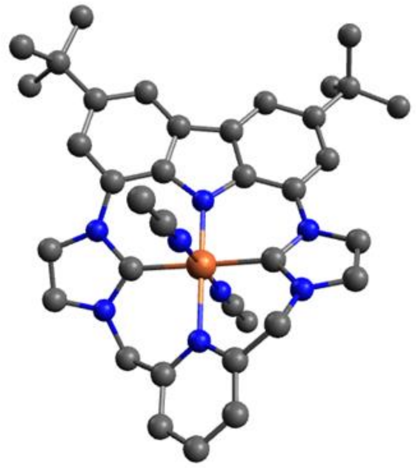

Figure S100. *Visualization of the optimized geometry of the cation of complex* ***4a*** *based on DFT calculation at the BP86 level (grey: C, blue: N, orange: Fe; hydrogen atoms omitted for clarity).*

Table S8. *Electronic energies and multiplicity of different ground states of* ***4a*** *calculated at the B3LYP level with variable fractions of Hartree–Fock exchange (HFX).*

**HFX [%]**	**10**		**20** (B3LYP)		**30**	
Multiplicity	E [H]	ΔE [kcal mol^{-1}]	E [H]	ΔE [kcal mol^{-1}]	E [H]	ΔE [kcal mol^{-1}]
Singlet	–3131.25012	0.1	–3134.01401	1.4	–3136.79695	4.1
Triplet	–3131.23571	9.1	–3134.00683	5.9	–3136.79803	3.5
Singlet (BS)	–3131.25024	0	–3134.01622	0	–3136.80354	0

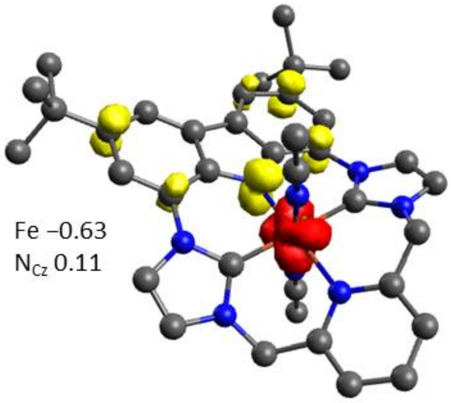

Figure S101. *DFT calculated spin density plot (isovalue 0.005) for complex* ***4a*** *at the B3LYP level. Spin distribution according to Löwdin population analysis.*

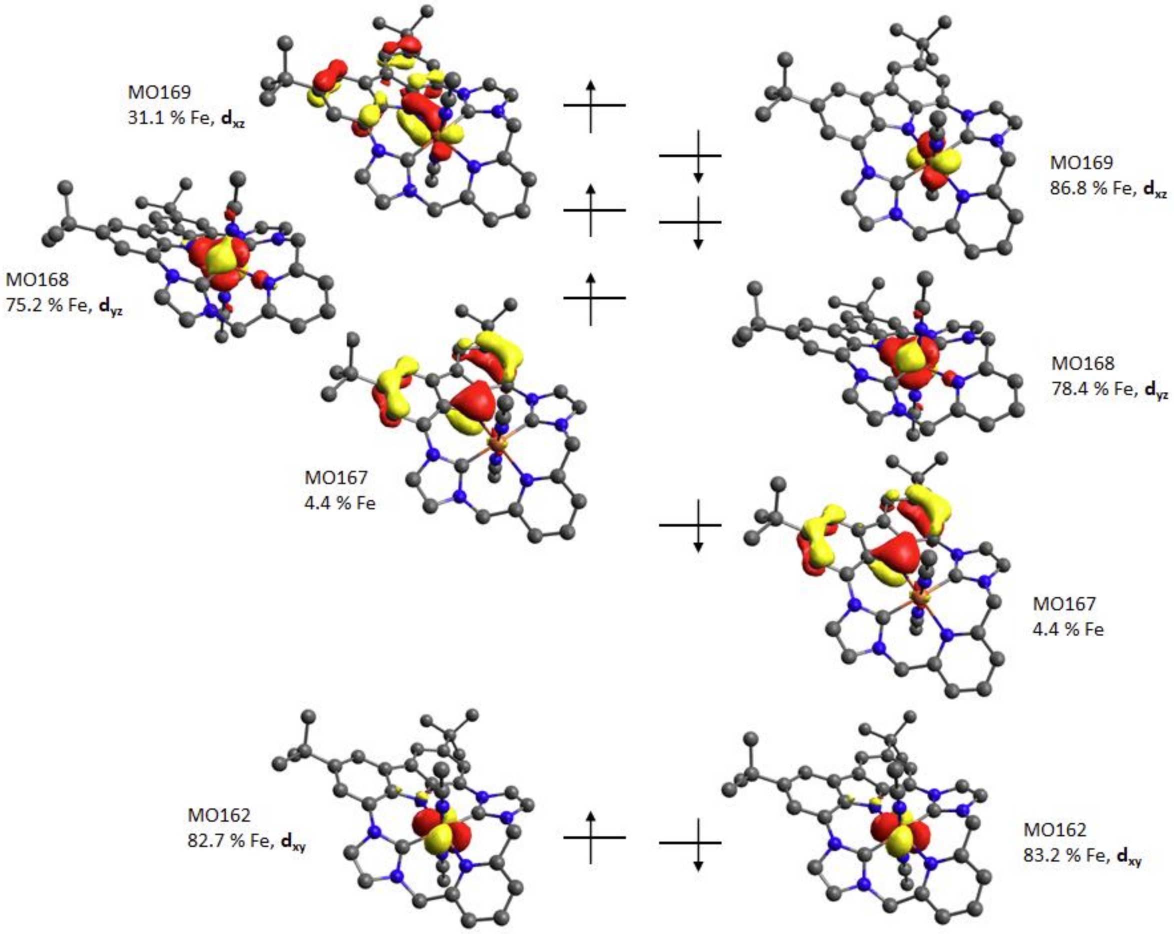

Figure S102. *Schematic molecular orbital diagram of* ***4a*** *represented by unrestricted corresponding orbitals (isovalue 0.05) at the B3LYP level. Iron contribution and atom orbital assignments are based on Löwdin reduced orbital populations per MO.*

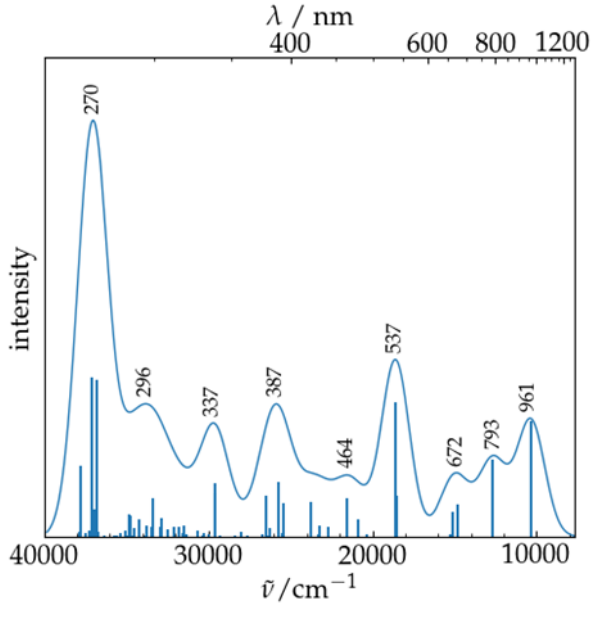

Figure S103. *Calculated absorption spectrum of the cation of complex* ***4a*** *in CH_3CN at the B3LYP level.*

Table S9. *Selected TD-DFT (B3LYP) calculated energies, oscillator strengths and major contributions of the main electronic transitions of complex* ***4a****.*

States	Energy (cm^{-1})	Wavelength (nm)	Osc. Strength	Major Contributions
4	10360.1	965.2	0.103861573	HOMO → LUMO (87 %)
5	12670.4	789.2	0.068497756	HOMO → LUMO (85 %)
6	14835.2	674.1	0.029493884	HOMO−2 → LUMO (64 %)<br>HOMO−3 → LUMO (26 %)
8	15107.4	661.9	0.022746423	HOMO−3 → LUMO (64 %)<br>HOMO−2 → LUMO (27 %)
12	18618.2	537.1	0.120462968	HOMO−4 → LUMO (23 %)<br>HOMO−2 → LUMO (21 %)<br>HOMO−1 → LUMO (18 %)<br>HOMO−5 → LUMO (17 %)

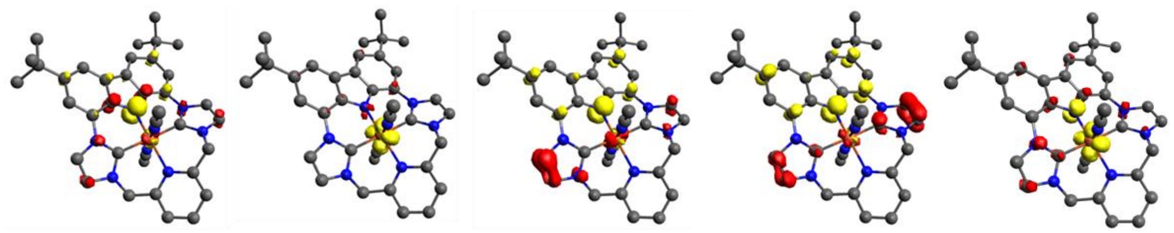

Figure S104. *Calculated TD-DFT difference density of complex* ***4a*** *(yellow/red indicates gain/loss of electron density) for states 4 (965 nm), 5 (789 nm), 6 (674 nm), 8 (662 nm) and 12 (537 nm).*

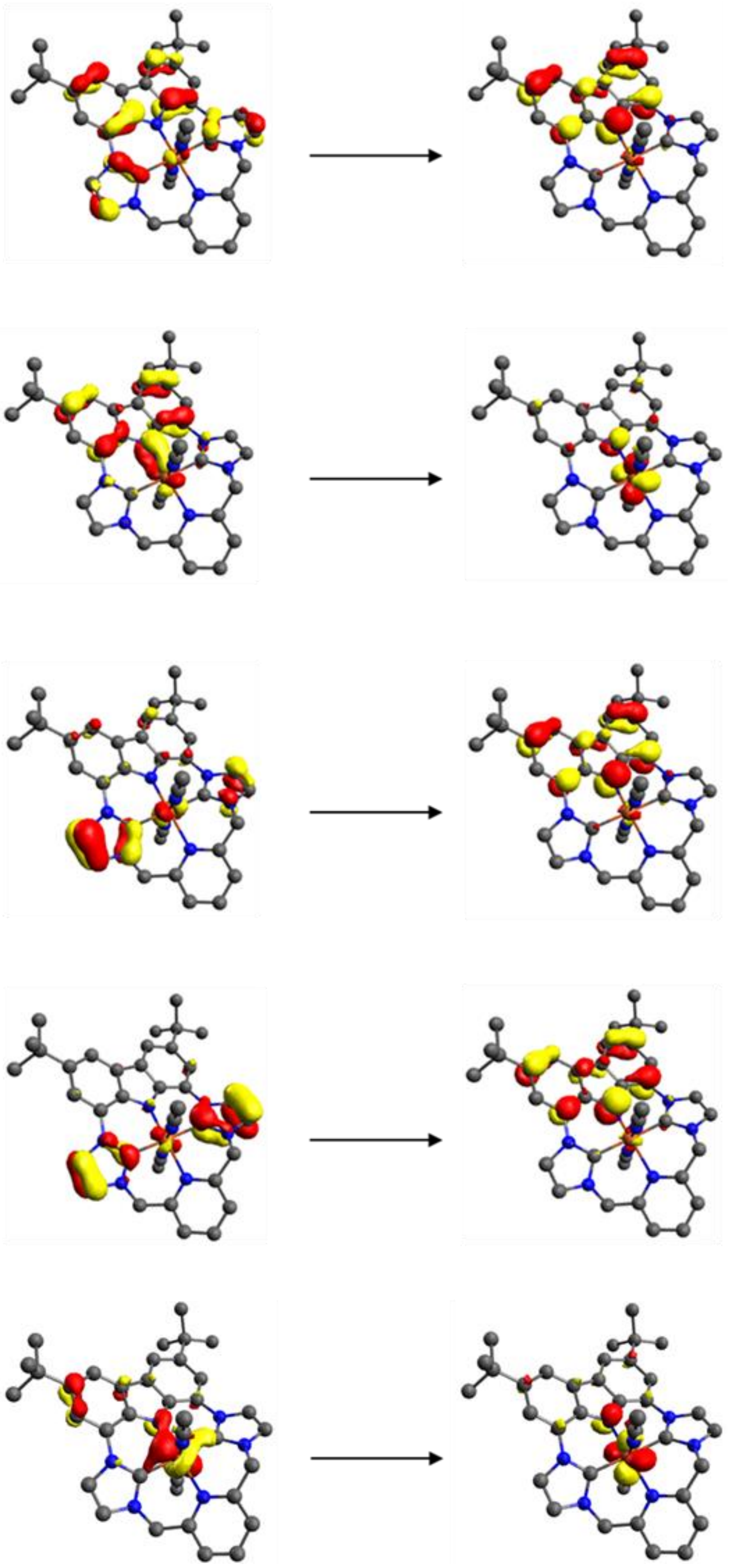

Figure S105. *Natural transition orbitals that mainly contribute to states 4, 5, 6, 8 and 12 (from top to bottom) for complex* ***4a****.*

10.6. Complex **4b**

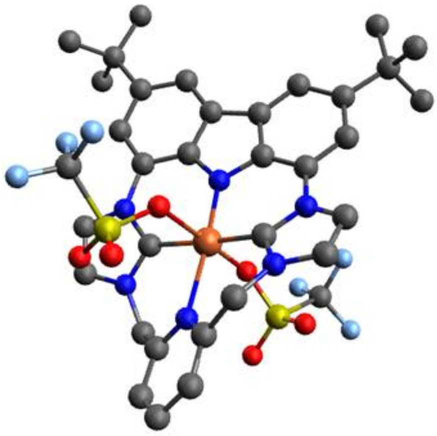

Figure S106. *Visualization of the optimized geometry of the cation of complex* ***4b*** *based on DFT calculation at the BP86 level (grey: C, blue: N, red: O, yellow: S, light blue: F, orange: Fe; hydrogen atoms omitted for clarity).*

Table S10. *Electronic energies and multiplicity of different ground states of* ***4a*** *calculated at the B3LYP level with variable fractions of Hartree–Fock exchange (HFX).*

**HFX [%]**	**15**		**20 (B3LYP)**	
Multiplicity	*E* [H]	Δ*E* [kcal mol^{-1}]	*E* [H]	Δ*E* [kcal mol^{-1}]
Triplet	–4790.28275	1.6	–4792.21313	3.9
Triplet (BS)	–4790.28524	0	–4792.21930	0

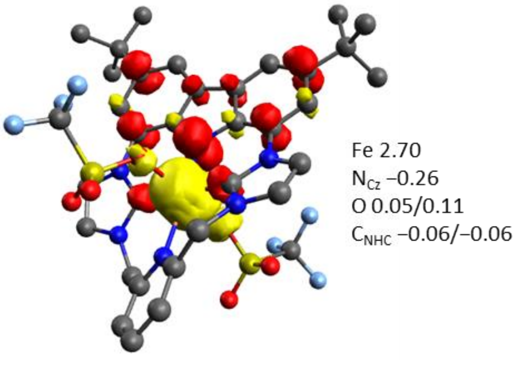

Figure S107. *DFT calculated spin density plot (isovalue 0.005) for complex* ***4b*** *at the B3LYP level. Spin distribution according to Löwdin population analysis.*

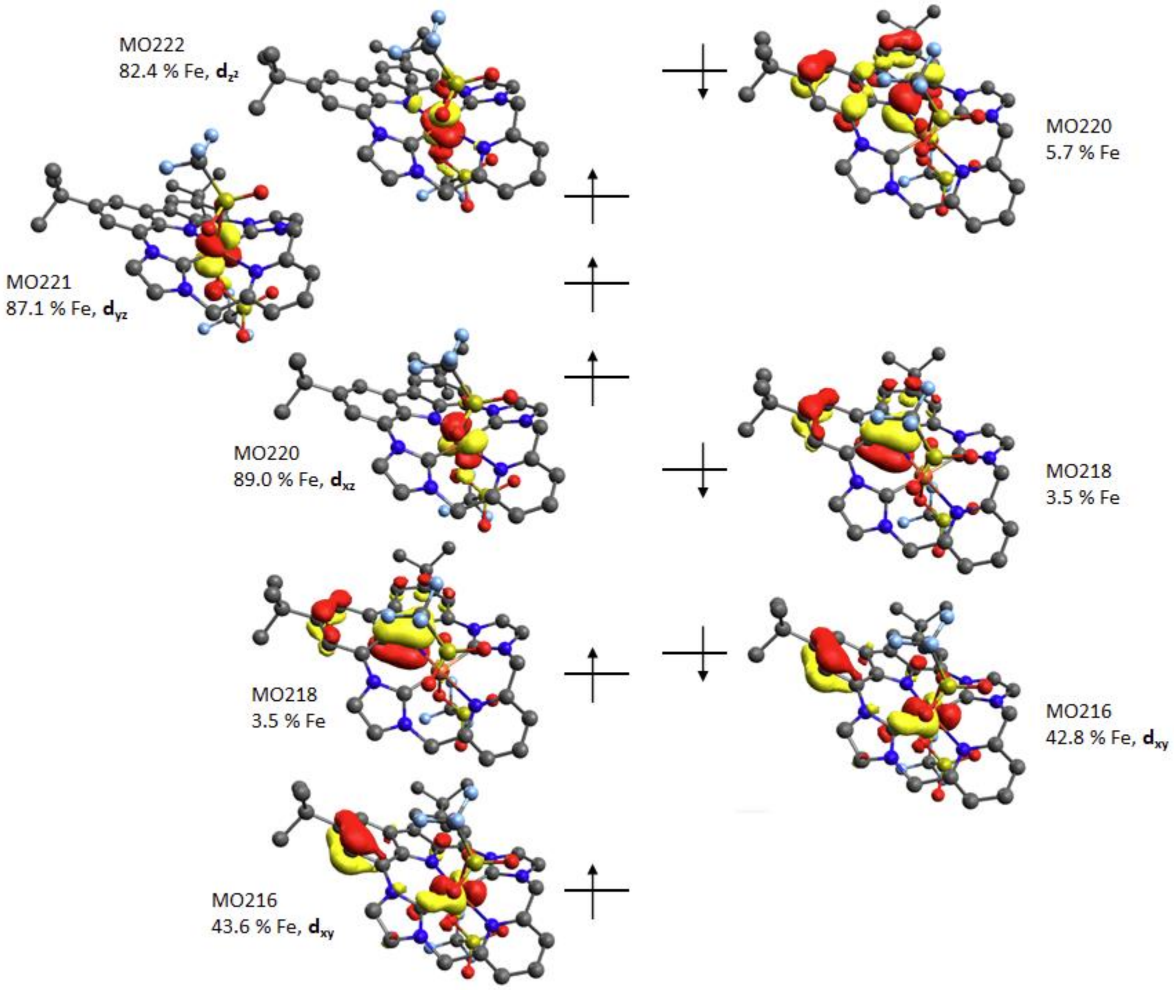

Figure S108. *Schematic molecular orbital diagram of* ***4b*** *represented by unrestricted corresponding orbitals (isovalue 0.05) at the B3LYP level. Iron contribution and atom orbital assignments are based on Löwdin reduced orbital populations per MO.*

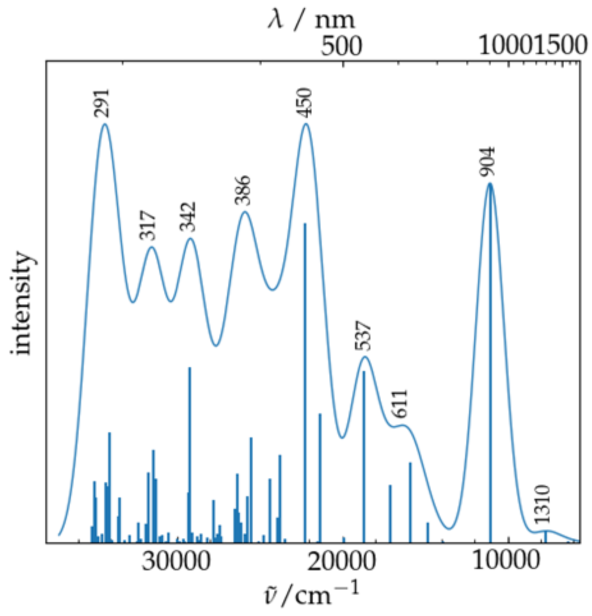

Figure S109. *Calculated absorption spectrum of the cation of complex* ***4b*** *in acetone at the B3LYP level.*

Table S11. *Selected TD-DFT (B3LYP) calculated energies, oscillator strengths and major contributions of the main electronic transitions of complex* ***4b****.*

States	Energy (cm^{-1})	Wavelength (nm)	Osc. Strength	Major Contributions
4	11057.8	904.3	0.126192648	HOMO−1 → LUMO (94 %)
6	14831.6	674.2	0.007023514	HOMO−2 → LUMO (95 %)
8	15896.2	629.1	0.028148684	HOMO−3 → LUMO (93 %)
9	17084.4	585.3	0.020438760	HOMO−4 → LUMO (88 %)

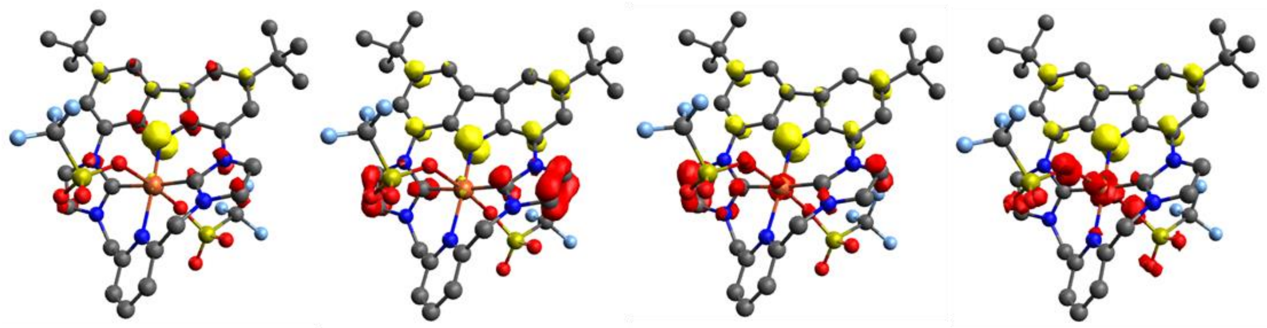

Figure S110. *Calculated TD-DFT difference density of complex* ***4b*** *(yellow/red indicates gain/loss of electron density) for states 4 (904 nm), 6 (674 nm), 8 (629 nm) and 9 (585 nm).*

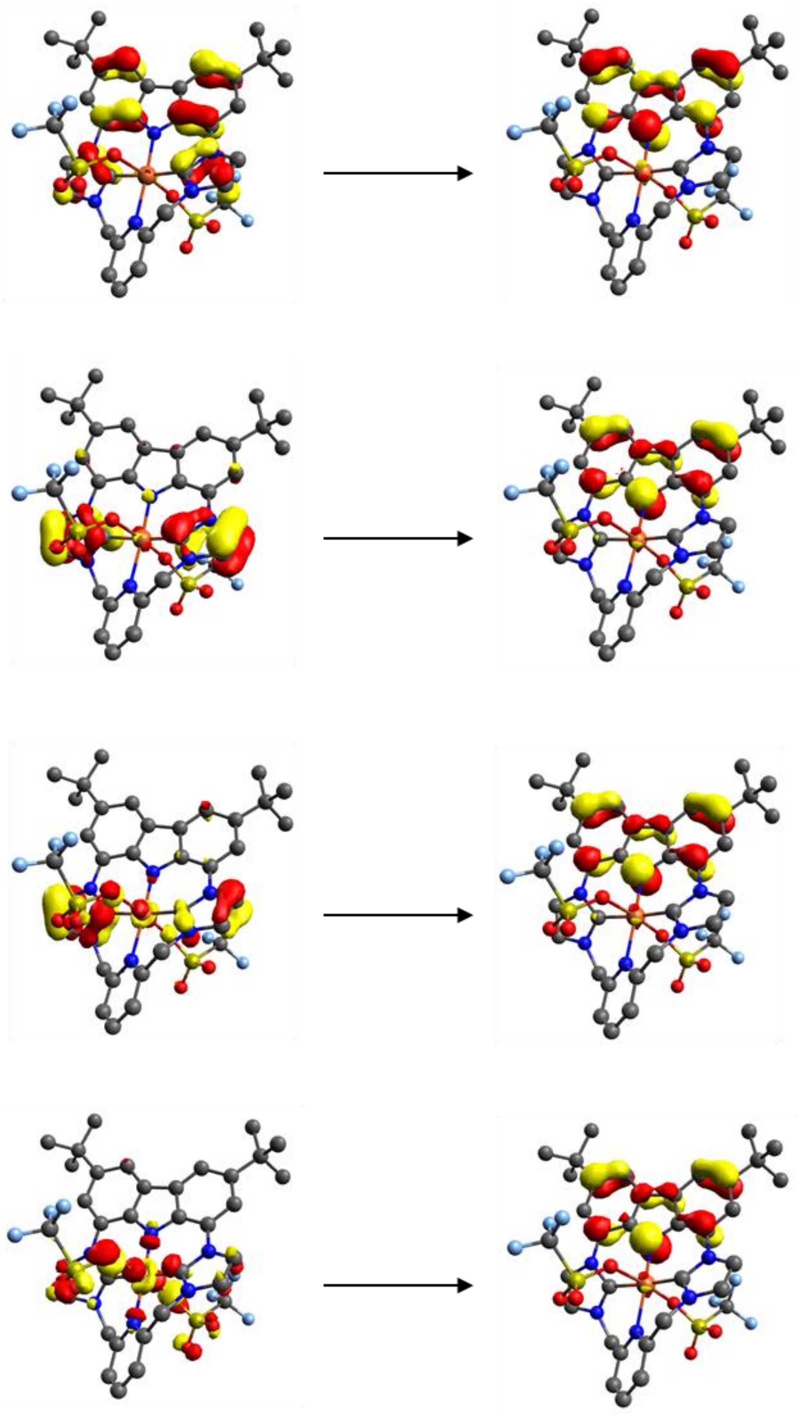

Figure S111. *Natural transition orbitals that mainly contribute to states 4, 6, 8 and 9 (from top to bottom) for complex* ***4b****.*
